# Attitudes towards career choice and general practice: a cross-sectional survey of medical students and residents in Tyrol, Austria

**DOI:** 10.1186/s12909-024-05205-8

**Published:** 2024-03-15

**Authors:** Angelika Mahlknecht, Adolf Engl, Verena Barbieri, Herbert Bachler, Alois Obwegeser, Giuliano Piccoliori, Christian J. Wiedermann

**Affiliations:** 1 Institute of General Practice and Public Health, College of Health Care Professions, Lorenz-Boehler-street 13, Bolzano, 39100 Italy; 2 Institute of General Practice, Medical University Innsbruck, Christoph-Probst-square 1, Innsbruck, 6020 Austria; 3 Department of Neurosurgery, University Hospital of Innsbruck, Anich-street 35, Innsbruck, 6020 Austria; 4 UMIT – Private University for Health Sciences, Medical Informatics and Technology – Tyrol, Eduard- Wallnöfer-center 1, Hall in Tirol, 6060 Austria

**Keywords:** General practice, Primary health care, Career choice, Graduate medical education, Undergraduate medical education, Curriculum, Cross-sectional survey

## Abstract

**Background:**

The global primary healthcare workforce is declining, leading to a shortage of general practitioners. Although various educational models aim to increase interest in general practice, effective interventions are limited. The reasons for this low appeal among medical graduates remain unclear.

**Methods:**

This cross-sectional study surveyed medical students’ and residents’ attitudes towards general practice in Tyrol, Austria. The online questionnaire addressed professional values, general practice-related issues, personal professional intentions, and demographics. Data analysis employed chi-square tests and multivariate logistic regression to explore predictors of interest in general practice.

**Results:**

The study included 528 students and 103 residents. Key values identified were stable positions, assured income, and work-family reconciliation. General practice was recognised for long-term patient relationships and patient contact, with students attributing more positive work-environmental characteristics and higher reputation to it than residents. Few participants (students: 3.2%, residents: 11.7%) had opted for general practice; about half were considering it as career option. Reasons not to choose general practice were preferences for other specialties, intrinsic characteristics of general practice, workload, insufficient time for the patients, financial pressures, low reputation, and perceived mediocre training quality. Predictors of interest in general practice included perception of independent decision-making, importance of work-family balance (students), better practical experiences in general practice during medical school (students and residents), younger age, and perceiving general practice as offering a promising future (residents). Both groups felt underprepared by medical school and/or general practice training for general practice roles. The attractiveness of specialist medicine over general practice was related to clearer content boundaries, better career opportunities, and higher incomes.

**Conclusions:**

According to these results, measures to improve attractiveness of general practice should focus on (i) high-quality undergraduate education including practical experiences, and (ii) on ensuring professional autonomy, work-family reconciliation, and job stability. Efforts to encourage more graduates to pursue this essential healthcare sector are crucial for strengthening primary healthcare and public health services.

**Trial registration:**

The study has not been registered as it did not include a direct medical intervention on human participants.

**Supplementary Information:**

The online version contains supplementary material available at 10.1186/s12909-024-05205-8.

## Background

A well-functioning primary healthcare (PHC) sector is an essential feature of a high-performing healthcare system [1]. Strengthening PHC has shown to improve various outcomes, including morbidity and mortality, life expectancy, health status in the general population [2, 3], reduced avoidable hospitalisations and healthcare expenditures [2, 4]. General practice plays a key role in chronic care [4] and in providing essential public health services, as highlighted by the COVID-19 pandemic [1, 5]. However, PHC workforces are declining globally [6–9] and a shortage of general practitioners (GPs) is especially imminent in rural areas [10]. Working as a GP seems to lack attractivity for medical students [11], and the percentage of medical graduates orientating themselves towards general practice is far below the required number [12].

Career choices and their determinants among medical graduates have been studied previously [12, 13], but effective interventions addressing both under- and postgraduate education to increase interest in general practice have not yet been widely implemented. Further insights could therefore provide valuable information regarding aspects which should be chiefly considered by specific interventions.

General practice education models and implementation in medical school curricula vary internationally [10, 14, 15]. While general practice as a subject was absent in former medical school curricula, students in Austria are now exposed to general practice experiences throughout their educational path, e.g., clinical case demonstrations, various facultative general practice teachings, and the possibility to complete parts of the mandatory clerkship and clinical practical year in a GP office [16].

GP residents in Austria as in other countries [7] were formerly exclusively trained in hospital settings; from 2015 up to now, after completing basic education and hospital internships, they receive mandatory nine-month training in a GP office where they work alongside an experienced GP mentor [17].

While these measures should have improved interest and self-confidence of young doctors to work in PHC [7], the reasons for the persisting low attractiveness of general practice among Austrian medical graduates remain unclear.

The present study, conducted in Tyrol, Austria, had the following aims:

1. to assess the general values and expectations of medical students and residents regarding their future professional lives,
2. to investigate the participants’ current perceptions and attitudes towards general practice, their general interest and previous experiences in PHC,
3. to assess participants’ personal intentions and preferences with respect to different possible professional profiles and working conditions,
4. and to identify demographic variables and other factors that positively or negatively influence interest in general practice as a career choice among the medical students and residents surveyed.

The study hypothesised that a potentially low interest in general practice may be due to various factors, including insufficient practical primary care experiences during medical school and a lack of attractive training and working circumstances.

## Methods

### Study design and setting

This cross-sectional study was conducted as an online survey in two cohorts:

i. The survey addressing the currently enrolled medical students of all years at the Medical University of Innsbruck (Austria) was conducted between 2022-05-20 and 2022-07-18.
ii. The survey of residents attending medical training (internship, ‘Turnus’, or residency) in nine different hospitals of Tyrol (Austria) was conducted between 2022-06-24 and 2022-09-03.

The surveys were administered at different times to accommodate coordination with multiple medical directorates for residents and to precede the university’s summer holidays for students. The time slot of the residents’ survey was extended owing to a low response rate during the summerly vacation period.

In this study, ‘residents’ refers to medical doctors in various stages of postgraduate training that may lead to general practice or other specialties. By deliberately including a diverse group of residents (undecided, in basic training, or aspiring to a specific specialty), a comprehensive assessment of attitudes towards PHC was undertaken to gather a broad range of insights that reflect the general disposition towards general practice in the medical community of young physicians, not only the view of those who are already committed to PHC.

The study aimed to include all currently active medical students and residents of Tyrol (inclusion criterium). No explicit exclusion criteria were applied. As the entire population was targeted, there was no need to perform a sample size calculation. Although the entire target population can provide comprehensive insights, it may still introduce some biases, including non-response and selection biases.

### Recruitment

The recruitment of the study participants was carried out by the vice-chancellorship of the Medical University Innsbruck and medical directorates of the participating hospitals. The link to the online questionnaire along with a short information letter containing information about the study, its purpose and information regarding the processing and protection of the collected data was sent to all medical students then enrolled at the Medical University Innsbruck (*n* = 3,453) and to all residents who were then employed at the participating hospitals (*n* = 578). For both cohorts, an email reminder was sent three weeks after the first invitation.

### Online questionnaire

The research team developed the questionnaire through a consensus process. The included variables and measures of the survey were informed by a combination of theoretical frameworks and empirical evidence that highlighted key factors influencing career choice in the healthcare sector. The approach was grounded in Social Cognitive Career Theory, which emphasised the role of personal performance beliefs and outcome expectations in shaping career decisions [18]. Furthermore, the Theory of Planned Behaviour informed the inclusion of variables, relating to attitudes toward behaviour, subjective norms, and perceived behavioural control, suggesting that these components collectively influence career paths [19]. In addition, the inclusion of variables was based on empirical studies of medical career choice that have identified work-life balance, income expectations, and the perceived prestige of a specialty as key factors [20, 21].

The questionnaire comprised 122 items (for the students) and 124 items (for the residents), respectively, and involved previously published survey instruments (part A-C) as well as ad-hoc developed questions (part D). The total questionnaire consisted of the following parts:

- Part A included general professional values and expectations rated as important or less important by the participants (addressing the study aim 1).
- Part B included the same and some additional issues as Part A in relation to the work as a GP (perceived values and characteristics of general practice; addressing the study aim 2).

The parts A and B corresponded to a validated questionnaire of the Department of General Practice and Health Services Research of the University Hospital Heidelberg, Germany, which was developed to assess the views of PHC among medical students [22].

- Part C (addressing the study aims 2 and 3) comprised questions regarding personal professional notions and intentions, former experiences in general practice, perceived preparedness to work as a GP, and attitudes towards general practice in comparison with specialism, corresponding to selected items of a questionnaire from the Institute of General Medicine and Evidence-based Health Services Research at the Medical University of Graz, Austria [10] which was developed to investigate the professional motivation of medical students and residents regarding general practice.
- Part D addressed demographic information (study aim 4). The items of part D were developed ad-hoc by the research team.

The medical students answered the same questions as the residents, with exception of two items which concerned only the residents (perceived preparedness to work as a GP by the general practice training, part C; current department/specialty of deployment, part D).

The answer scales were mostly ordinal (predominantly five-point); some questions allowed free-text entries. The time needed to complete the online questionnaire was about fifteen minutes.

Permission to use preexisting questionnaires (parts A-C) was obtained from the authors prior to the start of the study. The questionnaire was programmed in German using the online survey tool ‘Q-set’ (www.q-set.de). Participation was anonymous, the survey tool assigned a pseudonymisation codex to each participant; an identification of single participants by the research team was not possible at any time.

The responses to the online questionnaire were automatically imported into a csv-datafile by the survey tool (resulting in two different datafiles according to the two study cohorts) which were subsequently exported by the research team for the statistical analysis.

### Statistical analysis

Data were analysed using IBM®SPSS®Statistics 27.0. Only completed questionnaires were considered for analysis; in case of single missing responses the concerned individuals were excluded from the analysis of the respective item. Nominal and ordinal data were calculated as absolute (n) and relative (%) frequencies. Free-text comments were categorised and descriptively summarised.

Chi-square tests were used for comparison between the two study cohorts and for subgroup analyses. All tests were two-sided, a significance level of *p* < 0.05 was used throughout.

Furthermore, we conducted a multivariate logistic regression analysis to explore potential predictors of three dependent variables regarding the participants’ interest in PHC: (i) currently preferred specialty choice, (ii) interest in general practice as a specialty for the future professional life, and (iii) attractiveness of becoming a GP. For this purpose, all concerned dependent and independent variables using a five-point Likert answer scale were dichotomised as follows: 1 = ‘yes, surely’ or ‘rather yes’; 0 = ‘neutral, ‘rather no’ or ‘not at all’. The variables with a 6-point answer scale were dichotomised accordingly (1 = ‘very good’ or ‘good’; 0 = neutral’, ‘bad’, ‘very bad’ or ‘no experience’). The variable currently preferred specialty choice was dichotomised into 1 = ‘only general practice’ or ‘general practice or other specialty’ and 0 = ‘only other specialty’ or ‘don’t know’.

Chi-Square tests were used to explore the association of the three dependent variables with possible predictors. All resulting significant predictors were tested for correlations between each other using Phi and Chi-square tests and excluded from the logistic regression model when they were highly correlated with another predictor (correlation coefficient > 0.35). Subsequently, the logistic regression models were calculated. For all significant independent variables, the odds ratio (OR) and corresponding 95% confidence intervals (CI) were calculated. Model fit was assessed using Nagelkerke’s R^2. Sample size estimation for a two-tailed logistic regression model required 103 participants with a type one error of 5%, a power of 80% and a significant OR of 1.83. Significance levels are expressed as *p* < 0.05 (^*^), *p* < 0.01 (^**^) and *p* < 0.001 (^***^).

For reporting, we adhered to the STrengthening the Reporting of OBservational studies in Epidemiology (STROBE) Checklist.

## Results

### Study participants: demographic information

In total, *n* = 528 medical students (response: 15.3%) and *n* = 103 residents (response: 17.8%) participated in the survey.

About two-thirds (67.8%) of the participating students were 23 + years old and the majority of the participating residents (62.1%) was below 30 years old. The gender-related distribution was nearly similar in the two cohorts (female: 56.3% of the students, 57.3% of the residents; male: 43.7% of the students, 42.7% of the residents). More than half of the participants were Austrian (54.1% of the students, 59.2% of the residents), less than one-fifth were German (18.7% of the students, 15.5% of the residents); 23.6% of the students and 20.4% of the residents were from the northern-Italian Province of Bolzano (South Tyrol) which geographically approximates closely to Innsbruck. More than 90% of the participants indicated that German was their primary language. Most of the residents had graduated from the Medical University of Innsbruck (81.4%). On average, students were in their fourth year of study (median: eighth semester). All characteristics of the study participants are shown in Table 1.

**Table 1:** Demographic information and characteristics of the study participants

Characteristics	Medical students	Residents
Age groups	*n* = 513	*n* = 103
< 23 years: n (%)	165 (32.2%)	–
≥ 23 years: n (%)	348 (67.8%)	–
< 30 years: n (%)	–	64 (62.1%)
≥ 30 years: n (%)	–	39 (37.9%)
**Gender**	*n* = 526	*n* = 103
Female: n (%)	296 (56.3%)	59 (57.3%)
Male: n (%)	230 (43.7%)	44 (42.7%)
**Nationality**	*n* = 525	*n* = 103
Austria: n (%)	284 (54.1%)	61 (59.2%)
Germany: n (%)	98 (18.7%)	16 (15.5%)
South Tyrol: n (%)	124 (23.6%)	21 (20.4%)
Other Italian region (except South Tyrol): n (%)	1 (0.2%)	0 (0.0%)
Other EU-state: n (%)	8 (1.5%)	3 (2.9%)
Non-EU-state: n (%)	10 (1.9%)	2 (1.9%)
**Mother language**	*n* = 525	*n* = 102
German	500 (95.2%)	95 (93.1%)
Italian	4 (0.8%)	0 (0.0%)
Other	21 (4.0%)	7 (6.9%)
**Current semester of study**	*n* = 520	–
Median (IQR)	8.0 (4.0–10.0)	–
**University of medical graduation**	– ^**§**^	*n* = 102
Medical University of Innsbruck: n (%)	–	83 (81.4%)
Other Austrian University: n (%)	–	8 (7.8%)
German University: n (%)	–	9 (8.8%)
Other University: n (%)	–	2 (2.0%)
**Current department / specialty of employment** ^**§§**^		*n* = 96
Internship (‘Turnus’) / basic education	–	32 (33.4%)
General Practice	–	28 (29.2%)
Internal Medicine	–	8 (8.3%)
Anaesthesia	–	6 (6.3%)
Paediatrics	–	4 (4.2%)
Neurology	–	3 (3.1%)
Otolaryngology	–	3 (3.1%)
Psychiatry	–	2 (2.1%)
Gynaecology	–	2 (2.1%)
Urology	–	2 (2.1%)
Surgery / Vascular Surgery		2 (2.1%)
Ophthalmology	–	1 (1.0%)
Dermatology	–	1 (1.0%)
Orthopaedics / Traumatology	–	1 (1.0%)
Radiology	–	1 (1.0%)

*IQR* Interquartile range. ^ii^ Chi² Test. ^**§**^ Only students studying at the Medical University of Innsbruck were addressed by this survey. ^**§§**^ Corresponding to the current department of medical training and not necessarily to the definitively chosen medical specialty

### General wishes and aspired values in the professional life and in relation to general practice (parts A and B of the questionnaire)

*Most important professional values and expectations*: The most frequently mentioned values were working in a profession with a promising future (‘yes, surely’: 81.5% of the students, 74.8% of the residents), having a stable working position (73.6% of the students, 70.9% of the residents), an assured income (73.6% of the students, 68.6% of the residents), being able to reconcile family and professional life (66.7% of the students, 68.9% of the residents), being able to realise private aims (64.8% of the students, 62.1% of the residents), and to work according to the scientific state of the art (60.6% of the students).

*Most frequently perceived values and characteristics of general practice*: Long-term relationships with patients (‘yes, surely’: 85.0% of the students, 74.8% of the residents), much contact with patients (85.0% of the students, 74.8% of the residents), preventive activities (81.9% of the students, 73.5% of the residents), making independent, self-reliant decisions (64.0% of the students, 71.8% of the residents), PHC being a profession with a promising future (62.9% of the students, 52.4% of the residents) and offering a stable working position (62.3% of the students, 53.4% of the residents).

#### Correspondence between important professional values and perceived characteristics of PHC

More than 70% of the participants gave a high importance to working in a profession with a promising future and to an assured working position. At the same time, more than half of the residents and over 60% of the students attributed these preconditions to general practice. The application of broad medical knowledge was rated as more important by residents (57.8%) than by students (47.9%); however, the latter attributed this characteristic more frequently to general practice (57.2%) than the residents (45.6%).

#### Comparison between students and residents regarding professional values

The students gave higher importance to working in a profession with a promising future, having a diversified daily routine, participation in research, to work according to the scientific state of the art, and public appreciation. The residents attributed a significantly higher importance to regular working times and few night duties.

#### Comparison between students and residents regarding perceptions of PHC

The students more frequently perceived general practice as offering flexible and regular working times, few night duties, assured income, participation in research, a low physical and psychological burden, much time for recreation, separability of professional and private life, family-work balance, realisation of private aims, public appreciation, positive perception in the medical community and in the media, and having a high importance during medical education. The residents more frequently saw a diversified daily routine as characteristic of general practice.

The complete results regarding professional values and perceived characteristics of general practice (part A and B of the questionnaire, comparison between students and residents) are listed in the Supplementary Table S1. Subgroup analyses according to gender, age, and nationality are shown in the Supplementary Tables S2-S7.

### Interest and former experiences in general practice, perceived preparedness to work as a GP, and general practice versus specialism (part C of the questionnaire)

*General interest in PHC* as a career choice was expressed by 39.6% of the students and 55.9% of the residents (‘yes, surely’ and ‘rather yes’), whereas 3.2% of the students and 11.7% of the residents had already decided to become a GP (‘only general practice’) and 44.2% of the students and about half of the residents (49.5%) were at any rate not excluding PHC (‘general practice or other specialty’). The residents showed significantly higher interest in PHC than the students, whereas the latter were more often undecided about the specialty.

For the students, the most frequently mentioned *reasons not to choose general practice* as a career option were the preference of another specialty and the assumed characteristics and operating principles of general practice, whereas the residents most frequently indicted a high workload in PHC with insufficient time for a single patient, followed by financial responsibility, perceived low public appreciation, the need for quality improvement of general practice training, and likewise the preference of another specialty. The complete results are shown in Table 2.

**Table 2:** Interest in general practice as a career choice

		Only general practice		General practice or other specialty		Only other specialty		Don’t know	n	*p*-value	
Currently planned specialty choice											
Students		17 (3.2%)		233 (44.2%)		215 (40.8%)		62 (11.8%)	*527*	**< 0.001** ^**ii**^	
Residents		12 (11.7%)		51 (49.5%)		38 (36.9%)		2 (1.9%)	*103*		
		**Yes, surely**	**Rather yes**		**Neutral**		**Rather no**	**Not at all**	***n***	***p-value***	
**Interest in general practice as a specialty for the future professional life**											
Students		75 (14.3%)	133 (25.3%)		146 (27.8%)		122 (23.2%)	50 (9.5%)	*526*	**0.001** ^**ii**^	
Residents		32 (31.4%)	25 (24.5%)		20 (19.6%)		17 (16.7%)	8 (7.8%)	*102*		
								**Students**	**Residents**		
**Reasons for*not*being interested in general practice** [free-text answers]								*n* = 176 ^**§**^	*n* = 23 ^**§**^		
1.	Preference of another specialty							60 (34.1%)	5 (21.7%)		
2.	General practice is boring/monotonous, many bagatelle health problems, geriatric patients, long-term relationships with patients							43 (24.4%)	4 (17.4%)		
3.	Too broad spectrum, not specialised, preference of specific knowledge in one concrete specialty							33 (18.8%)	1 (4.3%)		
4.	Gatekeeper-function, referral in case of more complex or interesting conditions							29 (16.5%)	2 (8.7%)		
5.	Financial responsibility, lower income than a specialist							22 (12.5%)	5 (21.7%)		
6.	High burden and workload, no time for the single patient, long working hours							21 (11.9%)	7 (30.4%)		
7.	No teamwork, limited possibility of networking							20 (11.4%)	0 (0.0%)		
8.	Too limited possibility of diagnostic services in the GP office, some therapeutic procedures are not possible or not remunerated							19 (10.8%)	2 (8.7%)		
9.	Preference of the hospital setting as a working place							19 (10.8%)	1 (4.3%)		
10.	Free-lance activity and health insurance system are dissuasive							15 (8.5%)	1 (4.3%)		
11.	Bureaucracy, high administrative burden							11 (6.3%)	1 (4.3%)		
12.	Little appreciation of general practice, no specialist qualification							10 (5.7%)	5 (21.7%)		
13.	Too few possibilities to participate in research activities							9 (5.1%)	1 (4.3%)		
14.	Low quality of the general practice training, unstructured, little recognition							5 (2.8%)	5 (21.7%)		
15.	Low support of GPs by health policy makers, little networking with hospitals							5 (2.8%)	0 (0.0%)		
16.	Separation of professional and private life is difficult							2 (1.1%)	4 (17.4%)		
17.	Much responsibility, psychological burden, stress							1 (0.6%)	3 (13.0%)		
18.	General practice offers low professional perspectives							0 (0.0%)	3 (13.0%)		
19.	Too few possibilities of being employed by another GP							0 (0.0%)	2 (8.7%)		

*GP* General practitioner. ^§^ n = The number of students and residents who gave free-text answers

The quality of the *practical primary care experiences* during medical school was rated significantly higher by the residents (71.9% ‘very good’ or ‘good’) than by the students (38.6%), while the *theoretical experiences* were valued significantly better by the students (49.3% very good’ or ‘good’) than by the residents (25.3%).

Half of the participating residents indicated to feel rather not *prepared to work as a GP* and one-fifth felt not at all prepared by medical school. The students indicated significantly higher preparedness, however, also in this cohort, over one-third felt insufficiently prepared. The preparedness acquired by the residents during internship or general practice training was higher (51.0% felt well or rather well prepared); however, one-quarter indicated persistent insufficient preparedness to work as a GP.

The residents non-significantly more often indicated a *specialist qualification* as motivating for becoming a GP (57.3% ‘yes, surely’ or ‘rather yes’) than the students (46.7%).

The most frequently mentioned *reasons for a higher attractiveness of specialist medicine* compared to general practice were the ability to extend one’s knowledge in a more targeted way (‘yes, surely’ or ‘rather yes’: 74.3% of the students, 67.7% of the residents), the clearer content-related demarcations of a specialty (56.6% of the students, 57.8% of the residents), better opportunities of career as a specialist (57.3% of the students, 56.9% of the residents), a higher income of specialists (54.9% of the students, 57.4% of the residents), and more possibilities to participate in research (53.4% of the students, 41.2% of the residents). About half of the participants did not attribute a lower reputation to physicians without specialist qualifications while the residents more frequently perceived a physician without specialist qualification as a ‘lesser’ physician than the students. The results are shown in Table 3.

**Table 3:** Experiences in general practice, preparedness to work as GP, and specialism versus general practice

	Very good		Good		Neutral		Bad		Very bad	No exp.	n	*p*-value
**Quality of the practical experiences in general practice during medical school**												
Students	109 (20.7%)		94 (17.9%)		45 (8.6%)		13 (2.5%)		2 (0.4%)	263 (50.0%)	*526*	**< 0.001** ^**ii**^
Residents	36 (35.0%)		38 (36.9%)		21 (20.4%)		4 (3.9%)		3 (2.9%)	1 (1.0%)	*103*	
**Quality of the theoretical experiences in general practice during medical school**												
Students	56 (10.6%)		204 (38.7%)		174 (33.0%)		58 (11.0%)		15 (2.8%)	20 (3.8%)	*527*	**< 0.001** ^**ii**^
Residents	4 (3.9%)		22 (21.4%)		37 (35.9%)		28 (27.2%)		10 (9.7%)	2 (1.9%)	*103*	
		**Yes, surely**		**Rather yes**		**Neutral**		**Rather no**		**Not at all**	***n***	***p-value***
**I feel well prepared to work as a GP by medical school**												
Students		11 (2.1%)		121 (23.1%)		200 (38.2%)		162 (31.0%)		29 (5.5%)	*523*	**< 0.001** ^**ii**^
Residents		2 (1.9%)		5 (4.9%)		24 (23.3%)		52 (50.5%)		20 (19.4%)	*103*	
**I feel well prepared to work as a GP by the general practice training**												
Students		-		-		-		-		-	*-*	-
Residents		12 (12.0%)		39 (39.0%)		24 (24.0%)		18 (18.0%)		7 (7.0%)	*103*	
**A specialist qualification would increase the attractiveness of general practice**												
Students		86 (16.3%)		160 (30.4%)		88 (16.7%)		103 (19.6%)		89 (16.9%)	*526*	0.173 ^ii^
Residents		25 (24.3%)		34 (33.0%)		16 (15.5%)		18 (17.5%)		10 (9.7%)	*103*	
		**Yes, surely**		**Rather yes**		**Neutral**		**Rather no**		**Not at all**	***n***	***p-value***
**A specialist qualification is more attractive to me than general practice, because…**												
**… without any specialist qualification, I would be a ‘lesser’ physician**												
Students		28 (5.3%)		88 (16.8%)		67 (12.8%)		141 (26.9%)		201 (38.3%)	*525*	**0.040** ^**ii**^
Residents		12 (11.8%)		16 (15.7%)		17 (16.7%)		30 (29.4%)		27 (26.5%)	*102*	
**… I can get higher reputation as a specialist**												
Students		66 (12.6%)		119 (22.8%)		80 (15.3%)		108 (20.7%)		149 (28.5%)	*522*	0.222 ^ii^
Residents		13 (12.9%)		25 (24.8%)		21 (20.8%)		24 (23.8%)		18 (17.8%)	*101*	
**… teamwork is easier as a specialist**												
Students		63 (12.0%)		147 (28.1%)		107 (20.5%)		114 (21.8%)		92 (17.6%)	*523*	0.421 ^ii^
Residents		18 (17.6%)		28 (27.5%)		18 (17.6%)		25 (24.5%)		13 (12.7%)	*102*	
**… I have better possibilities to work in research**												
Students		116 (22.1%)		164 (31.3%)		81 (15.5%)		86 (16.4%)		77 (14.7%)	*524*	0.156 ^ii^
Residents		15 (14.7%)		27 (26.5%)		16 (15.7%)		23 (22.5%)		21 (20.6%)	*102*	
**… I have a higher income**												
Students		127 (24.3%)		160 (30.6%)		99 (18.9%)		79 (15.1%)		58 (11.1%)	*523*	0.691 ^ii^
Residents		30 (29.7%)		28 (27.7%)		18 (17.8%)		17 (16.8%)		8 (7.9%)	*101*	
**… there are clearer content-related demarcations of the field**												
Students		127 (24.2%)		170 (32.4%)		96 (18.3%)		84 (16.0%)		48 (9.1%)	*525*	0.275 ^ii^
Residents		25 (24.5%)		34 (33.3%)		26 (25.5%)		12 (11.8%)		5 (4.9%)	*102*	
**… I am facing more challenging medical topics**												
Students		115 (21.9%)		110 (21.0%)		111 (21.1%)		126 (24.0%)		63 (12.0%)	*525*	0.086 ^ii^
Residents		15 (14.7%)		17 (16.7%)		33 (32.4%)		27 (26.5%)		10 (9.8%)	*102*	
**… I have better opportunities of career**												
Students		136 (25.9%)		165 (31.4%)		100 (19.0%)		70 (13.3%)		54 (10.3%)	*525*	0.961 ^ii^
Residents		25 (24.5%)		33 (32.4%)		19 (18.6%)		16 (15.7%)		9 (8.8%)	*102*	
**… I can extend my knowledge in a more targeted way**												
Students		242 (46.0%)		149 (28.3%)		72 (13.7%)		39 (7.4%)		24 (4.6%)	*526*	0.079 ^ii^
Residents		32 (31.4%)		37 (36.3%)		20 (19.6%)		9 (8.8%)		4 (3.9%)	*102*	

*GP* General practitioner, *Exp* Experience. ^ii^ Chi² Test

### Personal preferences regarding various professional profiles and working circumstances (part C of the questionnaire)

The participants considered the following profiles as most attractive: working in a group office (‘yes’: 63.9% of the students, 78.2% of the residents), in the inpatient setting (68.9% of the students, 51.5% of the residents), in a multi*-*professional team (64.7% of the residents), as an employed physician (54.5% of the residents) or working as a GP in the PHC setting (53.9% of the residents).

The residents indicated a significantly higher preference of working in a group office, being employed by another physician, working in a multi-professional team, working as a GP in the primary care setting, working with insurances or approaching a non-medical career. The students significantly more often preferred the hospital setting and a scientific career.

Most participants were open to work in both urban and rural settings. The students significantly more often indicated to prefer the urban area than the residents, whereas the latter rather tended to prefer the rural location. The complete results are shown in Table 4.

**Table 4:** Personal preferences regarding various professional profiles and working circumstances

	Yes	No	Don’t know	n	*p*-value
**I would preferably work…**					
**… as an independent physician in a group office**					
Students	337 (63.9%)	99 (18.8%)	91 (17.3%)	*527*	**0.004** ^**ii**^
Residents	79 (78.2%)	6 (5.9%)	16 (15.8%)	*101*	
**… as an independent physician in a single-handed office**					
Students	232 (44.1%)	208 (39.5%)	86 (16.3%)	*526*	0.076 ^ii^
Residents	33 (32.4%)	51 (50.0%)	18 (17.6%)	*102*	
**… as a physician in the hospital setting**					
Students	361 (68.9%)	102 (19.5%)	61 (11.6%)	*524*	**0.003** ^**ii**^
Residents	53 (51.5%)	32 (31.1%)	18 (17.5%)	*102*	
**… as an employed physician in another physician’s office**					
Students	174 (33.3%)	252 (48.3%)	96 (18.4%)	*522*	**< 0.001** ^**ii**^
Residents	55 (54.5%)	24 (23.8%)	22 (21.8%)	*101*	
**… as a GP in a multi-professional team (e.g. Primary Healthcare Centre)**					
Students	223 (42.6%)	183 (34.9%)	118 (22.5%)	*524*	**< 0.001** ^**ii**^
Residents	66 (64.7%)	24 (23.5%)	12 (11.8%)	*102*	
**… as a GP in the primary care setting**					
Students	196 (37.5%)	207 (39.6%)	120 (22.9%)	*523*	**0.001** ^**ii**^
Residents	55 (53.9%)	37 (36.3%)	10 (9.8%)	*102*	
**… as a ward physician in a hospital**					
Students	113 (21.5%)	302 (57.5%)	110 (21.0%)	*525*	**0.006** ^**ii**^
Residents	9 (8.9%)	73 (72.3%)	19 (18.8%)	*101*	
**… as a school physician**					
Students	63 (12.0%)	402 (76.6%)	60 (11.4%)	*525*	0.082 ^ii^
Residents	20 (19.8%)	68 (67.3%)	13 (12.9%)	*101*	
**… as a public health officer**					
Students	61 (11.7%)	397 (76.1%)	64 (12.3%)	*522*	0.053 ^ii^
Residents	15 (15.0%)	65 (65.0%)	20 (20.0%)	*100*	
**… as a physician with insurance companies or health insurances**					
Students	22 (4.2%)	454 (86.5%)	49 (9.3%)	*525*	**0.042** ^**ii**^
Residents	10 (10.0%)	79 (79.0%)	11 (11.0%)	*100*	
**… in a scientific career**					
Students	163 (31.2%)	272 (52.0%)	88 (16.8%)	*523*	**0.029** ^**ii**^
Residents	20 (19.6%)	67 (65.7%)	15 (14.7%)	*102*	
**… in a non-medical career (e.g. pharmaceutical industry, economy)**					
Students	43 (8.2%)	412 (78.9%)	67 (12.8%)	*522*	**0.011** ^**ii**^
Residents	16 (15.7%)	67 (65.7%)	19 (18.6%)	*102*	
**I would like to work…**					
**… in a rural area**					
Students	371 (70.4%)	75 (14.2%)	81 (15.4%)	*527*	0.070 ^ii^
Residents	81 (81.0%)	7 (7.0%)	12 (12.0%)	*100*	
**… in an urban area**					
Students	424 (80.3%)	52 (9.8%)	52 (9.8%)	*528*	**0.001** ^**ii**^
Residents	66 (64.1%)	18 (17.5%)	19 (18.4%)	*103*	

*GP* General practitioner. ^ii^ Chi² Test

### Determining factors of interest in general practice as a career choice (logistic regression analysis)

The logistic regression analysis was conducted separately for students and residents. We used ‘currently planned specialty choice’, ‘interest in general practice as a specialty for the future professional life’, and ‘attractiveness of the GP profession‘ as dependent variables. Independent variables were demographic factors (age, gender, nationality), along with factors indicated by both study cohorts as important in professional life and/or as existing features of general practice (stable working position, promising future, assured income, family-work balance, realisation of private aims, independent decision-making, preventive activities, patient contact, and long-term patient relationships). Additionally, the quality of the practical and theoretical experiences in general practice during medical school were included as independent variables.

For the students, ‘*currently planned specialty choice*’ was significantly correlated with age group, ‘it is important to me to reconcile family and professional life’, ‘in general practice one makes independent decisions’, ‘general practice offers the possibility to conduct preventive activities’ and ‘quality of the practical experience’. All five variables were only slightly correlated with each other and were thus added to the logistic regression model. Medical students with higher interest in reconciliation of family and professional life (OR = 3.18, 95% CI 1.49–6.80, p = 0.003), a higher perception of general practice as a specialty in which independent decisions are taken (OR = 4.66, 95% CI 2.04–10.65, p < 0.001), and who reported better practical experiences in general practice during medical school (OR = 1.78, 95% CI 1.20–2.63, p = 0.004) were significantly more interested in choosing general practice as a specialty choice.

For the residents, ‘currently planned specialty choice’ was significantly correlated with lower age group and ‘it is important to me to realise private aims’. The variables were only slightly correlated. In the logistic regression model, only younger age group remained a significant predictor (OR = 0.38, 95% CI 0.16–0.89, *p* = 0.027), indicating that younger residents (< 30 years) were significantly more interested in general practice as a specialty choice than their older counterparts.

‘*Interest in general practice as a specialty for the future professional life*’ was significantly correlated with age group, ‘it is important to me to reconcile family and professional life’, ‘in general practice one makes independent, self-reliant decisions’ and ‘quality of the practical experience’ for the students, and with age group, ‘general practice is a profession with a promising future’ and ‘quality of the practical experience’ for the residents. All the predictors were only slightly correlated with each other.

Regarding the students, significantly higher interest in general practice was reported by those with higher interest in work-family balance (OR = 3.22, 95% CI 1.51–6.86, *p* = 0.002), with a higher perception of general practice as a speciality where self-reliant decisions are taken (OR = 4.76, 95% CI 2.09–10.88, *p* < 0.001), and by students reporting better practical experiences in PHC during medical school (OR = 1.83, 95% CI 1.23–2.70, *p* = 0.003). Age was no longer significant.

Regarding the residents, higher interest in general practice was again found among younger age groups (OR = 0.37, 95% CI 0.15–0.88, *p* = 0.025) and among those who perceived general practice as a profession with a promising future (OR = 3.90, 95% CI 1.09–14.03, *p* = 0.037).

‘*I deem the profession of a GP as attractive*’ was significantly correlated with ‘it is important to me to reconcile family and professional life’, ‘general practice is a profession with a promising future’, ‘in general practice one makes independent, self-reliant decisions’, ‘general practice offers the possibility to conduct preventive activities’, ‘in general practice one has much contact with patients’ and ‘quality of the practical experiences’ for the students, and with the age group, ‘it is important to me to reconcile family and professional life’, ‘general practice is a profession with a promising future’ and ‘quality of the practical experience’ for the residents. All the predictors were only slightly correlated with each other.

For the students, the profession of a GP was again significantly more attractive for those with higher interest work-family balance (OR = 2.36, CI 1.16–4.79, *p* = 0.018), with a higher perception of general practice as a specialty where self-reliant decisions are taken (OR = 6.20, CI 2.39–16.09, *p* < 0.001), and with better practical experiences in general practice during medical school (OR = 2.10, CI 1.44–3.07, *p* < 0.001).

Younger residents (OR = 0.22, CI 0.08–0.58, *p* = 0.002) and those reporting better practical experiences during medical school (OR = 4.57, 1.43–14.61, *p* = 0.010) attributed a significantly higher attractiveness to the profession of a GP than their fellow residents.

Gender and nationality were no significant predictors in all calculated models. The complete results of the logistic regression analysis are shown in Table 5.

**Table 5:** Logistic regression analysis: predictors of interest in general practice

	Currently planned specialty choice		Interest in general practice as specialty		Attractiveness of becoming a GP	
	Students	Residents	Students	Residents	Students	Residents
n	506	102	508	102	519	103
Nagelkerke’s R^2	0.118	0.106	0.119	0.197	0.145	0.376
	*OR* *(95% CI)*	*OR* *(95% CI)*	*OR* *(95% CI)*	*OR* *(95% CI)*	*OR* *(95% CI)*	*OR* *(95% CI)*
Age group	n.s.	0.38 (0.16–0.89) ^*^	n.s.	0.37 (0.15–0.88) ^*^	–	0.22 (0.08–0.58) ^**^
It is important to me to reconcile family and professional life	3.18 (1.49–6.80) ^**^	–	3.22 (1.51–6.86) ^**^	–	2.36 (1.16–4.79) ^*^	n.s.
It is important to me to realise private aims	–	n.s.	–	–	–	–
General practice is a profession with a promising future	–	–	–	3.90 (1.09–14.03) ^*^	–	n.s.
In general practice one makes independent, self-reliant decisions	4.66 (2.04–10.65) ^***^	–	4.76 (2.09–10.88) ^***^	–	6.20 (2.39–16.09) ^***^	–
General practice offers the possibility to conduct preventive activities	n.s.	–	–	–	n.s.	–
In general practice one has much contact with patients	–	–	–	–	n.s.	–
Quality of practical general practice experiences during medical school	1.78 (1.20–2.63) ^**^	–	1.83 (1.23–2.70) ^**^	n.s.	2.10 (1.44–3.07) ^***^	4.57 (1.43–14.61) ^*^

*GP* General practitioner, *n.s.* not significant. ^*^ Significant, *p* < 0.05. ^**^ Significant, *p* < 0.01. ^***^ Significant, *p* < 0.001. – The concerned variable was not included in the model

## Discussion

### Interest in becoming a GP

Interest in becoming a GP in this cohort was low among students (3.2%) and residents (11.7%), with residents showing greater interest. On the other hand, nearly half of the participants were at least not excluding general practice as a professional field. Other studies found higher [9, 23, 24] or similar rates [25] of students having definitively opted for general practice; similarly to our study, up to 50% of the students considered general practice as at least partly attractive [12, 26–28].

For the students, the strongest predictor of interest was the perception of general practice as a profession with independent decision making. Moreover, the importance of work-family balance and better practical experiences in general practice during medical school were significant predictors of higher interest.

Previous studies suggested a trend of older students being more likely to consider general practice than their younger counterparts [23, 25, 29]. However, in our sample as well as in other studies, students’ age [27], gender [30] and nationality were no significant predictors for being interested in becoming a GP.

Among the residents, younger age was a consistent predictor of higher interest in general practice; moreover, better practical experiences in PHC during medical school and perceiving general practice as a profession with a promising future were significant predictors of higher interest in PHC. As in the students’ cohort, gender and nationality showed no significant impact.

Conspicuously, most of the identified factors affecting the attractiveness of general practice are issues which could be influenced by appropriate measures. Two main target areas can be distinguished in this regard:

i. High-quality education in general practice during medical school with a focus on practical experiences [25], knowledge about the profession’s characteristics, especially regarding independent, autonomous decision-making. Previous studies have emphasised the importance of medical students being early and continuously exposed to high-quality general practice experiences and to inspiring role models [11, 23, 26, 27, 30–34]. While in the UK about 12% of the medical school curriculum is taught by GPs [23], the currently mandatory general practice part at the Medical University of Innsbruck is considerably lower and amounts about 3% with however offering the possibility to optionally increase it up to 17%. To additionally increase the orientation of medical graduates towards PHC, the Medical University of Innsbruck recently introduced a new expanded four-semester curriculum for general practice which is completed within the ordinary medical school curriculum. Graduates of this expanded curriculum receive a certificate but no additional academic degree. The contents comprise theoretical knowledge, practical skills, internship in PHC and GP offices, acquaintance of the way of working and practice organisation, research, and freely selectable lectures with reference to general practice [35]. Future evaluations will investigate if and to which extent the introduction of this expanded curriculum may increase the willingness of young physicians to work as a GP. As a previous study has shown, students are expecting teaching programmes with high practical relevance, also regarding information about administrative and economic aspects when starting a GP office [36].
ii. Furthermore, our results highlight the importance of independence, a promising professional future, and ensured family-work balance. As previous studies confirm, the imposition of manyfold external directives highly contributes to a negative view of PHC) [10, 12]. Health policymakers should prioritise these aspects when undertaking measures relating to the working circumstances in PHC. Though gender in our cohort did not play a significant role regarding interest in PHC, other studies found more frequent career intentions towards general practice among female students [24, 37] but this trend was not confirmed elsewhere [30]. A tendency was noted of medical graduates to prefer an employment rather than the entrepreneurial GP activity with its potentially associated risks [37, 38]; this was more pronounced among female residents [8] and was also mentioned in our cohort. Increasing employment options in primary care, especially for young GPs [39], alongside facilitating job-family reconciliation, e.g. by offering part-time options and flexible working models, could make general practice more interesting for newcomers.

### Professional values and perceptions of general practice

Professional values and perceptions of general practice were found to prioritise a promising future and job security, stable income and work-family balance. Discipline-specific aspects such as diverse daily routines and scientific advancements were considered less important. Students valued discipline-specific aspects higher than residents, who prioritised regular working hours and fewer night duties.

The perceived working circumstances of general practice were only partially aligned with the most important professional values. As in previous findings [12], job security and work-life balance in general practice were positively perceived, but improvements in income security and family-life reconciliation were needed, indicating the potential for tailored health policy measures to improve PHC attractiveness [38].

About half of the students and residents perceived general practice as applying a broad medical knowledge, which in turn was an important professional value for half of the participants. This shows that PHC besides from several attractive working circumstances also offers discipline-specific characteristics which are appealing to young physicians and students.

Regarding the most frequent reasons for not wishing to become a GP, the residents mainly mentioned working-related characteristics which were previously confirmed [10, 12, 38], i.e., a high workload, limited time for patients, financial responsibility, low public reputation, and mediocre training quality.

A considerable part of the students and residents perceived general practice and its assumed intrinsic characteristics as monotonous (long-term patient relationships, frequent contact with geriatric patients and/or bagatelle health problems, broad spectrum, gatekeeper-function). These negative perceptions of the generalist characteristics were also found among other student cohorts [7, 12, 32, 38]; however, as confirmed previously [7, 32, 38], some students found diagnostic challenges, variety, dealing with uncertainty and risk, and long-term patient relationships attractive. The complexity of ambulatory care measured according to the quantity of information and events and diversity has shown to be higher in PHC than in other specialties [40]. This highlights potential misconceptions regarding the interestingness and intellectual challenges of general practice which should be addressed through intensive, challenging undergraduate exposure to the field [11].

A previously described ‘hidden curriculum’ in medical schools, i.e. unofficially and usually unintentionally taught values and lessons, is often not favourable towards general practice and can affect students’ PHC perceptions [11, 12, 41], suggesting a need to promote a respectful atmosphere towards PHC at medical schools, to which also the teaching GPs themselves can contribute by positively representing the field [38].

Interestingly, students valued general practice more positively than residents in terms of working circumstances but were less interested in pursuing it as a career. This suggests that, among students, content-related characteristics of a specialty play a more significant role regarding its attractiveness than work-environmental factors. Intensified teaching of general practice content and skills throughout the curriculum could improve the knowledge about the specifics of general practice and thus increase interest especially among undecided students [42]. The fact that the preparedness to work as a GP after medical school was perceived as low seems to confirm this necessity. It might be particularly important to give students in more advanced stages, when organisational and working circumstances become more important, a realistic, positive insight into the working conditions of PHC.

### Specialist qualification and preferred work settings

Introducing a specialist qualification for GPs in Austria [43] could make general practice more attractive by equalising GP qualifications with other medical specialties. However, according to our results, specialist qualifications may not be the most crucial factor in increasing the number of GPs.

Intrinsic factors such as the opportunity for a more targeted expansion of medical knowledge were the most common reasons for preferring a specialty other than general practice. About half of the participants found a specialty more attractive due to better career options, higher income and research opportunities, but not because of a higher reputation. Recent studies confirm that prestige though having an impact on career choices [25, 32] seem not to be among the most important driving forces [29], while financial aspects have shown to affect career choices [9, 25, 32]; however, other studies found that job satisfaction and workload balance were equally [32] or more important regarding GP recruitment than financial incentives [31].

Previous studies suggest that PHC is seen as offering limited research opportunities [12, 23, 29]. Establishing institutional departments of general practice at universities could increase students’ interest in primary care, potentially due to more established exposure and contact with scientifically active role models [37]. Consistent academic representation of general practice at medical schools and throughout the curriculum is crucial for improving its scientific reputation among students and graduates interested in research activities.

The students in our cohort generally preferred the hospital setting, whereas residents showed higher interest in working in group offices, as employed physicians, or in multi-professional teams. These preferences are supported by other studies [37, 39], suggesting that promoting group offices, teamwork, and employment options could increase the appeal of general practice for young physicians.

Interestingly, contrary to other studies [44], participants in this cohort showed a high willingness to work in rural areas, especially residents. This promising result may encourage health policymakers to invest in endorsement measures and thus ensure the continuity of care in rural areas. Initiatives targeting rural general practice promotion, such as financial grants and longitudinal programs, have been implemented in some regions [45] but their success in increasing GP careers in rural areas requires further investigation.

### Strengths and limitations

The survey used a validated instrument and included the views of both medical students and residents from nine different hospitals and various nationalities, offering a broader perspective on the reasons for choosing or refusing general practice. However, the cross-sectional design did not allow for conclusions regarding temporal development and causality.

The response rate was relatively low compared to that of other surveys [9, 12, 27, 28, 36], potentially compromising the representativeness of the results. However, other studies achieved similar response rates [25, 37] and the absolute number of medical students participating in our study was comparable to or higher than in other surveys [12, 23, 27, 36].

Generalisability is further limited by including participants of a specific Austrian region and by focusing on a single medical university. Nevertheless, the findings are supported by other Austrian and international studies, suggesting some validity and potential applicability in other contexts.

Selection bias may be present because of the overrepresentation of participants interested in GP careers. Older students (aged 23 + years) were more represented than younger students in our cohort, which might be due to a higher amount of undergone exposure to general practice contents and experiences in advanced stages of medical school and thus to a higher willingness to participate in a survey addressing perceptions of primary care. Gender representation in the study sample was comparable to the underlying population and similar surveys [29], but the study did not compare other demographic variables, so over- or under-representation of certain groups cannot be excluded. This may have influenced the identified attitudes towards general practice but should not have substantially affected the identification of determinants that might influence these attitudes.

For the logistic regression analysis, a dichotomisation of the Likert scale was conducted which distinguished between explicitly positive responses (e.g. ‘yes, surely’ or ‘rather yes’) and all other response options. The arbitrary allocation of the ‘neutral’ responses to the negative response group may be controversial, because a different way of dichotomisation by adding the ‘neutral’ responses to the positive response group could have impacted the results. However, the mentioned approach was chosen as it was deemed appropriate and most meaningful to differentiate the explicitly positive responses from the other responses.

As the subgroup of participants who had opted for PCH was relatively small, our investigation did not include a direct comparison between the views of participants who were considering general practice to those who had chosen another specialty. By involving this aspect, future studies could provide additional insights regarding motivational or deterring factors in relation to PHC.

The study did not assess whether the COVID-19 pandemic had an impact on the perceptions and career intentions of medical students and residents towards general practice. It is likely that the pandemic which posed manyfold challenges and risks to GPs [5] influenced some answers and opinions of the study participants regarding general practice. It would be an interesting subject for future studies to investigate if and how the COVID-19 pandemic affected the determination of students and residents to choose or to reject general practice as a career option.

## Conclusion

Interest in becoming a GP was low among both students and residents. The study identified two main target areas to improve the attractiveness of general practice:

i. High-quality education throughout medical school with practical experience and knowledge of the profession’s specific content and skills. Negative perceptions of PHC could be addressed by intensified teaching and by promoting respectful attitudes towards primary care in medical schools.
ii. Ensuring that general practice is perceived as a profession with a promising future, offering independence and work-family balance. Measures by health policy makers should therefore focus on job security, stable income, professional autonomy, increasing employment options particularly for young GPs, and promoting job-family reconciliation. Moreover, promoting group offices and teamwork could increase the appeal of general practice for young physicians.

While introducing a specialist qualification for GPs in Austria could make general practice more attractive, it may not be the most crucial factor in increasing the number of GPs. A consistent academic representation of general practice in medical schools seems to be essential for improving scientific reputation.

The high willingness to work in rural areas among the present cohort, especially residents, is promising, and may encourage health policymakers to invest in endorsement measures for rural general practice.

### Electronic supplementary material

Below is the link to the electronic supplementary material.

Supplementary Material 1

Supplementary Material 2

Supplementary Material 3

## Data Availability

The datasets used during the current study are available from the corresponding author upon request.

## Abbreviations

CI: Confidence interval
GP: General practitioner
OR: Odds ratio
PHC: Primary healthcare
