# Supplementary material for: Attitudes towards career choice and general practice: a cross-sectional survey of medical students and residents in Tyrol, Austria
Source: BMC Med Educ. 2024 Mar 15;24:294. doi: 10.1186/s12909-024-05205-8 (PMC10943776; doi:10.1186/s12909-024-05205-8)
Supplement: Supplementary file 1 — Supplementary Material 1 [file 12909_2024_5205_MOESM1_ESM.docx]

### **Supplementary Table S1a**: Aspired values in the professional life and corresponding perceptions of general practice – MEDICAL STUDENTS and RESIDENTS

|  | **Yes, surely** | **Rather yes** | **Neutral** | **Rather no** | **Not at all** | ***n*** | **p-value** |
| --- | --- | --- | --- | --- | --- | --- | --- |
| **It is important to me to have a stable working position.** | | | | | | | |
| Students | 388 (73.6%) | 118 (22.4%) | 18 (3.4%) | 3 (0.6%) | 0 (0.0%) | *528* | 0.843 ^ii^ |
| Residents | 73 (70.9%) | 24 (23.3%) | 5 (4.9%) | 1 (1.0%) | 0 (0.0%) | *103* |  |
| **General practice offers a stable working position.** | | | | | | | |
| Students | 329 (62.3%) | 162 (30.7%) | 31 (5.9%) | 6 (1.1%) | 0 (0.0%) | *528* | 0.199 ^ii^ |
| Residents | 55 (53.4%) | 41 (39.8%) | 7 (6.8%) | 0 (0.0%) | 0 (0.0%) | *103* |  |
| **It is important to me to work in a profession with a promising future.** | | | | | | | |
| Students | 428 (81.5%) | 92 (17.5%) | 3 (0.6%) | 2 (0.4%) | 0 (0.0%) | *528* | **0.018** ^ii^ |
| Residents | 77 (74.8%) | 22 (21.4%) | 4 (3.9%) | 0 (0.0%) | 0 (0.0%) | *103* |  |
| **General practice is a profession with a promising future.** | | | | | | | |
| Students | 331 (62.9%) | 130 (24.7%) | 51 (9.7%) | 13 (2.5%) | 1 (0.2%) | *526* | 0.364 ^ii^ |
| Residents | 54 (52.4%) | 33 (32.0%) | 13 (12.6%) | 3 (2.9%) | 0 (0.0%) | *103* |  |
| **It is important to me to can organise my working times flexibly.** | | | | | | | |
| Students | 130 (24.7%) | 183 (34.7%) | 174 (33.0%) | 38 (7.2%) | 2 (0.4%) | *527* | 0.169 ^ii^ |
| Residents | 36 (35.0%) | 35 (34.0%) | 25 (24.3%) | 6 (5.8%) | 1 (1.0%) | *103* |  |
| **General practice offers the possibility to organise the working times flexibly.** | | | | | | | |
| Students | 107 (20.3%) | 179 (34.0%) | 151 (28.7%) | 79 (15.0%) | 11 (2.1%) | *527* | **0.006** ^ii^ |
| Residents | 11 (10.7%) | 25 (24.3%) | 40 (38.8%) | 22 (21.4%) | 5 (4.9%) | *103* |  |
| **It is important to me to have regular working times.** | | | | | | | |
| Students | 105 (20.1%) | 202 (38.7%) | 163 (31.2%) | 48 (9.2%) | 4 (0.8%) | *522* | **0.041** ^ii^ |
| Residents | 29 (28.4%) | 46 (45.1%) | 17 (16.7%) | 9 (8.8%) | 1 (1.0%) | *102* |  |
| **General practice offers the possibility to organise the working times on a regular basis.** | | | | | | | |
| Students | 129 (24.4%) | 177 (33.5%) | 142 (26.9%) | 67 (12.7%) | 13 (2.5%) | *528* | **<0.001** ^ii^ |
| Residents | 7 (6.8%) | 34 (33.0%) | 31 (30.1%) | 26 (25.2%) | 5 (4.9%) | *103* |  |
| **It is important to me to have a diversified daily routine.** | | | | | | | |
| Students | 278 (53.0%) | 181 (34.5%) | 58 (11.0%) | 8 (1.5%) | 0 (0.0%) | *525* | **0.047** ^ii^ |
| Residents | 43 (41.7%) | 37 (35.9%) | 20 (19.4%) | 3 (2.9%) | 0 (0.0%) | *103* |  |
| **General practice offers a diversified daily routine.** | | | | | | | |
| Students | 136 (25.8%) | 155 (29.4%) | 157 (29.7%) | 70 (13.3%) | 10 (1.9%) | *528* | **0.003** ^ii^ |
| Residents | 32 (31.1%) | 46 (44.7%) | 15 (14.6%) | 9 (8.7%) | 1 (1.0%) | *103* |  |
| **It is important to me to have few night duties.** | | | | | | | |
| Students | 89 (16.9%) | 120 (22.8%) | 174 (33.1%) | 112 (21.3%) | 31 (5.9%) | *526* | **0.047** ^ii^ |
| Residents | 28 (27.7%) | 23 (22.8%) | 34 (33.7%) | 13 (12.9%) | 3 (3.0%) | *101* |  |
| **In general practice one has few night duties.** | | | | | | | |
| Students | 285 (54.0%) | 158 (29.9%) | 64 (12.1%) | 20 (3.8%) | 1 (0.2%) | *528* | **<0.001** ^ii^ |
| Residents | 31 (30.1%) | 32 (31.1%) | 31 (30.1%) | 9 (8.7%) | 0 (0.0%) | *103* |  |
| **It is important to me to have good opportunities of income.** | | | | | | | |
| Students | 215 (41.0%) | 236 (45.0%) | 58 (11.1%) | 12 (2.3%) | 3 (0.6%) | *524* | 0.539 ^ii^ |
| Residents | 44 (42.7%) | 42 (40.8%) | 12 (11.7%) | 5 (4.9%) | 0 (0.0%) | *103* |  |
| **General practice offers good opportunities of income.** | | | | | | | |
| Students | 94 (17.8%) | 140 (26.5%) | 177 (33.5%) | 93 (17.6%) | 24 (4.5%) | *528* | 0.346 ^ii^ |
| Residents | 12 (11.8%) | 29 (28.4%) | 40 (39.2%) | 14 (13.7%) | 7 (6.9%) | *102* |  |
| **It is important to me to have an assured income.** | | | | | | | |
| Students | 384 (73.6%) | 120 (23.0%) | 15 (2.9%) | 3 (0.6%) | 0 (0.0%) | *522* | 0.610 ^ii^ |
| Residents | 70 (68.6%) | 29 (28.4%) | 2 (2.0%) | 1 (1.0%) | 0 (0.0%) | *102* |  |
| **In general practice one has an assured income.** | | | | | | | |
| Students | 187 (35.6%) | 213 (40.5%) | 102 (19.4%) | 24 (4.6%) | 0 (0.0%) | *526* | **0.022** ^ii^ |
| Residents | 23 (22.5%) | 48 (47.1%) | 24 (23.5%) | 6 (5.9%) | 1 (1.0%) | *102* |  |
| **It is important to me to be able to participate in research projects.** | | | | | | | |
| Students | 58 (11.1%) | 109 (20.8%) | 143 (27.3%) | 144 (27.5%) | 70 (13.4%) | *524* | **0.001** ^ii^ |
| Residents | 5 (4.9%) | 8 (7.8%) | 32 (31.4%) | 31 (30.4%) | 26 (25.5%) | *102* |  |
| **General practice offers the opportunity to participate in research projects.** | | | | | | | |
| Students | 15 (2.9%) | 60 (11.4%) | 157 (29.9%) | 233 (44.4%) | 60 (11.4%) | *525* | **0.049** ^ii^ |
| Residents | 4 (3.9%) | 5 (4.9%) | 23 (22.5%) | 51 (50.0%) | 19 (18.6%) | *102* |  |
| **It is important to me to deal with a variety of conditions.** | | | | | | | |
| Students | 212 (40.3%) | 215 (40.9%) | 86 (16.3%) | 10 (1.9%) | 3 (0.6%) | *526* | 0.175 ^ii^ |
| Residents | 45 (44.1%) | 41 (40.2%) | 10 (9.8%) | 4 (3.9%) | 2 (2.0%) | *102* |  |
| **General practice offers the opportunity to deal with a variety of conditions.** | | | | | | | |
| Students | 277 (52.5%) | 173 (32.8%) | 57 (10.8%) | 19 (3.6%) | 2 (0.2%) | *528* | 0.575 ^ii^ |
| Residents | 58 (56.3%) | 35 (34.0%) | 9 (8.7%) | 1 (1.0%) | 0 (0.0%) | *103* |  |
| **It is important to me to work according to the currently available scientific state of the art.** | | | | | | | |
| Students | 319 (60.6%) | 163 (31.0%) | 35 (6.7%) | 6 (1.1%) | 3 (0.6%) | *526* | **0.024** ^ii^ |
| Residents | 44 (43.1%) | 47 (46.1%) | 8 (7.8%) | 2 (2.0%) | 1 (1.0%) | *102* |  |
| **In general practice one works according to the currently available scientific state of the art.** | | | | | | | |
| Students | 61 (11.6%) | 137 (25.9%) | 210 (39.8%) | 100 (18.9%) | 20 (3.8%) | *528* | 0.228 ^ii^ |
| Residents | 5 (4.9%) | 23 (22.3%) | 48 (46.6%) | 23 (22.3%) | 4 (3.9%) | *103* |  |
| **It is important to me to conduct preventive activities.** | | | | | | | |
| Students | 247 (47.2%) | 141 (27.0%) | 85 (16.3%) | 46 (8.8%) | 4 (0.8%) | *523* | **0.036** ^ii^ |
| Residents | 42 (41.2%) | 41 (40.2%) | 13 (12.7%) | 4 (3.9%) | 2 (2.0%) | *102* |  |
| **General practice offers the possibility to conduct preventive activities.** | | | | | | | |
| Students | 431 (81.9%) | 83 (15.8%) | 9 (1.7%) | 3 (0.6%) | 0 (0.0%) | *526* | 0.129 ^ii^ |
| Residents | 75 (73.5%) | 23 (22.5%) | 4 (3.9%) | 0 (0.0%) | 0 (0.0%) | *102* |  |
| **It is important to me to be exposed to a low physical burden in my professional life.** | | | | | | | |
| Students | 24 (4.6%) | 94 (17.8%) | 161 (30.6%) | 166 (31.5%) | 82 (15.6%) | *527* | 0.973 ^ii^ |
| Residents | 5 (4.9%) | 16 (15.7%) | 31 (30.4%) | 35 (34.3%) | 15 (14.7%) | *102* |  |
| **In general practice one is exposed to a low physical burden.** | | | | | | | |
| Students | 119 (22.6%) | 182 (34.5%) | 170 (32.3%) | 49 (9.3%) | 7 (1.3%) | *527* | **<0.001** ^ii^ |
| Residents | 8 (7.8%) | 22 (21.6%) | 49 (48.0%) | 19 (18.6%) | 4 (3.9%) | *102* |  |
| **It is important to me to be exposed to a low psychological burden in my professional life.** | | | | | | | |
| Students | 52 (9.9%) | 120 (22.9%) | 179 (34.2%) | 137 (26.1%) | 36 (6.9%) | *524* | 0.693 ^ii^ |
| Residents | 13 (12.6%) | 25 (24.3%) | 28 (27.2%) | 30 (29.1%) | 7 (6.8%) | *103* |  |
| **In general practice one is exposed to a low psychological burden.** | | | | | | | |
| Students | 26 (4.9%) | 66 (12.5%) | 211 (40.0%) | 190 (36.1%) | 34 (6.5%) | *527* | **0.001** ^ii^ |
| Residents | 0 (0.0%) | 6 (5.8%) | 35 (34.0%) | 47 (45.6%) | 15 (14.6%) | *103* |  |
| **It is important to me to have long-term relationships with the patients.** | | | | | | | |
| Students | 74 (14.1%) | 138 (26.2%) | 186 (35.4%) | 103 (19.6%) | 25 (4.8%) | *526* | 0.771 ^ii^ |
| Residents | 13 (12.7%) | 23 (22.5%) | 43 (42.2%) | 19 (18.6%) | 4 (3.9%) | *102* |  |
| **In general practice one has long-term relationships with patients.** | | | | | | | |
| Students | 449 (85.0%) | 65 (12.3%) | 12 (2.3%) | 1 (0.2%) | 1 (0.2%) | *528* | **0.008** ^ii^ |
| Residents | 77 (74.8%) | 23 (22.3%) | 1 (1.0%) | 2 (1.9%) | 0 (0.0%) | *103* |  |
| **It is important to me to conduct emergency medicine activities.** | | | | | | | |
| Students | 142 (27.0%) | 138 (26.2%) | 115 (21.9%) | 98 (18.6%) | 33 (6.3%) | *526* | 0.665 ^ii^ |
| Residents | 34 (33.3%) | 27 (26.5%) | 17 (16.7%) | 18 (17.6%) | 6 (5.9%) | *102* |  |
| **General practice offers the possibility to conduct emergency medicine activities.** | | | | | | | |
| Students | 75 (14.3%) | 143 (27.2%) | 207 (39.4%) | 84 (16.0%) | 17 (3.2%) | *526* | 0.459 ^ii^ |
| Residents | 13 (12.6%) | 32 (31.1%) | 45 (43.7%) | 12 (11.7%) | 1 (1.0%) | *103* |  |

^ii^ Chi² Test

### **Supplementary Table S1b**: Aspired values in the professional life and corresponding perceptions of general practice – MEDICAL STUDENTS and RESIDENTS

|  | **Yes, surely** | **Rather yes** | **Neutral** | **Rather no** | **Not at all** | ***n*** | **p-value** |
| --- | --- | --- | --- | --- | --- | --- | --- |
| **It is important to me to apply a broad medical knowledge.** | | | | | | | |
| Students | 250 (47.9%) | 182 (34.9%) | 66 (12.6%) | 23 (4.4%) | 1 (0.2%) | *522* | 0.124 ^ii^ |
| Residents | 59 (57.8%) | 33 (32.4%) | 7 (6.9%) | 2 (2.0%) | 1 (1.0%) | *102* |  |
| **General practice offers the possibility to apply a broad medical knowledge.** | | | | | | | |
| Students | 302 (57.2%) | 173 (32.8%) | 42 (8.0%) | 9 (1.7%) | 2 (0.4%) | *528* | 0.186 ^ii^ |
| Residents | 47 (45.6%) | 43 (41.7%) | 12 (11.7%) | 1 (1.0%) | 0 (0.0%) | *103* |  |
| **It is important to me to have much time for recreation.** | | | | | | | |
| Students | 150 (28.5%) | 206 (39.2%) | 135 (25.7%) | 31 (5.9%) | 4 (0.8%) | *526* | 0.077 ^ii^ |
| Residents | 43 (41.7%) | 37 (35.9%) | 19 (18.4%) | 3 (2.9%) | 1 (1.0%) | *103* |  |
| **In general practice one has much time for recreation.** | | | | | | | |
| Students | 70 (13.3%) | 133 (25.3%) | 229 (43.6%) | 76 (14.5%) | 17 (3.2%) | *525* | **<0.001** ^ii^ |
| Residents | 1 (1.0%) | 11 (10.7%) | 50 (48.5%) | 32 (31.1%) | 9 (8.7%) | *103* |  |
| **It is important to me to be able to separate the professional life from the private life.** | | | | | | | |
| Students | 192 (36.4%) | 197 (37.4%) | 97 (18.4%) | 39 (7.4%) | 2 (0.4%) | *527* | 0.422 ^ii^ |
| Residents | 44 (42.7%) | 41 (39.8%) | 13 (12.6%) | 5 (4.9%) | 0 (0.0%) | *103* |  |
| **General practice offers the possibility to separate the professional life from the private life.** | | | | | | | |
| Students | 55 (10.5%) | 120 (22.9%) | 173 (33.0%) | 148 (28.2%) | 29 (5.5%) | *525* | **<0.001** ^ii^ |
| Residents | 1 (1.0%) | 13 (12.6%) | 45 (43.7%) | 31 (30.1%) | 13 (12.6%) | *103* |  |
| **It is important to me to have the opportunity of working on a part-time basis.** | | | | | | | |
| Students | 153 (29.1%) | 146 (27.8%) | 104 (19.8%) | 83 (15.8%) | 40 (7.6%) | *526* | 0.208 ^ii^ |
| Residents | 39 (37.9%) | 31 (30.1%) | 14 (13.6%) | 15 (14.6%) | 4 (3.9%) | *103* |  |
| **General practice offers the opportunity to work on a part-time basis.** | | | | | | | |
| Students | 142 (26.9%) | 186 (35.2%) | 122 (23.1%) | 66 (12.5%) | 12 (2.3%) | *528* | **0.007** ^ii^ |
| Residents | 11 (10.8%) | 49 (48.0%) | 23 (22.5%) | 15 (14.7%) | 4 (3.9%) | *102* |  |
| **It is important to me to reconcile family and professional life.** | | | | | | | |
| Students | 350 (66.7%) | 122 (23.2%) | 31 (5.9%) | 16 (3.0%) | 6 (1.1%) | *525* | 0.990 ^ii^ |
| Residents | 71 (68.9%) | 23 (22.3%) | 5 (4.9%) | 3 (2.9%) | 1 (1.0%) | *103* |  |
| **General practice offers the possibility to reconcile family and professional life.** | | | | | | | |
| Students | 173 (32.8%) | 228 (43.3%) | 103 (19.5%) | 18 (3.4%) | 5 (0.9%) | *527* | **<0.001** ^ii^ |
| Residents | 10 (9.8%) | 49 (48.0%) | 33 (32.4%) | 9 (8.8%) | 1 (1.0%) | *102* |  |
| **It is important to me that my profession earns public recognition.** | | | | | | | |
| Students | 86 (16.3%) | 164 (31.1%) | 118 (22.4%) | 111 (21.1%) | 48 (9.1%) | *527* | **0.013** ^ii^ |
| Residents | 6 (5.8%) | 34 (33.0%) | 36 (35.0%) | 19 (18.4%) | 8 (7.8%) | *103* |  |
| **General practice is a profession which earns public recognition.** | | | | | | | |
| Students | 115 (21.9%) | 205 (39.0%) | 132 (25.1%) | 58 (11.0%) | 16 (3.0%) | *526* | **0.032** ^ii^ |
| Residents | 14 (13.6%) | 35 (34.0%) | 34 (33.0%) | 12 (11.7%) | 8 (7.8%) | *103* |  |
| **It is important to me to be able to realise professional aims.** | | | | | | | |
| Students | 285 (54.2%) | 184 (35.0%) | 48 (9.1%) | 6 (1.1%) | 3 (0.6%) | *526* | **0.027** ^ii^ |
| Residents | 39 (38.2%) | 46 (45.1%) | 14 (13.7%) | 3 (2.9%) | 0 (0.0%) | *102* |  |
| **General practice offers the possibility to realise professional aims.** | | | | | | | |
| Students | 80 (15.2%) | 163 (30.9%) | 181 (34.3%) | 93 (17.6%) | 10 (1.9%) | *527* | **0.023** ^ii^ |
| Residents | 10 (9.7%) | 43 (41.7%) | 35 (34.0%) | 10 (9.7%) | 5 (4.9%) | *103* |  |
| **It is important to me to realise private aims.** | | | | | | | |
| Students | 340 (64.8%) | 160 (30.5%) | 19 (3.6%) | 5 (1.0%) | 1 (0.2%) | *525* | 0.317 ^ii^ |
| Residents | 64 (62.1%) | 31 (30.1%) | 8 (7.8%) | 0 (0.0%) | 0 (0.0%) | *103* |  |
| **General practice offers the possibility to realise private aims.** | | | | | | | |
| Students | 153 (29.0%) | 252 (47.8%) | 102 (19.4%) | 16 (3.0%) | 4 (0.8%) | *527* | **<0.001** ^ii^ |
| Residents | 7 (6.8%) | 40 (38.8%) | 45 (43.7%) | 8 (7.8%) | 3 (2.9%) | *103* |  |
| **It is important to me that my specialty has a positive perception in the medical community.** | | | | | | | |
| Students | 147 (27.9%) | 161 (30.6%) | 104 (19.8%) | 82 (15.6%) | 32 (6.1%) | *526* | 0.185 ^ii^ |
| Residents | 23 (22.5%) | 39 (38.2%) | 20 (19.6%) | 10 (9.8%) | 10 (9.8%) | *102* |  |
| **General practice enjoys a positive perception in the medical community.** | | | | | | | |
| Students | 38 (7.3%) | 96 (18.3%) | 207 (39.5%) | 139 (26.5%) | 44 (8.4%) | *524* | **0.012** ^ii^ |
| Residents | 4 (3.9%) | 15 (14.6%) | 29 (28.2%) | 42 (40.8%) | 13 (12.6%) | *103* |  |
| **It is important to me that my specialty is positively presented in the media.** | | | | | | | |
| Students | 91 (17.3%) | 142 (27.0%) | 132 (25.1%) | 93 (17.7%) | 67 (12.8%) | *525* | 0.607 ^ii^ |
| Residents | 16 (15.5%) | 36 (35.0%) | 23 (22.3%) | 17 (16.5%) | 11 (10.7%) | *103* |  |
| **General practice is positively presented in the media.** | | | | | | | |
| Students | 61 (11.6%) | 185 (35.2%) | 188 (35.7%) | 73 (13.9%) | 19 (3.6%) | *526* | **0.015** ^ii^ |
| Residents | 4 (3.9%) | 28 (27.2%) | 46 (44.7%) | 17 (16.5%) | 8 (7.8%) | *103* |  |
| **It is important to me that my specialty is appreciated by the patients.** | | | | | | | |
| Students | 222 (42.2%) | 195 (37.1%) | 71 (13.5%) | 27 (5.1%) | 11 (2.1%) | *526* | 0.304 ^ii^ |
| Residents | 38 (36.9%) | 37 (35.9%) | 14 (13.6%) | 10 (9.7%) | 4 (3.9%) | *103* |  |
| **General practice is appreciated by the patients.** | | | | | | | |
| Students | 174 (33.1%) | 163 (31.0%) | 131 (25.0%) | 42 (8.0%) | 15 (2.9%) | *525* | 0.383 ^ii^ |
| Residents | 25 (24.5%) | 32 (31.4%) | 31 (30.4%) | 9 (8.8%) | 5 (4.9%) | *102* |  |
| **It is important to me that my specialty is appreciated by the political decision makers.** | | | | | | | |
| Students | 174 (33.1%) | 169 (32.1%) | 89 (16.9%) | 47 (8.9%) | 47 (8.9%) | *526* | 0.514 ^ii^ |
| Residents | 40 (38.8%) | 32 (31.1%) | 11 (10.7%) | 11 (10.7%) | 9 (8.7%) | *103* |  |
| **General practice is appreciated by the political decision makers.** | | | | | | | |
| Students | 40 (7.6%) | 104 (19.8%) | 168 (32.0%) | 135 (25.7%) | 78 (14.9%) | *525* | 0.088 ^ii^ |
| Residents | 3 (3.0%) | 12 (11.9%) | 37 (36.6%) | 28 (27.7%) | 21 (20.8%) | *101* |  |
| **It is important to me that my specialty has a high significance/status during medical education and specialisation.** | | | | | | | |
| Students | 173 (33.0%) | 173 (33.0%) | 118 (22.5%) | 45 (8.6%) | 16 (3.0%) | *525* | 0.405 ^ii^ |
| Residents | 37 (35.9%) | 41 (39.8%) | 17 (16.5%) | 6 (5.8%) | 2 (1.9%) | *103* |  |
| **General practice has a high significance/status during medical education and specialisation.** | | | | | | | |
| Students | 23 (4.4%) | 89 (16.9%) | 185 (35.2%) | 173 (32.9%) | 56 (10.6%) | *526* | **0.010** ^ii^ |
| Residents | 2 (2.0%) | 8 (7.8%) | 31 (30.4%) | 41 (40.2%) | 20 (19.6%) | *102* |  |
| **In general practice one makes independent, self-reliant decisions.** | | | | | | | |
| Students | 338 (64.0%) | 137 (25.9%) | 41 (7.8%) | 12 (2.3%) | 0 (0.0%) | *528* | 0.374 ^ii^ |
| Residents | 74 (71.8%) | 23 (22.3%) | 4 (3.9%) | 2 (1.9%) | 0 (0.0%) | *103* |  |
| **In general practice one has much contact with patients.** | | | | | | | |
| Students | 447 (85.0%) | 70 (13.3%) | 6 (1.1%) | 3 (0.6%) | 0 (0.0%) | *526* | **0.040** ^ii^ |
| Residents | 77 (74.8%) | 23 (22.3%) | 3 (2.9%) | 0 (0.0%) | 0 (0.0%) | *103* |  |
| **In general practice one works in a team.** | | | | | | | |
| Students | 91 (17.2%) | 103 (19.5%) | 162 (30.7%) | 134 (25.4%) | 38 (7.2%) | *528* | 0.799 ^ii^ |
| Residents | 13 (12.6%) | 20 (19.4%) | 36 (35.0%) | 26 (25.2%) | 8 (7.8%) | *103* |  |
| **General practice is a relevant subject during the whole period of medical school.** | | | | | | | |
| Students | 220 (41.7%) | 149 (28.3%) | 79 (15.0%) | 58 (11.0%) | 21 (4.0%) | *527* | 0.613 ^ii^ |
| Residents | 36 (35.0%) | 30 (29.1%) | 16 (15.5%) | 16 (15.5%) | 5 (4.9%) | *103* |  |
| **General practice is a relevant subject for the future professional activity.** | | | | | | | |
| Students | 209 (39.7%) | 181 (34.3%) | 83 (15.7%) | 37 (7.0%) | 17 (3.2%) | *527* | 0.913 ^ii^ |
| Residents | 43 (41.7%) | 31 (30.1%) | 19 (18.4%) | 7 (6.8%) | 3 (2.9%) | *103* |  |
| **I deem the profession of a GP as attractive.** | | | | | | | |
| Students | 85 (16.1%) | 120 (22.7%) | 168 (31.8%) | 92 (17.4%) | 63 (11.9%) | *528* | 0.415 ^ii^ |
| Residents | 15 (14.6%) | 30 (29.1%) | 31 (30.1%) | 20 (19.4%) | 7 (6.8%) | *103* |  |

^ii^ Chi² Test
