# Supplementary material for: Attitudes towards career choice and general practice: a cross-sectional survey of medical students and residents in Tyrol, Austria
Source: BMC Med Educ. 2024 Mar 15;24:294. doi: 10.1186/s12909-024-05205-8 (PMC10943776; doi:10.1186/s12909-024-05205-8)
Supplement: Supplementary file 2 — Supplementary Material 2 [file 12909_2024_5205_MOESM2_ESM.docx]

### **Supplementary Table S2a**: Gender-related significant^§^ differences of individual importance of aspects in the professional life (part A of the questionnaire) and possibilities in general practice (part B) – MEDICAL STUDENTS

| **MEDICAL STUDENTS** | **Yes, surely** | **Rather yes** | **Neutral** | **Rather no** | **Not at all** | ***n*** | **p-value** |
| --- | --- | --- | --- | --- | --- | --- | --- |
| **A1. It is important to me to have a stable working position.** | | | | | | |  |
| Females | 234 (79.1%) | 51 (17.2%) | 10 (3.4%) | 1 (0.3%) | 0 (0.0%) | *296* | **0.008** ^ii^ |
| Males | 152 (66.4%) | 67 (29.3%) | 8 (3.5%) | 2 (0.9%) | 0 (0.0%) | *229* |  |
| **B1. General practice offers a stable working position.** | | | | | | |  |
| Females | 177 (59.8%) | 99 (33.4%) | 17 (5.7%) | 3 (1.0%) | 0 (0.0%) | *296* | 0.517 ^ii^ |
| Males | 150 (65.2%) | 63 (27.4%) | 14 (6.1%) | 3 (1.3%) | 0 (0.0%) | *230* |  |
| **A3. It is important to me to can organize my working times flexibly.** | | | | | | | |
| Females | 83 (28.0%) | 97 (32.8%) | 99 (33.4%) | 15 (5.1%) | 2 (0.7%) | *296* | **0.046** ^ii^ |
| Males | 47 (20.5%) | 85 (37.1%) | 74 (32.3%) | 23 (10.0%) | 0 (0.0%) | *229* |  |
| **B3. General practice offers the possibility to organize the working times flexibly.** | | | | | | | |
| Females | 69 (23.4%) | 99 (33.6%) | 87 (29.5%) | 33 (11.2%) | 7 (2.4%) | *295* | **0.038** ^ii^ |
| Males | 38 (16.5%) | 79 (34.3%) | 63 (27.4%) | 46 (20.0%) | 4 (1.7%) | *230* |  |
| **A4. It is important to me to have regular working times.** | | | | | | | |
| Females | 67 (23.0%) | 113 (38.8%) | 92 (31.6%) | 17 (5.8%) | 2 (0.7%) | *291* | **0.027** ^ii^ |
| Males | 38 (16.6%) | 88 (38.4%) | 70 (30.6%) | 31 (13.5%) | 2 (0.9%) | *229* |  |
| **B4. General practice offers the possibility to organize the working times on a regular basis.** | | | | | | | |
| Females | 88 (29.7%) | 95 (32.1%) | 75 (25.3%) | 33 (11.1%) | 5 (1.7%) | *296* | **0.022** ^ii^ |
| Males | 41 (17.8%) | 80 (34.8%) | 67 (29.1%) | 34 (14.8%) | 8 (3.5%) | *230* |  |
| **A6. It is important to me to have few night duties.** | | | | | | | |
| Females | 59 (19.9%) | 79 (26.7%) | 98 (33.1%) | 50 (16.9%) | 10 (3.4%) | *296* | **<0.001** ^ii^ |
| Males | 29 (12.7%) | 41 (18.0%) | 75 (32.9%) | 62 (27.2%) | 21 (9.2%) | *228* |  |
| **B7. In general practice one has few night duties.** | | | | | | | |
| Females | 183 (61.8%) | 85 (28.7%) | 21 (7.1%) | 7 (2.4%) | 0 (0.0%) | *296* | **<0.001** ^ii^ |
| Males | 100 (43.5%) | 73 (31.7%) | 43 (18.7%) | 13 (5.7%) | 1 (0.4%) | *230* |  |
| **A8. It is important to me to have an assured income.** | | | | | | | |
| Females | 228 (77.8%) | 59 (20.1%) | 5 (1.7%) | 1 (0.3%) | 0 (0.0%) | *293* | **0.044** ^ii^ |
| Males | 154 (67.8%) | 61 (26.9%) | 10 (4.4%) | 2 (0.9%) | 0 (0.0%) | *227* |  |
| **B9. In general practice one has an assured income.** | | | | | | | |
| Females | 101 (34.2%) | 123 (41.7%) | 61 (20.7%) | 10 (3.4%) | 0 (0.0%) | *295* | 0.392 ^ii^ |
| Males | 84 (36.7%) | 90 (39.3%) | 41 (17.9%) | 14 (6.1%) | 0 (0.0%) | *229* |  |
| **A11. It is important to me to work according to the currently available scientific state of the art.** | | | | | | | |
| Females | 175 (59.3%) | 95 (32.2%) | 22 (7.5%) | 2 (0.7%) | 1 (0.3%) | *295* | 0.540 ^ii^ |
| Males | 143 (62.4%) | 67 (29.3%) | 13 (5.7%) | 4 (1.7%) | 2 (0.9%) | *229* |  |
| **B12. In general practice one works according to the currently available scientific state of the art.** | | | | | | | |
| Females | 41 (13.9%) | 74 (25.0%) | 122 (41.2%) | 53 (17.9%) | 6 (2.0%) | *296* | **0.039** ^ii^ |
| Males | 19 (8.3%) | 62 (27.0%) | 88 (38.3%) | 47 (20.4%) | 14 (6.1%) | *230* |  |
| **A13. It is important to me to be exposed to a low physical burden in my professional life.** | | | | | | | |
| Females | 17 (5.7%) | 65 (22.0%) | 97 (32.8%) | 81 (27.4%) | 36 (12.2%) | *296* | **0.001** ^ii^ |
| Males | 6 (2.6%) | 29 (12.7%) | 63 (27.5%) | 85 (37.1%) | 46 (20.1%) | *229* |  |
| **B14. In general practice one is exposed to a low physical burden.** | | | | | | | |
| Females | 82 (27.8%) | 107 (36.3%) | 85 (28.8%) | 19 (6.4%) | 2 (0.7%) | *295* | **0.001** ^ii^ |
| Males | 36 (15.7%) | 74 (32.2%) | 85 (37.0%) | 30 (13.0%) | 5 (2.2%) | *230* |  |
| **A14. It is important to me to be exposed to a low psychological burden in my professional life.** | | | | | | | |
| Females | 30 (10.2%) | 83 (28.1%) | 107 (36.3%) | 61 (20.7%) | 14 (4.7%) | *295* | <**0.001** ^ii^ |
| Males | 21 (9.3%) | 37 (16.3%) | 71 (31.3%) | 76 (33.5%) | 22 (9.7%) | *227* |  |
| **B15. In general practice one is exposed to a low psychological burden.** | | | | | | | |
| Females | 15 (5.1%) | 42 (14.2%) | 121 (41.0%) | 102 (34.6%) | 15 (5.1%) | *295* | 0.366 ^ii^ |
| Males | 10 (4.3%) | 24 (10.4%) | 89 (38.7%) | 88 (38.3%) | 19 (8.3%) | *230* |  |
| **A20. It is important to me to have the opportunity of working on a part-time basis.** | | | | | | | |
| Females | 118 (40.0%) | 90 (30.5%) | 58 (19.7%) | 19 (6.4%) | 10 (3.4%) | *295* | <**0.001** ^ii^ |
| Males | 34 (14.8%) | 55 (24.0%) | 46 (20.1%) | 64 (27.9%) | 30 (13.1%) | *229* |  |
| **B23. General practice offers the opportunity to work on a part-time basis.** | | | | | | | |
| Females | 89 (30.1%) | 104 (35.1%) | 68 (23.0%) | 29 (9.8%) | 6 (2.0%) | *296* | 0.137 ^ii^ |
| Males | 52 (22.6%) | 81 (35.2%) | 54 (23.5%) | 37 (16.1%) | 6 (2.6%) | *230* |  |
| **A21. It is important to me to reconcile family and professional life.** | | | | | | | |
| Females | 223 (75.3%) | 44 (14.9%) | 17 (5.7%) | 9 (3.0%) | 3 (1.0%) | *296* | <**0.001** ^ii^ |
| Males | 127 (55.7%) | 77 (33.8%) | 14 (6.1%) | 7 (3.1%) | 3 (1.3%) | *228* |  |
| **B24. General practice offers the possibility to reconcile family and professional life.** | | | | | | | |
| Females | 109 (36.8%) | 125 (42.2%) | 52 (17.6%) | 7 (2.4%) | 3 (1.0%) | *296* | 0.121 ^ii^ |
| Males | 63 (27.5%) | 102 (44.5%) | 51 (22.3%) | 11 (4.8%) | 2 (0.9%) | *229* |  |
| **B16. In general practice one has much contact with patients.** | | | | | | | |
| Females | 262 (89.1%) | 27 (9.2%) | 3 (1.0%) | 2 (0.7%) | 0 (0.0%) | *294* | **0.016** ^ii^ |
| Males | 183 (79.6%) | 43 (18.7%) | 3 (1.3%) | 1 (0.4%) | 0 (0.0%) | *230* |  |

^ii^ Chi² Test

^§^ To keep the length of the tables reasonable, only items with significant results are reported for this part of the questionnaire. As these items were paired (‘It is important to me…’ – ‘In general practice…’), for each item with a significant result the respectively corresponding item is also reported (regardless whether it was significant or not).

### **Supplementary Table S2b**: Gender-related significant^§^ differences of individual importance of aspects in the professional life (part A of the questionnaire) and possibilities in general practice (part B) – RESIDENTS

| **RESIDENTS** | **Yes, surely** | **Rather yes** | **Neutral** | **Rather no** | **Not at all** | ***n*** | **p-value** |
| --- | --- | --- | --- | --- | --- | --- | --- |
| **A6. It is important to me to have few night duties.** | | | | | | | |
| Females | 20 (33.9%) | 12 (20.3%) | 23 (39.0%) | 3 (5.1%) | 1 (1.7%) | *59* | **0.027** ^ii^ |
| Males | 8 (19.0%) | 11 (26.2%) | 11 (26.2%) | 10 (23.8%) | 2 (4.8%) | *42* |  |
| **B7. In general practice one has few night duties.** | | | | | | | |
| Females | 19 (32.2%) | 18 (30.5%) | 20 (33.9%) | 2 (3.4%) | 0 (0.0%) | *59* | 0.145 ^ii^ |
| Males | 12 (27.3%) | 14 (31.8%) | 11 (25.0%) | 7 (15.9%) | 0 (0.0%) | *44* |  |
| **A10. It is important to me to deal with a variety of conditions.** | | | | | | | |
| Females | 29 (50.0%) | 24 (41.4%) | 2 (3.4%) | 3 (5.2%) | 0 (0.0%) | *58* | **0.044** ^ii^ |
| Males | 16 (36.4%) | 17 (38.6%) | 8 (18.2%) | 1 (2.3%) | 2 (4.5%) | *44* |  |
| **B11. General practice offers the opportunity to deal with a variety of conditions.** | | | | | | | |
| Females | 35 (59.3%) | 21 (35.6%) | 2 (3.4%) | 1 (1.7%) | 0 (0.0%) | *59* | 0.133 ^ii^ |
| Males | 23 (52.3%) | 14 (31.8%) | 7 (15.9%) | 0 (0.0%) | 0 (0.0%) | *44* |  |
| **A13. It is important to me to be exposed to a low physical burden in my professional life.** | | | | | | | |
| Females | 2 (3.4%) | 13 (22.0%) | 19 (32.2%) | 17 (28.8%) | 8 (13.6%) | *59* | 0.218 ^ii^ |
| Males | 3 (7.0%) | 3 (7.0%) | 12 (27.9%) | 18 (41.9%) | 7 (16.3%) | *43* |  |
| **B14. In general practice one is exposed to a low physical burden.** | | | | | | | |
| Females | 5 (8.5%) | 18 (30.5%) | 23 (39.0%) | 9 (15.3%) | 4 (6.8%) | *59* | **0.022** ^ii^ |
| Males | 3 (7.0%) | 4 (9.3%) | 26 (60.5%) | 10 (23.3%) | 0 (0.0%) | *43* |  |
| **A17. It is important to me to apply a broad medical knowledge.** | | | | | | | |
| Females | 41 (70.7%) | 13 (22.4%) | 3 (5.2%) | 1 (1.7%) | 0 (0.0%) | *58* | **0.043** ^ii^ |
| Males | 18 (40.9%) | 20 (45.5%) | 4 (9.1%) | 1 (2.3%) | 1 (2.3%) | *44* |  |
| **B20. General practice offers the possibility to apply a broad medical knowledge.** | | | | | | | |
| Females | 33 (55.9%) | 18 (30.5%) | 8 (13.6%) | 0 (0.0%) | 0 (0.0%) | *59* | **0.027** ^ii^ |
| Males | 14 (31.8%) | 25 (56.8%) | 4 (9.1%) | 1 (2.3%) | 0 (0.0%) | *44* |  |
| **A20. It is important to me to have the opportunity of working on a part-time basis.** | | | | | | | |
| Females | 28 (47.5%) | 19 (32.2%) | 7 (11.9%) | 4 (6.8%) | 1 (1.7%) | *59* | **0.023 ^ii^** |
| Males | 11 (25.0%) | 12 (27.3%) | 7 (15.9%) | 11 (25.0%) | 3 (6.8%) | *44* |  |
| **B23. General practice offers the opportunity to work on a part-time basis.** | | | | | | | |
| Females | 9 (15.3%) | 28 (47.5%) | 11 (18.6%) | 10 (16.9%) | 1 (1.7%) | *59* | 0.215 ^ii^ |
| Males | 2 (4.7%) | 21 (48.8%) | 12 (27.9%) | 5 (11.6%) | 3 (7.0%) | *43* |  |

^ii^ Chi² Test

^§^ To keep the length of the tables reasonable, only items with significant results are reported for this part of the questionnaire. As these items were paired (‘It is important to me…’ – ‘In general practice…’), for each item with a significant result the respectively corresponding item is also reported (regardless whether it was significant or not).

### **Supplementary Table S3a**: Age-related significant^§^ differences of individual importance of aspects in the professional life (part A of the questionnaire) and possibilities in general practice (part B) – MEDICAL STUDENTS

| **MEDICAL STUDENTS** | **Yes, surely** | **Rather yes** | **Neutral** | **Rather no** | **Not at all** | ***n*** | **p-value** |
| --- | --- | --- | --- | --- | --- | --- | --- |
| **A3. It is important to me to can organize my working times flexibly.** | | | | | | | |
| < 23 years | 29 (17.6%) | 54 (32.7%) | 67 (40.6%) | 15 (9.1%) | 0 (0.0%) | *165* | **0.028** ^ii^ |
| ≥ 23 years | 96 (27.6%) | 124 (35.6%) | 104 (29.9%) | 22 (6.3%) | 2 (0.6%) | *348* |  |
| **B3. General practice offers the possibility to organize the working times flexibly.** | | | | | | | |
| < 23 years | 33 (20.0%) | 58 (35.2%) | 48 (29.1%) | 22 (13.1%) | 4 (2.4%) | *165* | 0.945 ^ii^ |
| ≥ 23 years | 72 (20.7%) | 115 (33.1%) | 98 (28.2%) | 55 (15.9%) | 7 (2.0%) | *347* |  |
| **A5. It is important to me to have a diversified daily routine.** | | | | | | | |
| < 23 years | 96 (58.5%) | 55 (33.5%) | 12 (7.3%) | 1 (0.6%) | 0 (0.0%) | *164* | 0.093 ^ii^ |
| ≥ 23 years | 174 (50.0%) | 121 (34.8%) | 46 (13.2%) | 7 (2.0%) | 0 (0.0%) | *348* |  |
| **B5. General practice offers a diversified daily routine.** | | | | | | | |
| < 23 years | 35 (21.1%) | 40 (24.2%) | 63 (38.2%) | 22 (13.3%) | 5 (3.0%) | *165* | **0.021** ^ii^ |
| ≥ 23 years | 97 (27.9%) | 112 (32.2%) | 90 (25.9%) | 44 (12.6%) | 5 (1.4%) | *348* |  |
| **A8. It is important to me to have an assured income.** | | | | | | | |
| < 23 years | 120 (73.2%) | 39 (23.8%) | 5 (3.0%) | 0 (0.0%) | 0 (0.0%) | *164* | 0.689 ^ii^ |
| ≥ 23 years | 252 (73.3%) | 79 (23.0%) | 10 (2.9%) | 3 (0.9%) | 0 (0.0%) | *344* |  |
| **B9. In general practice one has an assured income.** | | | | | | | |
| < 23 years | 74 (45.1%) | 60 (36.6%) | 27 (16.5%) | 3 (1.8%) | 0 (0.0%) | *164* | **0.004** ^ii^ |
| ≥ 23 years | 105 (30.3%) | 150 (43.2%) | 71 (20.5%) | 21 (6.1%) | 0 (0.0%) | *347* |  |
| **A10. It is important to me to deal with a variety of conditions.** | | | | | | | |
| < 23 years | 76 (46.1%) | 59 (35.8%) | 30 (18.2%) | 0 (0.0%) | 0 (0.0%) | *165* | **0.043** ^ii^ |
| ≥ 23 years | 133 (38.3%) | 151 (43.5%) | 51 (14.7%) | 9 (2.6%) | 3 (0.9%) | *347* |  |
| **B11. General practice offers the opportunity to deal with a variety of conditions.** | | | | | | | |
| < 23 years | 81 (49.1%) | 58 (35.2%) | 15 (9.1%) | 10 (6.1%) | 1 (0.6%) | *165* | 0.096 ^ii^ |
| ≥ 23 years | 191 (54.9%) | 107 (30.7%) | 41 (11.8%) | 9 (2.6%) | 0 (0.0%) | *348* |  |
| **A11. It is important to me to work according to the currently available scientific state of the art.** | | | | | | | |
| < 23 years | 105 (64.0%) | 45 (27.4%) | 12 (7.3%) | 2 (1.2%) | 0 (0.0%) | *164* | 0.643 ^ii^ |
| ≥ 23 years | 209 (60.1%) | 110 (31.6%) | 22 (6.3%) | 4 (1.1%) | 3 (0.9%) | *348* |  |
| **B12. In general practice one works according to the currently available scientific state of the art.** | | | | | | | |
| < 23 years | 23 (13.9%) | 53 (32.1%) | 52 (31.5%) | 32 (19.4%) | 5 (3.0%) | *165* | **0.043** ^ii^ |
| ≥ 23 years | 37 (10.6%) | 78 (22.4%) | 154 (44.3%) | 66 (19.0%) | 13 (3.7%) | *348* |  |
| **A13. It is important to me to be exposed to a low physical burden in my professional life.** | | | | | | | |
| < 23 years | 4 (2.4%) | 25 (15.2%) | 44 (26.7%) | 67 (40.6%) | 25 (15.2%) | *165* | **0.022** ^ii^ |
| ≥ 23 years | 20 (5.7%) | 68 (19.5%) | 111 (31.9%) | 94 (27.0%) | 55 (15.8%) | *348* |  |
| **B14. In general practice one is exposed to a low physical burden.** | | | | | | | |
| < 23 years | 46 (27.9%) | 55 (33.3%) | 48 (29.1%) | 16 (9.7%) | 0 (0.0%) | *165* | 0.116 ^ii^ |
| ≥ 23 years | 69 (19.9%) | 120 (34.6%) | 118 (34.0%) | 33 (9.5%) | 7 (2.0%) | *347* |  |
| **A14. It is important to me to be exposed to a low psychological burden in my professional life.** | | | | | | | |
| < 23 years | 9 (5.5%) | 35 (21.5%) | 54 (33.1%) | 55 (33.7%) | 10 (6.1%) | *163* | **0.025** ^ii^ |
| ≥ 23 years | 42 (12.1%) | 85 (24.5%) | 118 (34.0%) | 77 (22.2%) | 25 (7.2%) | *347* |  |
| **B15. In general practice one is exposed to a low psychological burden.** | | | | | | | |
| < 23 years | 12 (7.3%) | 25 (15.2%) | 71 (43.3%) | 49 (29.9%) | 7 (4.3%) | *164* | **0.039** ^ii^ |
| ≥ 23 years | 13 (3.7%) | 37 (10.6%) | 135 (38.8%) | 136 (39.1%) | 27 (7.8%) | *348* |  |
| **A20. It is important to me to have the opportunity of working on a part-time basis.** | | | | | | | |
| < 23 years | 30 (18.2%) | 49 (29.7%) | 35 (21.2%) | 34 (20.6%) | 17 (10.3%) | *165* | **0.003** ^ii^ |
| ≥ 23 years | 118 (34.0%) | 94 (27.1%) | 67 (19.3%) | 47 (13.5%) | 21 (6.1%) | *347* |  |
| **B23. General practice offers the opportunity to work on a part-time basis.** | | | | | | | |
| < 23 years | 47 (28.5%) | 73 (44.2%) | 25 (15.2%) | 17 (10.3%) | 3 (1.8%) | *165* | **0.005** ^ii^ |
| ≥ 23 years | 92 (26.4%) | 105 (30.2%) | 95 (27.3%) | 48 (13.8%) | 8 (2.3%) | *348* |  |
| **A21. It is important to me to reconcile family and professional life.** | | | | | | | |
| < 23 years | 106 (64.2%) | 39 (23.6%) | 13 (7.9%) | 4 (2.4%) | 3 (1.8%) | *165* | 0.459 ^ii^ |
| ≥ 23 years | 236 (68.2%) | 79 (22.8%) | 16 (4.6%) | 12 (3.5%) | 3 (0.9%) | *346* |  |
| **B24. General practice offers the possibility to reconcile family and professional life.** | | | | | | | |
| < 23 years | 66 (40.0%) | 77 (46.7%) | 17 (10.3%) | 3 (1.8%) | 2 (1.2%) | *165* | **0.001** ^ii^ |
| ≥ 23 years | 102 (29.4%) | 145 (41.8%) | 83 (23.9%) | 15 (4.3%) | 2 (0.6%) | *347* |  |
| **A25. It is important to me that my specialty has a positive perception in the medical community.** | | | | | | | |
| < 23 years | 44 (26.8%) | 57 (34.8%) | 30 (18.3%) | 31 (18.9%) | 2 (1.2%) | *164* | **0.012** ^ii^ |
| ≥ 23 years | 98 (28.2%) | 98 (28.2%) | 73 (21.0%) | 50 (14.4%) | 29 (8.3%) | *348* |  |
| **B28. General practice enjoys a positive perception in the medical community.** | | | | | | | |
| < 23 years | 9 (5.5%) | 40 (24.2%) | 70 (42.4%) | 32 (19.4%) | 14 (8.5%) | *165* | **0.024** ^ii^ |
| ≥ 23 years | 28 (8.1%) | 54 (15.7%) | 128 (37.2%) | 104 (30.2%) | 30 (8.7%) | *344* |  |
| **A26. It is important to me that my specialty is positively presented in the media.** | | | | | | | |
| < 23 years | 24 (14.5%) | 50 (30.3%) | 45 (27.3%) | 30 (18.2%) | 16 (9.7%) | *165* | 0.370 ^ii^ |
| ≥ 23 years | 63 (18.2%) | 91 (26.3%) | 80 (23.1%) | 62 (17.9%) | 50 (14.5%) | *346* |  |
| **B29. General practice is positively presented in the media.** | | | | | | | |
| < 23 years | 21 (12.7%) | 72 (43.6%) | 50 (30.3%) | 18 (10.9%) | 4 (2.4%) | *165* | **0.044** ^ii^ |
| ≥ 23 years | 38 (11.0%) | 108 (31.2%) | 132 (38.2%) | 53 (15.3%) | 15 (4.3%) | *346* |  |
| **A28. It is important to me that my specialty is appreciated by the political decision makers.** | | | | | | | |
| < 23 years | 46 (27.9%) | 58 (35.2%) | 27 (16.4%) | 19 (11.5%) | 15 (9.1%) | *165* | 0.330 ^ii^ |
| ≥ 23 years | 122 (35.2%) | 107 (30.8%) | 61 (17.6%) | 26 (7.5%) | 31 (8.9%) | *347* |  |
| **B31. General practice is appreciated by the political decision makers.** | | | | | | | |
| < 23 years | 15 (9.1%) | 45 (27.4%) | 53 (32.3%) | 34 (20.7%) | 17 (10.4%) | *164* | **0.011** ^ii^ |
| ≥ 23 years | 22 (6.4%) | 58 (16.8%) | 110 (31.8%) | 98 (28.3%) | 58 (16.8%) | *346* |  |

^ii^ Chi² Test

^§^ To keep the length of the tables reasonable, only items with significant results are reported for this part of the questionnaire. As these items were paired (‘It is important to me…’ – ‘In general practice…’), for each item with a significant result the respectively corresponding item is also reported (regardless whether it was significant or not).

### **Supplementary Table S3b**: Age-related significant^§^ differences of individual importance of aspects in the professional life (part A of the questionnaire) and possibilities in general practice (part B) – RESIDENTS

| **RESIDENTS** | **Yes, surely** | **Rather yes** | **Neutral** | **Rather no** | **Not at all** | ***n*** | **p-value** |
| --- | --- | --- | --- | --- | --- | --- | --- |
| **A15. It is important to me to have long-term relationships with the patients.** | | | | | | | |
| < 30 years | 11 (17.2%) | 16 (25.0%) | 29 (45.3%) | 7 (10.9%) | 1 (1.6%) | *64* | **0.022** ^ii^ |
| ≥ 30 years | 2 (5.3%) | 7 (18.4%) | 14 (36.8%) | 12 (31.6%) | 3 (7.9%) | *38* |  |
| **B17. In general practice one has long-term relationships with patients.** | | | | | | | |
| < 30 years | 55 (85.9%) | 8 (12.5%) | 1 (1.6%) | 0 (0.0%) | 0 (0.0%) | *64* | **0.008** ^ii^ |
| ≥ 30 years | 22 (56.4%) | 15 (38.5%) | 1 (2.6%) | 1 (2.6%) | 0 (0.0%) | *39* |  |
| **A24. It is important to me to realise private aims.** | | | | | | | |
| < 30 years | 42 (65.6%) | 20 (31.3%) | 2 (3.1%) | 0 (0.0%) | 0 (0.0%) | *64* | 0.078 ^ii^ |
| ≥ 30 years | 22 (56.4%) | 11 (28.2%) | 6 (15.4%) | 0 (0.0%) | 0 (0.0%) | *39* |  |
| **B27. General practice offers the possibility to realise private aims.** | | | | | | | |
| < 30 years | 6 (9.4%) | 31 (48.4%) | 24 (37.5%) | 2 (3.1%) | 1 (1.6%) | *64* | **0.012** ^ii^ |
| ≥ 30 years | 1 (2.6%) | 9 (23.1%) | 21 (53.8%) | 6 (15.4%) | 2 (5.1%) | *39* |  |
| **A25. It is important to me that my specialty has a positive perception in the medical community.** | | | | | | | |
| < 30 years | 17 (26.6%) | 28 (43.8%) | 14 (21.9%) | 2 (3.1%) | 3 (4.7%) | *64* | **0.004** ^ii^ |
| ≥ 30 years | 6 (15.8%) | 11 (28.9%) | 6 (15.8%) | 8 (21.1%) | 7 (18.4%) | *38* |  |
| **B28. General practice enjoys a positive perception in the medical community.** | | | | | | | |
| < 30 years | 2 (3.1%) | 10 (15.6%) | 17 (26.6%) | 27 (42.2%) | 8 (12.5%) | *64* | 0.961 ^ii^ |
| ≥ 30 years | 2 (5.1%) | 5 (12.8%) | 12 (30.8%) | 15 (38.5%) | 5 (12.8%) | *39* |  |
| **B35. I deem the profession of a GP as attractive.** | | | | | | | |
| < 30 years | 12 (18.8%) | 24 (37.5%) | 17 (26.6%) | 10 (15.6%) | 1 (1.6%) | *64* | **0.005** ^ii^ |
| ≥ 30 years | 3 (7.7%) | 6 (15.4%) | 14 (35.9%) | 10 (25.6%) | 6 (15.4%) | *39* |  |

^ii^ Chi² Test

^§^ To keep the length of the tables reasonable, only items with significant results are reported for this part of the questionnaire. As these items were paired (‘It is important to me…’ – ‘In general practice…’), for each item with a significant result the respectively corresponding item is also reported (regardless whether it was significant or not).

### **Supplementary Table S4a**: Nationality-related significant^§^ differences of individual importance of aspects in the professional life (part A of the questionnaire) and possibilities in general practice (part B) – MEDICAL STUDENTS

| **MEDICAL STUDENTS** | **Yes, surely** | **Rather yes** | **Neutral** | **Rather no** | **Not at all** | ***n*** | **p-value** |
| --- | --- | --- | --- | --- | --- | --- | --- |
| **A1. It is important to me to have a stable working position.** | | | | | | |  |
| Austria | 214 (75.6%) | 59 (20.8%) | 9 (3.2%) | 1 (0.4%) | 0 (0.0%) | *283* | **0.004** ^ii^ |
| Germany | 56 (57.1%) | 35 (35.7%) | 5 (5.1%) | 2 (2.0%) | 0 (0.0%) | *98* |  |
| South Tyrol | 97 (78.2%) | 24 (19.4%) | 3 (2.4%) | 0 (0.0%) | 0 (0.0%) | *124* |  |
| Others ^§§^ | 18 (94.7%) | 0 (0.0%) | 1 (5.3%) | 0 (0.0%) | 0 (0.0%) | *19* |  |
| **B1. General practice offers a stable working position.** | | | | | | |  |
| Austria | 184 (64.8%) | 82 (28.9%) | 15 (5.3%) | 3 (1.1%) | 0 (0.0%) | *284* | 0.398 ^ii^ |
| Germany | 55 (56.1%) | 36 (36.7%) | 6 (6.1%) | 1 (1.0%) | 0 (0.0%) | *98* |  |
| South Tyrol | 79 (63.7%) | 37 (29.8%) | 7 (5.6%) | 1 (0.8%) | 0 (0.0%) | *124* |  |
| Others ^§§^ | 9 (47.4%) | 6 (31.6%) | 3 (15.8%) | 1 (5.3%) | 0 (0.0%) | *19* |  |
| **A5. It is important to me to have a diversified daily routine.** | | | | | | | |
| Austria | 153 (54.3%) | 95 (33.7%) | 31 (11.0%) | 3 (1.1%) | 0 (0.0%) | *282* | 0.111 ^ii^ |
| Germany | 48 (49.0%) | 34 (34.7%) | 14 (14.3%) | 2 (2.0%) | 0 (0.0%) | *98* |  |
| South Tyrol | 69 (56.1%) | 42 (34.1%) | 11 (8.9%) | 1 (0.8%) | 0 (0.0%) | *123* |  |
| Others ^§§^ | 7 (36.8%) | 8 (42.1%) | 2 (10.5%) | 2 (10.5%) | 0 (0.0%) | *19* |  |
| **B5. General practice offers a diversified daily routine.** | | | | | | | |
| Austria | 82 (28.9%) | 72 (25.4%) | 90 (31.7%) | 32 (11.3%) | 8 (2.8%) | *284* | **0.011** ^ii^ |
| Germany | 16 (16.3%) | 40 (40.8%) | 27 (27.6%) | 15 (15.3%) | 0 (0.0%) | *98* |  |
| South Tyrol | 34 (27.4%) | 33 (26.6%) | 33 (26.6%) | 23 (18.5%) | 1 (0.8%) | *124* |  |
| Others ^§§^ | 3 (15.8%) | 9 (47.4%) | 6 (31.6%) | 0 (0.0%) | 1 (5.3%) | *19* |  |
| **A6. It is important to me to have few night duties.** | | | | | | | |
| Austria | 41 (14.5%) | 57 (20.1%) | 102 (36.0%) | 69 (24.4%) | 14 (4.9%) | *283* | **0.020** ^ii^ |
| Germany | 18 (18.4%) | 30 (30.6%) | 24 (24.5%) | 15 (15.3%) | 11 (11.2%) | *98* |  |
| South Tyrol | 28 (22.8%) | 26 (21.1%) | 43 (35.0%) | 22 (17.9%) | 4 (3.3%) | *123* |  |
| Others ^§§^ | 2 (10.5%) | 7 (36.8%) | 4 (21.1%) | 4 (21.1%) | 2 (10.5%) | *19* |  |
| **B7. In general practice one has few night duties.** | | | | | | | |
| Austria | 151 (53.2%) | 83 (29.2%) | 39 (13.7%) | 11 (3.9%) | 0 (0.0%) | *284* | 0.522 ^ii^ |
| Germany | 58 (59.2%) | 30 (30.6%) | 8 (8.2%) | 2 (2.0%) | 0 (0.0%) | *98* |  |
| South Tyrol | 68 (54.8%) | 37 (29.8%) | 14 (11.3%) | 4 (3.2%) | 1 (0.8%) | *124* |  |
| Others ^§§^ | 7 (36.8%) | 8 (42.1%) | 2 (10.5%) | 2 (10.5%) | 0 (0.0%) | *19* |  |
| **A7. It is important to me to have good opportunities of income.** | | | | | | | |
| Austria | 113 (40.1%) | 127 (45.0%) | 33 (11.7%) | 7 (2.5%) | 2 (0.7%) | *282* | 0.552 ^ii^ |
| Germany | 31 (32.0%) | 52 (53.6%) | 12 (12.4%) | 1 (1.0%) | 1 (1.0%) | *97* |  |
| South Tyrol | 61 (49.6%) | 48 (39.0%) | 11 (8.9%) | 3 (2.4%) | 0 (0.0%) | *123* |  |
| Others ^§§^ | 9 (47.4%) | 9 (47.4%) | 1 (5.3%) | 0 (0.0%) | 0 (0.0%) | *19* |  |
| **B8. General practice offers good opportunities of income.** | | | | | | | |
| Austria | 41 (14.4%) | 68 (23.9%) | 93 (32.7%) | 63 (22.2%) | 19 (6.7%) | *284* | **0.016** ^ii^ |
| Germany | 14 (14.3%) | 31 (31.6%) | 36 (36.7%) | 15 (15.3%) | 2 (2.0%) | *98* |  |
| South Tyrol | 32 (25.8%) | 35 (28.2%) | 42 (33.9%) | 13 (10.5%) | 2 (1.6%) | *124* |  |
| Others ^§§^ | 5 (26.3%) | 6 (31.6%) | 5 (26.3%) | 2 (10.5%) | 1 (5.3%) | *19* |  |
| **A8. It is important to me to have an assured income.** | | | | | | | |
| Austria | 210 (74.5%) | 64 (22.7%) | 7 (2.5%) | 1 (0.4%) | 0 (0.0%) | *282* | 0.208 ^ii^ |
| Germany | 62 (63.3%) | 29 (29.6%) | 6 (6.1%) | 1 (1.0%) | 0 (0.0%) | *98* |  |
| South Tyrol | 94 (78.3%) | 23 (19.2%) | 2 (1.7%) | 1 (0.8%) | 0 (0.0%) | *120* |  |
| Others ^§§^ | 17 (89.5%) | 2 (10.5%) | 0 (0.0%) | 0 (0.0%) | 0 (0.0%) | *19* |  |
| **B9. In general practice one has an assured income.** | | | | | | | |
| Austria | 91 (32.3%) | 113 (40.1%) | 67 (23.8%) | 11 (3.9%) | 0 (0.0%) | *282* | **0.007** ^ii^ |
| Germany | 26 (26.5%) | 45 (45.9%) | 20 (20.4%) | 7 (7.1%) | 0 (0.0%) | *98* |  |
| South Tyrol | 62 (50.0%) | 45 (36.3%) | 12 (9.7%) | 5 (4.0%) | 0 (0.0%) | *124* |  |
| Others ^§§^ | 6 (31.6%) | 9 (47.4%) | 3 (15.8%) | 1 (5.3%) | 0 (0.0%) | *19* |  |
| **A12. It is important to me to conduct preventive activities.** | | | | | | | |
| Austria | 134 (47.7%) | 67 (23.8%) | 54 (19.2%) | 23 (8.2%) | 3 (1.1%) | *281* | **0.036** ^ii^ |
| Germany | 33 (33.7%) | 36 (36.7%) | 14 (14.3%) | 14 (14.3%) | 1 (1.0%) | *98* |  |
| South Tyrol | 69 (56.1%) | 32 (26.0%) | 14 (11.4%) | 8 (6.5%) | 0 (0.0%) | *123* |  |
| Others ^§§^ | 11 (61.1%) | 4 (22.2%) | 3 (16.7%) | 0 (0.0%) | 0 (0.0%) | *18* |  |
| **B13. General practice offers the possibility to conduct preventive activities.** | | | | | | | |
| Austria | 237 (83.7%) | 38 (13.4%) | 6 (2.1%) | 2 (0.7%) | 0 (0.0%) | *283* | 0.873 ^ii^ |
| Germany | 77 (79.4%) | 19 (19.6%) | 1 (1.0%) | 0 (0.0%) | 0 (0.0%) | *97* |  |
| South Tyrol | 98 (79.0%) | 23 (18.5%) | 2 (1.6%) | 1 (0.8%) | 0 (0.0%) | *124* |  |
| Others ^§§^ | 16 (84.2%) | 3 (15.8%) | 0 (0.0%) | 0 (0.0%) | 0 (0.0%) | *19* |  |
| **A13. It is important to me to be exposed to a low physical burden in my professional life.** | | | | | | | |
| Austria | 12 (4.2%) | 46 (16.3%) | 80 (28.3%) | 92 (32.5%) | 53 (18.7%) | *283* | 0.865 ^ii^ |
| Germany | 5 (5.1%) | 20 (20.4%) | 32 (32.7%) | 29 (29.6%) | 12 (12.2%) | *98* |  |
| South Tyrol | 6 (4.8%) | 23 (18.5%) | 42 (33.9%) | 38 (30.6%) | 15 (12.1%) | *124* |  |
| Others ^§§^ | 1 (5.3%) | 5 (26.3%) | 5 (26.3%) | 6 (31.6%) | 2 (10.5%) | *19* |  |
| **B14. In general practice one is exposed to a low physical burden.** | | | | | | | |
| Austria | 48 (17.0%) | 95 (33.6%) | 103 (36.4%) | 33 (11.7%) | 4 (1.4%) | *283* | **0.008** ^ii^ |
| Germany | 22 (22.4%) | 38 (28.8%) | 27 (27.6%) | 8 (8.2%) | 3 (3.1%) | *98* |  |
| South Tyrol | 41 (33.1%) | 40 (32.3%) | 37 (29.8%) | 6 (4.8%) | 0 (0.0%) | *124* |  |
| Others ^§§^ | 8 (42.1%) | 7 (36.8%) | 3 (15.8%) | 1 (5.3%) | 0 (0.0%) | *19* |  |
| **A14. It is important to me to be exposed to a low psychological burden in my professional life.** | | | | | | | |
| Austria | 19 (6.7%) | 55 (19.5%) | 101 (35.8%) | 84 (29.8%) | 23 (8.2%) | *282* | 0.096 ^ii^ |
| Germany | 15 (15.3%) | 23 (23.5%) | 28 (28.6%) | 25 (25.5%) | 7 (7.1%) | *98* |  |
| South Tyrol | 16 (13.1%) | 34 (27.9%) | 42 (34.4%) | 24 (19.7%) | 6 (4.9%) | *122* |  |
| Others ^§§^ | 2 (10.5%) | 7 (36.8%) | 6 (31.6%) | 4 (21.1%) | 0 (0.0%) | *19* |  |
| **B15. In general practice one is exposed to a low psychological burden.** | | | | | | | |
| Austria | 8 (2.8%) | 30 (10.6%) | 118 (41.5%) | 103 (36.3%) | 25 (8.8%) | *284* | **0.004** ^ii^ |
| Germany | 4 (4.1%) | 12 (12.2%) | 44 (44.9%) | 35 (35.7%) | 3 (3.1%) | *98* |  |
| South Tyrol | 12 (9.8%) | 17 (13.8%) | 43 (35.0%) | 45 (36.6%) | 6 (4.9%) | *123* |  |
| Others ^§§^ | 2 (10.5%) | 7 (36.8%) | 4 (21.1%) | 6 (31.6%) | 0 (0.0%) | *19* |  |
| **A16. It is important to me to conduct emergency medicine activities.** | | | | | | | |
| Austria | 83 (29.4%) | 72 (25.5%) | 54 (19.1%) | 52 (18.4%) | 21 (7.4%) | *282* | 0.438 ^ii^ |
| Germany | 29 (29.6%) | 24 (24.5%) | 19 (19.4%) | 20 (20.4%) | 6 (6.1%) | *98* |  |
| South Tyrol | 23 (18.5%) | 36 (29.0%) | 36 (29.0%) | 23 (18.5%) | 6 (4.8%) | *124* |  |
| Others ^§§^ | 5 (26.3%) | 5 (26.3%) | 6 (31.6%) | 3 (15.8%) | 0 (0.0%) | *19* |  |
| **B19. General practice offers the possibility to conduct emergency medicine activities.** | | | | | | | |
| Austria | 50 (17.7%) | 85 (30.1%) | 107 (37.9%) | 36 (12.8%) | 4 (1.4%) | *282* | **0.001** ^ii^ |
| Germany | 11 (11.2%) | 30 (30.6%) | 39 (39.8%) | 14 (14.3%) | 4 (4.1%) | *98* |  |
| South Tyrol | 11 (8.9%) | 22 (17.7%) | 50 (40.3%) | 32 (25.8%) | 9 (7.3%) | *124* |  |
| Others ^§§^ | 3 (15.8%) | 6 (31.6%) | 9 (47.4%) | 1 (5.3%) | 0 (0.0%) | *19* |  |
| **A21. It is important to me to reconcile family and professional life.** | | | | | | | |
| Austria | 194 (68.6%) | 65 (23.0%) | 9 (3.2%) | 11 (3.9%) | 4 (1.4%) | *283* | **0.014** ^ii^ |
| Germany | 55 (56.1%) | 28 (28.6%) | 10 (10.2%) | 3 (3.1%) | 2 (2.0%) | *98* |  |
| South Tyrol | 87 (71.3%) | 25 (20.5%) | 8 (6.6%) | 2 (1.6%) | 0 (0.0%) | *122* |  |
| Others ^§§^ | 14 (73.7%) | 1 (5.3%) | 4 (21.1%) | 0 (0.0%) | 0 (0.0%) | *19* |  |
| **B24. General practice offers the possibility to reconcile family and professional life.** | | | | | | | |
| Austria | 83 (29.3%) | 133 (47.0%) | 51 (18.0%) | 12 (4.2%) | 4 (1.4%) | *283* | 0.208 ^ii^ |
| Germany | 30 (30.6%) | 40 (40.8%) | 23 (23.5%) | 5 (5.1%) | 0 (0.0%) | *98* |  |
| South Tyrol | 52 (41.9%) | 48 (38.7%) | 22 (17.7%) | 1 (0.8%) | 1 (0.8%) | *124* |  |
| Others ^§§^ | 7 (36.8%) | 6 (31.6%) | 6 (31.6%) | 0 (0.0%) | 0 (0.0%) | *19* |  |
| **A25. It is important to me that my specialty has a positive perception in the medical community.** | | | | | | | |
| Austria | 91 (32.3%) | 81 (28.7%) | 55 (19.5%) | 40 (14.2%) | 15 (5.3%) | *282* | **0.025** ^ii^ |
| Germany | 15 (15.3%) | 30 (30.6%) | 22 (22.4%) | 19 (19.4%) | 12 (12.2%) | *98* |  |
| South Tyrol | 32 (25.8%) | 44 (35.5%) | 25 (20.2%) | 18 (14.5%) | 5 (4.0%) | *124* |  |
| Others ^§§^ | 9 (47.4%) | 4 (21.1%) | 2 (10.5%) | 4 (21.1%) | 0 (0.0%) | *19* |  |
| **B28. General practice enjoys a positive perception in the medical community.** | | | | | | | |
| Austria | 14 (5.0%) | 53 (18.9%) | 107 (38.1%) | 86 (30.6%) | 21 (7.5%) | *281* | 0.122 ^ii^ |
| Germany | 9 (9.2%) | 14 (14.3%) | 38 (38.8%) | 29 (29.6%) | 8 (8.2%) | *98* |  |
| South Tyrol | 12 (9.7%) | 24 (19.4%) | 53 (42.7%) | 22 (17.7%) | 13 (10.5%) | *124* |  |
| Others ^§§^ | 3 (16.7%) | 5 (27.8%) | 7 (38.9%) | 1 (5.6%) | 2 (11.1%) | *18* |  |
| **A27. It is important to me that my specialty is appreciated by the patients.** | | | | | | | |
| Austria | 127 (44.9%) | 100 (35.3%) | 40 (14.1%) | 13 (4.6%) | 3 (1.1%) | *283* | 0.118 ^ii^ |
| Germany | 30 (30.6%) | 40 (40.8%) | 17 (17.3%) | 7 (7.1%) | 4 (4.1%) | *98* |  |
| South Tyrol | 52 (42.3%) | 48 (39.0%) | 13 (10.6%) | 6 (4.9%) | 4 (3.3%) | *123* |  |
| Others ^§§^ | 13 (68.4%) | 5 (26.3%) | 1 (5.3%) | 0 (0.0%) | 0 (0.0%) | *19* |  |
| **B30. General practice is appreciated by the patients.** | | | | | | | |
| Austria | 107 (37.9%) | 79 (28.0%) | 68 (24.1%) | 20 (7.1%) | 8 (2.8%) | *282* | **0.037** ^ii^ |
| Germany | 33 (33.7%) | 38 (38.8%) | 18 (18.4%) | 7 (7.1%) | 2 (2.0%) | *98* |  |
| South Tyrol | 28 (22.8%) | 34 (27.6%) | 41 (33.3%) | 15 (12.2%) | 5 (4.1%) | *123* |  |
| Others ^§§^ | 6 (31.6%) | 9 (47.4%) | 4 (21.1%) | 0 (0.0%) | 0 (0.0%) | *19* |  |
| **A29. It is important to me that my specialty has a high significance/status during medical education and specialisation.** | | | | | | | |
| Austria | 93 (33.0%) | 98 (34.8%) | 60 (21.3%) | 27 (9.6%) | 4 (1.4%) | *282* | **0.039** ^ii^ |
| Germany | 23 (23.7%) | 31 (32.0%) | 29 (29.9%) | 10 (10.3%) | 4 (4.1%) | *97* |  |
| South Tyrol | 46 (37.1%) | 37 (29.8%) | 25 (20.2%) | 8 (6.5%) | 8 (6.5%) | *124* |  |
| Others ^§§^ | 11 (57.9%) | 5 (26.3%) | 3 (15.8%) | 0 (0.0%) | 0 (0.0%) | *19* |  |
| **B32. General practice has a high significance/status during medical education and specialisation.** | | | | | | | |
| Austria | 12 (4.2%) | 44 (15.5%) | 97 (34.3%) | 98 (34.6%) | 32 (11.3%) | *283* | 0.564 ^ii^ |
| Germany | 4 (4.1%) | 16 (16.5%) | 36 (37.1%) | 30 (30.9%) | 11 (11.3%) | *97* |  |
| South Tyrol | 4 (3.2%) | 26 (21.0%) | 43 (34.7%) | 40 (32.3%) | 11 (8.9%) | *124* |  |
| Others ^§§^ | 3 (15.8%) | 3 (15.8%) | 8 (42.1%) | 3 (15.8%) | 2 (10.5%) | *19* |  |
| **B6. In general practice one makes independent, self-reliant decisions.** | | | | | | | |
| Austria | 197 (69.4%) | 65 (22.9%) | 16 (5.6%) | 6 (2.1%) | 0 (0.0%) | *284* | **0.004** ^ii^ |
| Germany | 67 (68.4%) | 23 (23.5%) | 8 (8.2%) | 0 (0.0%) | 0 (0.0%) | *98* |  |
| South Tyrol | 59 (47.6%) | 43 (34.7%) | 16 (12.9%) | 6 (4.8%) | 0 (0.0%) | *124* |  |
| Others ^§§^ | 13 (68.4%) | 5 (26.3%) | 1 (5.3%) | 0 (0.0%) | 0 (0.0%) | *19* |  |
| **B18. In general practice one works in a team.** | | | | | | | |
| Austria | 51 (18.0%) | 65 (22.9%) | 85 (29.9%) | 66 (23.2%) | 17 (6.0%) | *284* | <**0.001** ^ii^ |
| Germany | 27 (27.6%) | 22 (22.4%) | 27 (27.6%) | 20 (20.4%) | 2 (2.0%) | *98* |  |
| South Tyrol | 8 (6.5%) | 11 (8.9%) | 45 (36.3%) | 41 (33.1%) | 19 (15.3%) | *124* |  |
| Others ^§§^ | 5 (26.3%) | 5 (26.3%) | 5 (26.3%) | 4 (21.1%) | 0 (0.0%) | *19* |  |

^ii^ Chi² Test

^§^ To keep the length of the tables reasonable, only items with significant results are reported for this part of the questionnaire. As these items were paired (‘It is important to me…’ – ‘In general practice…’), for each item with a significant result the respectively corresponding item is also reported (regardless whether it was significant or not).

^§§^ Other nationalities include: Italian regions except South Tyrol, EU-states, Non-EU-states

### **Supplementary Table S4b**: Nationality-related significant^§^ differences of individual importance of aspects in the professional life (part A of the questionnaire) and possibilities in general practice (part B) – RESIDENTS

| **RESIDENTS** | **Yes, surely** | **Rather yes** | **Neutral** | **Rather no** | **Not at all** | ***n*** | **p-value** |
| --- | --- | --- | --- | --- | --- | --- | --- |
| **A11. It is important to me to work according to the currently available scientific state of the art.** | | | | | | | |
| Austria | 23 (37.7%) | 30 (49.2%) | 5 (8.2%) | 2 (3.3%) | 1 (1.6%) | *61* | 0.710 ^ii^ |
| Germany | 7 (43.8%) | 6 (37.5%) | 3 (18.8%) | 0 (0.0%) | 0 (0.0%) | *16* |  |
| South Tyrol | 12 (60.0%) | 8 (40.0%) | 0 (0.0%) | 0 (0.0%) | 0 (0.0%) | *20* |  |
| Others ^§§^ | 2 (40.0%) | 3 (60.0%) | 0 (0.0%) | 0 (0.0%) | 0 (0.0%) | *5* |  |
| **B12. In general practice one works according to the currently available scientific state of the art.** | | | | | | | |
| Austria | 1 (1.6%) | 11 (18.0%) | 33 (54.1%) | 14 (23.0%) | 2 (3.3%) | *61* | **0.030** ^ii^ |
| Germany | 0 (0.0%) | 4 (25.0%) | 8 (50.0%) | 3 (18.8%) | 1 (6.3%) | *16* |  |
| South Tyrol | 2 (9.5%) | 7 (33.3%) | 5 (23.8%) | 6 (28.6%) | 1 (4.8%) | *21* |  |
| Others ^§§^ | 2 (40.0%) | 1 (20.0%) | 2 (40.0%) | 0 (0.0%) | 0 (0.0%) | *5* |  |
| **A14. It is important to me to be exposed to a low psychological burden in my professional life.** | | | | | | | |
| Austria | 8 (13.1%) | 11 (18.0%) | 16 (26.2%) | 21 (34.4%) | 5 (8.2%) | *61* | **0.016** ^ii^ |
| Germany | 9 (56.3%) | 4 (25.0%) | 3 (18.8%) | 0 (0.0%) | 0 (0.0%) | *16* |  |
| South Tyrol | 2 (9.5%) | 4 (19.0%) | 8 (38.1%) | 5 (23.8%) | 2 (9.5%) | *21* |  |
| Others ^§§^ | 3 (60.0%) | 1 (20.0%) | 1 (20.0%) | 0 (0.0%) | 0 (0.0%) | *5* |  |
| **B15. In general practice one is exposed to a low psychological burden.** | | | | | | | |
| Austria | 0 (0.0%) | 3 (4.9%) | 22 (36.1%) | 28 (45.9%) | 8 (13.1%) | *61* | 0.979 ^ii^ |
| Germany | 0 (0.0%) | 2 (12.5%) | 5 (31.3%) | 7 (43.8%) | 2 (12.5%) | *16* |  |
| South Tyrol | 0 (0.0%) | 1 (4.8%) | 6 (28.6%) | 10 (47.6%) | 4 (19.0%) | *21* |  |
| Others ^§§^ | 0 (0.0%) | 0 (0.0%) | 2 (40.0%) | 2 (40.0%) | 1 (20.0%) | *5* |  |
| **A16. It is important to me to conduct emergency medicine activities.** | | | | | | | |
| Austria | 21 (35.0%) | 14 (23.3%) | 11 (18.3%) | 10 (16.7%) | 4 (6.7%) | *60* | 0.943 ^ii^ |
| Germany | 5 (31.3%) | 3 (18.8%) | 4 (25.0%) | 3 (18.8%) | 1 (6.3%) | *16* |  |
| South Tyrol | 7 (33.3%) | 8 (38.1%) | 1 (4.8%) | 4 (19.0%) | 1 (4.8%) | *21* |  |
| Others ^§§^ | 1 (20.0%) | 2 (40.0%) | 1 (20.0%) | 1 (20.0%) | 0 (0.0%) | *5* |  |
| **B19. General practice offers the possibility to conduct emergency medicine activities.** | | | | | | | |
| Austria | 8 (13.1%) | 23 (37.7%) | 23 (37.7%) | 7 (11.5%) | 0 (0.0%) | *61* | **0.007** ^ii^ |
| Germany | 2 (12.5%) | 5 (31.3%) | 7 (43.8%) | 1 (6.3%) | 1 (6.3%) | *16* |  |
| South Tyrol | 0 (0.0%) | 3 (14.3%) | 15 (71.4%) | 3 (14.3%) | 0 (0.0%) | *21* |  |
| Others ^§§^ | 3 (60.0%) | 1 (20.0%) | 1 (20.0%) | 0 (0.0%) | 0 (0.0%) | *5* |  |
| **A22. It is important to me that my profession earns public recognition.** | | | | | | | |
| Austria | 1 (1.6%) | 21 (34.4%) | 23 (37.7%) | 10 (16.4%) | 6 (9.8%) | *61* | **0.016** ^ii^ |
| Germany | 0 (0.0%) | 5 (31.3%) | 5 (31.3%) | 6 (37.5%) | 0 (0.0%) | *16* |  |
| South Tyrol | 3 (14.3%) | 8 (38.1%) | 7 (33.3%) | 2 (9.5%) | 1 (4.8%) | *21* |  |
| Others ^§§^ | 1 (4.8%) | 0 (0.0%) | 1 (20.0%) | 1 (20.0%) | 1 (20.0%) | *5* |  |
| **B25. General practice is a profession which earns public recognition.** | | | | | | | |
| Austria | 8 (13.1%) | 17 (27.9%) | 22 (36.1%) | 8 (13.1%) | 6 (9.8%) | *61* | 0.677 ^ii^ |
| Germany | 3 (18.8%) | 4 (25.0%) | 6 (37.5%) | 2 (12.5%) | 1 (6.3%) | *16* |  |
| South Tyrol | 3 (14.3%) | 12 (57.1%) | 4 (19.0%) | 1 (4.8%) | 1 (4.8%) | *21* |  |
| Others ^§§^ | 0 (0.0%) | 2 (40.0%) | 2 (40.0%) | 1 (20.0%) | 0 (0.0%) | *5* |  |

^ii^ Chi² Test

^§^ To keep the length of the tables reasonable, only items with significant results are reported for this part of the questionnaire. As these items were paired (‘It is important to me…’ – ‘In general practice…’), for each item with a significant result the respectively corresponding item is also reported (regardless whether it was significant or not).

^§§^ Other nationalities include: Italian regions except South Tyrol, EU-states, Non-EU-states
