# Supplementary material for: Attitudes towards career choice and general practice: a cross-sectional survey of medical students and residents in Tyrol, Austria
Source: BMC Med Educ. 2024 Mar 15;24:294. doi: 10.1186/s12909-024-05205-8 (PMC10943776; doi:10.1186/s12909-024-05205-8)
Supplement: Supplementary file 3 — Supplementary Material 3 [file 12909_2024_5205_MOESM3_ESM.docx]

### **Supplementary Table S5a**: Gender-related analyses regarding interest in general practice, feeling of preparedness to work as a GP, specialist qualification, and personal preferences regarding various professional profiles and working circumstances (part C of the questionnaire) – MEDICAL STUDENTS

| **MEDICAL STUDENTS** | | **Only general practice** | | | | **General practice or other specialty** | | | **Only other specialty** | | | **Don’t know** | ***n*** | **p-value** |
| --- | --- | --- | --- | --- | --- | --- | --- | --- | --- | --- | --- | --- | --- | --- |
| **C1. Currently planned specialty choice** | | | | | | | | | | | | | | |
| Females | | 7 (2.4%) | | | | 136 (45.9%) | | | 116 (39.2%) | | | 37 (12.5%) | *296* | 0.431 ^ii^ |
| Males | | 10 (4.4%) | | | | 96 (41.9%) | | | 98 (42.8%) | | | 25 (10.9%) | *229* |  |
|  | | **Yes, surely** | | **Rather yes** | | | **Neutral** | | | **Rather no** | | **Not at all** | ***n*** | **p-value** |
| **C2. Interest in general practice as a specialty for the future professional life** | | | | | | | | | | | | | | |
| Females | | 47 (15.9%) | | 74 (25.1%) | | | 74 (25.1%) | | | 73 (24.7%) | | 27 (9.2%) | *295* | 0.392 ^ii^ |
| Males | | 27 (11.8%) | | 59 (25.8%) | | | 72 (31.4%) | | | 49 (21.4%) | | 22 (9.6%) | *229* |  |
|  | **Very good** | | **Good** | | **Neutral** | | | **Bad** | | | **Very bad** | **No exp.** | ***n*** | **p-value** |
| **C3a. Quality of the practical experiences in general practice during medical school** | | | | | | | | | | | | | | |
| Females | 66 (22.4%) | | 58 (19.7%) | | 25 (8.5%) | | | 7 (2.4%) | | | 0 (0.0%) | 138 (46.9%) | *296* | 0.314 ^ii^ |
| Males | 43 (18.7%) | | 36 (15.7%) | | 19 (8.3%) | | | 6 (2.6%) | | | 2 (0.9%) | 124 (53.9%) | *230* |  |
| **C3b. Quality of the theoretical experiences in general practice during medical school** | | | | | | | | | | | | | | |
| Females | 35 (11.9%) | | 115 (39.0%) | | 110 (37.3%) | | | 22 (7.5%) | | | 5 (1.7%) | 8 (2.7%) | *295* | **0.003** ^ii^ |
| Males | 20 (8.7%) | | 89 (38.7%) | | 63 (27.4%) | | | 36 (15.7%) | | | 10 (4.3%) | 12 (5.2%) | *230* |  |
|  | | **Yes, surely** | | **Rather yes** | | | **Neutral** | | | **Rather no** | | **Not at all** | ***n*** | **p-value** |
| **C4a. I feel well prepared to work as a GP by medical school** | | | | | | | | | | | | | | |
| Females | | 8 (2.7%) | | 62 (21.2%) | | | 118 (40.3%) | | | 90 (30.7%) | | 15 (5.1%) | *293* | 0.515 ^ii^ |
| Males | | 3 (1.3%) | | 58 (25.4%) | | | 81 (35.5%) | | | 72 (31.6%) | | 14 (6.1%) | *228* |  |
| **C5. A specialist qualification would increase the attractiveness of general practice** | | | | | | | | | | | | | | |
| Females | | 52 (17.7%) | | 83 (28.2%) | | | 49 (16.7%) | | | 63 (21.4%) | | 47 (16.0%) | *294* | 0.495 ^ii^ |
| Males | | 33 (14.3%) | | 77 (33.5%) | | | 39 (17.0%) | | | 40 (17.4%) | | 41 (17.8%) | *230* |  |
|  | | **Yes, surely** | | **Rather yes** | | | **Neutral** | | | **Rather no** | | **Not at all** | ***n*** | **p-value** |
| **C6. A specialist qualification is more attractive to me than general practice, because …** | | | | | | | | | | | | | | |
| **… without any specialist qualification, I would be a ‘lesser’ physician** | | | | | | | | | | | | | | |
| Females | | 17 (5.8%) | | 43 (14.7%) | | | 40 (13.7%) | | | 78 (26.6%) | | 115 (39.2%) | *293* | 0.670 ^ii^ |
| Males | | 11 (4.8%) | | 44 (19.1%) | | | 27 (11.7%) | | | 63 (27.4%) | | 85 (37.0%) | *230* |  |
| **… I can get higher reputation as a specialist** | | | | | | | | | | | | | | |
| Females | | 38 (13.1%) | | 67 (23.0%) | | | 40 (13.7%) | | | 64 (22.0%) | | 82 (28.2%) | *291* | 0.781 ^ii^ |
| Males | | 28 (12.2%) | | 51 (22.3%) | | | 40 (17.5%) | | | 44 (19.2%) | | 66 (28.8%) | *229* |  |
| **… teamwork is easier as a specialist** | | | | | | | | | | | | | | |
| Females | | 44 (15.0%) | | 74 (25.3%) | | | 64 (21.8%) | | | 58 (19.8%) | | 53 (18.1%) | *293* | 0.072 ^ii^ |
| Males | | 19 (8.3%) | | 72 (31.6%) | | | 43 (18.9%) | | | 56 (24.6%) | | 38 (16.7%) | *228* |  |
| **… I have better possibilities to work in research** | | | | | | | | | | | | | | |
| Females | | 64 (21.9%) | | 85 (29.1%) | | | 54 (18.5%) | | | 48 (16.4%) | | 41 (14.0%) | *292* | 0.281 ^ii^ |
| Males | | 52 (22.6%) | | 79 (34.3%) | | | 27 (11.7%) | | | 37 (16.1%) | | 35 (15.2%) | *230* |  |
| **… I have a higher income** | | | | | | | | | | | | | | |
| Females | | 75 (25.7%) | | 86 (29.5%) | | | 60 (20.5%) | | | 39 (13.4%) | | 32 (11.0%) | *292* | 0.561 ^ii^ |
| Males | | 52 (22.7%) | | 73 (31.9%) | | | 39 (17.0%) | | | 40 (17.5%) | | 25 (10.9%) | *229* |  |
| **… there are clearer content-related demarcations of the field** | | | | | | | | | | | | | | |
| Females | | 76 (25.9%) | | 98 (33.3%) | | | 52 (17.7%) | | | 41 (13.9%) | | 27 (9.2%) | *294* | 0.622 ^ii^ |
| Males | | 51 (22.3%) | | 71 (31.0%) | | | 44 (19.2%) | | | 42 (18.3%) | | 21 (9.2%) | *229* |  |
| **… I am facing more challenging medical topics** | | | | | | | | | | | | | | |
| Females | | 61 (20.7%) | | 53 (18.0%) | | | 65 (22.1%) | | | 77 (26.2%) | | 38 (12.9%) | *294* | 0.282 ^ii^ |
| Males | | 54 (23.6%) | | 56 (24.5%) | | | 45 (19.7%) | | | 49 (21.4%) | | 25 (10.9%) | *229* |  |
| **… I have better opportunities of career** | | | | | | | | | | | | | | |
| Females | | 75 (25.5%) | | 93 (31.6%) | | | 54 (18.4%) | | | 40 (13.6%) | | 32 (10.9%) | *294* | 0.967 ^ii^ |
| Males | | 61 (26.6%) | | 71 (31.0%) | | | 46 (20.1%) | | | 29 (12.7%) | | 22 (9.6%) | *229* |  |
| **… I can extend my knowledge in a more targeted way** | | | | | | | | | | | | | | |
| Females | | 135 (45.9%) | | 77 (26.2%) | | | 48 (16.3%) | | | 23 (7.8%) | | 11 (3.7%) | *294* | 0.201 ^ii^ |
| Males | | 107 (46.5%) | | 71 (30.9%) | | | 23 (10.0%) | | | 16 (7.0%) | | 13 (5.7%) | *230* |  |
|  | | **Yes** | | | | | **No** | | | | | **Don’t know** | ***n*** | **p-value** |
| **C7. I would preferably work …** | | | | | | | | | | | | | | |
| **… as an independent physician in a group office** | | | | | | | | | | | | | | |
| Females | | 201 (68.1%) | | | | | 46 (15.6%) | | | | | 48 (16.3%) | *295* | 0.052 ^ii^ |
| Males | | 135 (58.7%) | | | | | 53 (23.0%) | | | | | 42 (18.3%) | *230* |  |
| **… as an independent physician in a single-handed office** | | | | | | | | | | | | | | |
| Females | | 114 (38.6%) | | | | | 133 (45.1%) | | | | | 48 (16.3%) | *295* | **0.007** ^ii^ |
| Males | | 117 (51.1%) | | | | | 74 (32.3%) | | | | | 38 (16.6%) | *229* |  |
| **… as a physician in the hospital setting** | | | | | | | | | | | | | | |
| Females | | 191 (65.0%) | | | | | 61 (20.7%) | | | | | 42 (14.3%) | *294* | 0.055 ^ii^ |
| Males | | 168 (73.7%) | | | | | 41 (18.0%) | | | | | 19 (8.3%) | *228* |  |
| **… as an employed physician in another physician’s office** | | | | | | | | | | | | | | |
| Females | | 120 (41.0%) | | | | | 108 (36.9%) | | | | | 65 (22.2%) | *293* | **<0.001** ^ii^ |
| Males | | 53 (23.3%) | | | | | 143 (63.0%) | | | | | 31 (13.7%) | *227* |  |
| **… as a GP in a multiprofessional team (e.g. Primary Healthcare Center)** | | | | | | | | | | | | | | |
| Females | | 132 (44.7%) | | | | | 88 (29.8%) | | | | | 75 (25.4%) | *295* | **0.017** ^ii^ |
| Males | | 90 (39.6%) | | | | | 94 (41.4%) | | | | | 43 (18.9%) | *227* |  |
| **… as a GP in the primary care setting** | | | | | | | | | | | | | | |
| Females | | 108 (36.9%) | | | | | 114 (38.9%) | | | | | 71 (24.2%) | *293* | 0.691 ^ii^ |
| Males | | 88 (38.6%) | | | | | 92 (40.4%) | | | | | 48 (21.1%) | *228* |  |
| **… as a ward physician in a hospital** | | | | | | | | | | | | | | |
| Females | | 64 (21.7%) | | | | | 172 (58.3%) | | | | | 59 (20.0%) | *295* | 0.798 ^ii^ |
| Males | | 47 (20.6%) | | | | | 130 (57.0%) | | | | | 51 (22.4%) | *228* |  |
| **… as a school physician** | | | | | | | | | | | | | | |
| Females | | 33 (11.2%) | | | | | 218 (73.9%) | | | | | 44 (14.9%) | *295* | **0.019** ^ii^ |
| Males | | 29 (12.7%) | | | | | 183 (80.3%) | | | | | 16 (7.0%) | *228* |  |
| **… as a public health officer** | | | | | | | | | | | | | | |
| Females | | 32 (10.9%) | | | | | 220 (75.1%) | | | | | 41 (14.0%) | *293* | 0.392 ^ii^ |
| Males | | 28 (12.3%) | | | | | 176 (77.5%) | | | | | 23 (10.1%) | *227* |  |
| **… as a physician with insurance companies or health insurances** | | | | | | | | | | | | | | |
| Females | | 9 (3.1%) | | | | | 253 (85.8%) | | | | | 33 (11.2%) | *295* | 0.135 ^ii^ |
| Males | | 12 (5.3%) | | | | | 200 (87.7%) | | | | | 16 (7.0%) | *228* |  |
| **… in a scientific career** | | | | | | | | | | | | | | |
| Females | | 81 (27.6%) | | | | | 164 (55.8%) | | | | | 49 (16.7%) | *294* | 0.079 ^ii^ |
| Males | | 82 (36.1%) | | | | | 106 (46.7%) | | | | | 39 (17.2%) | *227* |  |
| **… in a non-medical career (e.g. pharmaceutical industry, economy)** | | | | | | | | | | | | | | |
| Females | | 16 (5.4%) | | | | | 241 (81.7%) | | | | | 38 (12.9%) | *295* | **0.025** ^ii^ |
| Males | | 27 (12.0%) | | | | | 169 (75.1%) | | | | | 29 (12.9%) | *225* |  |
| **C8. I would like to work …** | | | | | | | | | | | | | | |
| **… in a rural area** | | | | | | | | | | | | | | |
| Females | | 206 (69.8%) | | | | | 42 (14.2%) | | | | | 47 (15.9%) | *295* | 0.881 ^ii^ |
| Males | | 164 (71.3%) | | | | | 33 (14.3%) | | | | | 33 (14.3%) | *230* |  |
| **… in an urban area** | | | | | | | | | | | | | | |
| Females | | 230 (77.7%) | | | | | 31 (10.5%) | | | | | 35 (11.8%) | *296* | 0.188 ^ii^ |
| Males | | 192 (83.5%) | | | | | 21 (9.1%) | | | | | 17 (7.4%) | *230* |  |

*GP* General Practitioner, *Exp* Experience

^ii^ Chi² Test

### **Supplementary Table S5b**: Gender-related analyses regarding interest in general practice, feeling of preparedness to work as a GP, specialist qualification, and personal preferences regarding various professional profiles and working circumstances (part C of the questionnaire) – RESIDENTS

| **RESIDENTS** | | **Only general practice** | | | | **General practice or other specialty** | | | **Only other specialty** | | | **Don’t know** | ***n*** | **p-value** |
| --- | --- | --- | --- | --- | --- | --- | --- | --- | --- | --- | --- | --- | --- | --- |
| **C1. Currently planned specialty choice** | | | | | | | | | | | | | | |
| Females | | 10 (16.9%) | | | | 30 (50.8%) | | | 17 (28.8%) | | | 2 (3.4%) | *59* | 0.063 ^ii^ |
| Males | | 2 (4.5%) | | | | 21 (47.7%) | | | 21 (47.7%) | | | 0 (0.0%) | *44* |  |
|  | | **Yes, surely** | | **Rather yes** | | | **Neutral** | | | **Rather no** | | **Not at all** | ***n*** | **p-value** |
| **C2. Interest in general practice as a specialty for the future professional life** | | | | | | | | | | | | | | |
| Females | | 21 (36.2%) | | 13 (22.4%) | | | 15 (25.9%) | | | 7 (12.1%) | | 2 (3.4%) | *58* | 0.063 ^ii^ |
| Males | | 11 (25.0%) | | 12 (27.3%) | | | 5 (11.4%) | | | 10 (22.7%) | | 6 (13.6%) | *44* |  |
|  | **Very good** | | **Good** | | **Neutral** | | | **Bad** | | | **Very bad** | **No exp.** | ***n*** | **p-value** |
| **C3a. Quality of the practical experiences in general practice during medical school** | | | | | | | | | | | | | | |
| Females | 24 (40.7%) | | 16 (27.1%) | | 13 (22.0%) | | | 3 (5.1%) | | | 2 (3.4%) | 1 (1.7%) | *59* | 0.267 ^ii^ |
| Males | 12 (27.3%) | | 22 (50.0%) | | 8 (18.2%) | | | 1 (2.3%) | | | 1 (2.3%) | 0 (0.0%) | *44* |  |
| **C3b. Quality of the theoretical experiences in general practice during medical school** | | | | | | | | | | | | | | |
| Females | 1 (1.7%) | | 13 (22.0%) | | 22 (37.3%) | | | 16 (27.1%) | | | 6 (10.2%) | 1 (1.7%) | *59* | 0.866 ^ii^ |
| Males | 3 (6.8%) | | 9 (20.5%) | | 15 (34.1%) | | | 12 (27.3%) | | | 4 (9.1%) | 1 (2.3%) | *44* |  |
|  | | **Yes, surely** | | **Rather yes** | | | **Neutral** | | | **Rather no** | | **Not at all** | ***n*** | **p-value** |
| **C4a. I feel well prepared to work as a GP by medical school** | | | | | | | | | | | | | | |
| Females | | 2 (3.4%) | | 1 (1.7%) | | | 15 (25.4%) | | | 31 (52.5%) | | 10 (16.9%) | *59* | 0.272 ^ii^ |
| Males | | 0 (0.0%) | | 4 (9.1%) | | | 9 (20.5%) | | | 21 (47.7%) | | 10 (22.7%) | *44* |  |
| **C4b. I feel well prepared to work as a GP by the general practice training** | | | | | | | | | | | | | | |
| Females | | 7 (12.3%) | | 24 (42.1%) | | | 12 (21.1%) | | | 11 (19.3%) | | 3 (5.3%) | *57* | 0.825 ^ii^ |
| Males | | 5 (11.6%) | | 15 (34.9%) | | | 12 (27.9%) | | | 7 (16.3%) | | 4 (9.3%) | *43* |  |
| **C5. A specialist qualification would increase the attractiveness of general practice** | | | | | | | | | | | | | | |
| Females | | 18 (30.5%) | | 17 (28.8%) | | | 9 (15.3%) | | | 11 (18.6%) | | 4 (6.8%) | *59* | 0.369 ^ii^ |
| Males | | 7 (15.9%) | | 17 (38.6%) | | | 7 (15.9%) | | | 7 (15.9%) | | 6 (13.6%) | *44* |  |
|  | | **Yes, surely** | | **Rather yes** | | | **Neutral** | | | **Rather no** | | **Not at all** | ***n*** | **p-value** |
| **C6. A specialist qualification is more attractive to me than general practice, because …** | | | | | | | | | | | | | | |
| **… without any specialist qualification, I would be a ‘lesser’ physician** | | | | | | | | | | | | | | |
| Females | | 7 (11.9%) | | 11 (18.6%) | | | 11 (18.6%) | | | 14 (23.7%) | | 16 (27.1%) | *59* | 0.615 ^ii^ |
| Males | | 5 (11.6%) | | 5 (11.6%) | | | 6 (14.0%) | | | 16 (37.2%) | | 11 (25.6%) | *43* |  |
| **… I can get higher reputation as a specialist** | | | | | | | | | | | | | | |
| Females | | 8 (13.6%) | | 14 (23.7%) | | | 13 (22.0%) | | | 13 (22.0%) | | 11 (18.6%) | *59* | 0.978 ^ii^ |
| Males | | 5 (11.9%) | | 11 (26.2%) | | | 8 (19.0%) | | | 11 (26.2%) | | 7 (16.7%) | *42* |  |
| **… teamwork is easier as a specialist** | | | | | | | | | | | | | | |
| Females | | 13 (22.0%) | | 15 (25.4%) | | | 13 (22.0%) | | | 13 (22.0%) | | 5 (8.5%) | *59* | 0.230 ^ii^ |
| Males | | 5 (11.6%) | | 13 (30.2%) | | | 5 (11.6%) | | | 12 (27.9%) | | 8 (18.6%) | *43* |  |
| **… I have better possibilities to work in research** | | | | | | | | | | | | | | |
| Females | | 10 (16.9%) | | 15 (25.4%) | | | 11 (18.6%) | | | 13 (22.0%) | | 10 (16.9%) | *59* | 0.693 ^ii^ |
| Males | | 5 (11.6%) | | 12 (27.9%) | | | 5 (11.6%) | | | 10 (23.3%) | | 11 (25.6%) | *43* |  |
| **… I have a higher income** | | | | | | | | | | | | | | |
| Females | | 19 (32.8%) | | 16 (27.6%) | | | 13 (22.4%) | | | 5 (8.6%) | | 5 (8.6%) | *58* | 0.108 ^ii^ |
| Males | | 11 (25.6%) | | 12 (27.9%) | | | 5 (11.6%) | | | 12 (27.9%) | | 3 (7.0%) | *43* |  |
| **… there are clearer content-related demarcations of the field** | | | | | | | | | | | | | | |
| Females | | 15 (25.4%) | | 19 (32.2%) | | | 14 (23.7%) | | | 6 (10.2%) | | 5 (8.5%) | *59* | 0.377 ^ii^ |
| Males | | 10 (23.3%) | | 15 (34.9%) | | | 12 (27.9%) | | | 6 (14.0%) | | 0 (0.0%) | *43* |  |
| **… I am facing more challenging medical topics** | | | | | | | | | | | | | | |
| Females | | 11 (18.6%) | | 5 (8.5%) | | | 21 (35.6%) | | | 15 (25.4%) | | 7 (11.9%) | *59* | 0.084 ^ii^ |
| Males | | 4 (9.3%) | | 12 (27.9%) | | | 12 (27.9%) | | | 12 (27.9%) | | 3 (7.0%) | *43* |  |
| **… I have better opportunities of career** | | | | | | | | | | | | | | |
| Females | | 15 (25.4%) | | 23 (39.0%) | | | 8 (13.6%) | | | 9 (15.3%) | | 4 (6.8%) | *59* | 0.336 ^ii^ |
| Males | | 10 (23.3%) | | 10 (23.3%) | | | 11 (25.6%) | | | 7 (16.3%) | | 5 (11.6%) | *43* |  |
| **… I can extend my knowledge in a more targeted way** | | | | | | | | | | | | | | |
| Females | | 21 (35.6%) | | 16 (27.1%) | | | 13 (22.0%) | | | 7 (11.9%) | | 2 (3.4%) | *59* | 0.198 ^ii^ |
| Males | | 11 (25.6%) | | 21 (48.8%) | | | 7 (16.3%) | | | 2 (4.7%) | | 2 (4.7%) | *43* |  |
|  | | **Yes** | | | | | **No** | | | | | **Don’t know** | ***n*** | **p-value** |
| **C7. I would preferably work …** | | | | | | | | | | | | | | |
| **… as an independent physician in a group office** | | | | | | | | | | | | | | |
| Females | | 51 (86.4%) | | | | | 1 (1.7%) | | | | | 7 (11.9%) | *59* | **0.031** ^ii^ |
| Males | | 28 (66.7%) | | | | | 5 (11.9%) | | | | | 9 (21.4%) | *42* |  |
| **… as an independent physician in a single-handed office** | | | | | | | | | | | | | | |
| Females | | 15 (25.4%) | | | | | 32 (54.2%) | | | | | 12 (20.3%) | *59* | 0.207 ^ii^ |
| Males | | 18 (41.9%) | | | | | 19 (44.2%) | | | | | 6 (14.0%) | *43* |  |
| **… as a physician in the hospital setting** | | | | | | | | | | | | | | |
| Females | | 27 (45.8%) | | | | | 21 (35.6%) | | | | | 11 (18.6%) | *59* | 0.389 ^ii^ |
| Males | | 26 (59.1%) | | | | | 11 (25.0%) | | | | | 7 (15.9%) | *44* |  |
| **… as an employed physician in another physician’s office** | | | | | | | | | | | | | | |
| Females | | 37 (62.7%) | | | | | 8 (13.6%) | | | | | 14 (23.7%) | *59* | **0.016** ^ii^ |
| Males | | 18 (42.9%) | | | | | 16 (38.1%) | | | | | 8 (19.0%) | *42* |  |
| **… as a GP in a multiprofessional team (e.g. Primary Healthcare Center)** | | | | | | | | | | | | | | |
| Females | | 42 (71.2%) | | | | | 9 (15.3%) | | | | | 8 (13.6%) | *59* | 0.068 ^ii^ |
| Males | | 24 (55.8%) | | | | | 15 (34.9%) | | | | | 4 (9.3%) | *43* |  |
| **… as a GP in the primary care setting** | | | | | | | | | | | | | | |
| Females | | 37 (62.7%) | | | | | 17 (28.8%) | | | | | 5 (8.5%) | *59* | 0.111 ^ii^ |
| Males | | 18 (41.9%) | | | | | 20 (46.5%) | | | | | 5 (11.6%) | *43* |  |
| **… as a ward physician in a hospital** | | | | | | | | | | | | | | |
| Females | | 5 (8.6%) | | | | | 40 (69.0%) | | | | | 13 (22.4%) | *58* | 0.560 ^ii^ |
| Males | | 4 (9.3%) | | | | | 33 (76.7%) | | | | | 6 (14.0%) | *43* |  |
| **… as a school physician** | | | | | | | | | | | | | | |
| Females | | 11 (19.0%) | | | | | 36 (62.1%) | | | | | 11 (19.0%) | *58* | 0.103 ^ii^ |
| Males | | 9 (20.9%) | | | | | 32 (74.4%) | | | | | 2 (4.7%) | *43* |  |
| **… as a public health officer** | | | | | | | | | | | | | | |
| Females | | 6 (10.2%) | | | | | 40 (67.8%) | | | | | 13 (22.0%) | *59* | 0.258 ^ii^ |
| Males | | 9 (22.0%) | | | | | 25 (61.0%) | | | | | 7 (17.1%) | *41* |  |
| **… as a physician with insurance companies or health insurances** | | | | | | | | | | | | | | |
| Females | | 4 (6.9%) | | | | | 46 (79.3%) | | | | | 8 (13.8%) | *58* | 0.315 ^ii^ |
| Males | | 6 (14.3%) | | | | | 33 (78.6%) | | | | | 3 (7.1%) | *42* |  |
| **… in a scientific career** | | | | | | | | | | | | | | |
| Females | | 10 (16.9%) | | | | | 39 (66.1%) | | | | | 10 (16.9%) | *59* | 0.610 ^ii^ |
| Males | | 10 (23.3%) | | | | | 28 (65.1%) | | | | | 5 (11.6%) | *43* |  |
| **… in a non-medical career (e.g. pharmaceutical industry, economy)** | | | | | | | | | | | | | | |
| Females | | 7 (11.9%) | | | | | 40 (67.8%) | | | | | 12 (20.3%) | *59* | 0.445 ^ii^ |
| Males | | 9 (20.9%) | | | | | 27 (62.8%) | | | | | 7 (16.3%) | *43* |  |
| **C8. I would like to work …** | | | | | | | | | | | | | | |
| **… in a rural area** | | | | | | | | | | | | | | |
| Females | | 47 (82.5%) | | | | | 5 (8.8%) | | | | | 5 (8.8%) | *57* | 0.411 ^ii^ |
| Males | | 34 (79.1%) | | | | | 2 (4.7%) | | | | | 7 (16.3%) | *43* |  |
| **… in an urban area** | | | | | | | | | | | | | | |
| Females | | 37 (62.7%) | | | | | 10 (16.9%) | | | | | 12 (20.3%) | *59* | 0.848 ^ii^ |
| Males | | 29 (65.9%) | | | | | 8 (18.2%) | | | | | 7 (15.9%) | *44* |  |

*GP* General Practitioner, *Exp* Experience

^ii^ Chi² Test

### **Supplementary Table S6a**: Age-related analyses regarding interest in general practice, feeling of preparedness to work as a GP, specialist qualification, and personal preferences regarding various professional profiles and working circumstances (part C of the questionnaire) – MEDICAL STUDENTS

| **MEDICAL STUDENTS** | | **Only general practice** | | | | **General practice or other specialty** | | | **Only other specialty** | | | **Don’t know** | ***n*** | **p-value** |
| --- | --- | --- | --- | --- | --- | --- | --- | --- | --- | --- | --- | --- | --- | --- |
| **C1. Currently planned specialty choice** | | | | | | | | | | | | | |  |
| < 23 years | | 4 (2.4%) | | | | 58 (35.2%) | | | 75 (45.5%) | | | 28 (17.0%) | *165* | **0.004** ^ii^ |
| ≥ 23 years | | 13 (3.7%) | | | | 171 (49.3%) | | | 132 (38.0%) | | | 31 (8.9%) | *347* |  |
|  | | **Yes, surely** | | **Rather yes** | | | **Neutral** | | | **Rather no** | | **Not at all** | ***n*** | **p-value** |
| **C2. Interest in general practice as a specialty for the future professional life** | | | | | | | | | | | | | |  |
| < 23 years | | 19 (11.5%) | | 34 (20.6%) | | | 50 (30.3%) | | | 46 (27.9%) | | 16 (9.7%) | *165* | 0.158 ^ii^ |
| ≥ 23 years | | 55 (15.9%) | | 96 (27.7%) | | | 92 (26.6%) | | | 73 (21.1%) | | 30 (8.7%) | *346* |  |
|  | **Very good** | | **Good** | | **Neutral** | | | **Bad** | | | **Very bad** | **No exp.** | ***n*** | **p-value** |
| **C3a. Quality of the practical experiences in general practice during medical school** | | | | | | | | | | | | | | |
| < 23 years | 17 (10.4%) | | 15 (9.1%) | | 8 (4.9%) | | | 1 (0.6%) | | | 0 (0.0%) | 123 (75.0%) | *164* | <**0.001** ^ii^ |
| ≥ 23 years | 89 (25.6%) | | 77 (22.2%) | | 36 (10.4%) | | | 12 (3.5%) | | | 2 (0.6%) | 131 (37.8%) | *347* |  |
| **C3b. Quality of the theoretical experiences in general practice during medical school** | | | | | | | | | | | | | | |
| < 23 years | 21 (12.7%) | | 71 (43.0%) | | 49 (29.7%) | | | 12 (7.3%) | | | 1 (0.6%) | 11 (6.7%) | *165* | **0.005** ^ii^ |
| ≥ 23 years | 33 (9.5%) | | 129 (37.2%) | | 122 (35.2%) | | | 42 (12.1%) | | | 14 (4.0%) | 7 (2.0%) | *347* |  |
|  | | **Yes, surely** | | **Rather yes** | | | **Neutral** | | | **Rather no** | | **Not at all** | ***n*** | **p-value** |
| **C4a. I feel well prepared to work as a GP by medical school** | | | | | | | | | | | | | | |
| < 23 years | | 4 (2.5%) | | 54 (33.3%) | | | 63 (38.9%) | | | 37 (22.8%) | | 4 (2.5%) | *162* | <**0.001** ^ii^ |
| ≥ 23 years | | 7 (2.0%) | | 61 (17.6%) | | | 129 (37.3%) | | | 124 (35.8%) | | 25 (7.2%) | *346* |  |
| **C5. A specialist qualification would increase the attractiveness of general practice** | | | | | | | | | | | | | | |
| < 23 years | | 22 (13.4%) | | 51 (31.1%) | | | 33 (20.1%) | | | 33 (20.1%) | | 25 (15.2%) | *164* | 0.532 ^ii^ |
| ≥ 23 years | | 62 (17.9%) | | 104 (30.0%) | | | 53 (15.3%) | | | 69 (19.9%) | | 59 (17.0%) | *347* |  |
|  | | **Yes, surely** | | **Rather yes** | | | **Neutral** | | | **Rather no** | | **Not at all** | ***n*** | **p-value** |
| **C6. A specialist qualification is more attractive to me than general practice, because …** | | | | | | | | | | | | | | |
| **… without any specialist qualification, I would be a ‘lesser’ physician** | | | | | | | | | | | | | | |
| < 23 years | | 9 (5.5%) | | 28 (17.1%) | | | 18 (11.0%) | | | 50 (30.5%) | | 59 (36.0%) | *164* | 0.757 ^ii^ |
| ≥ 23 years | | 18 (5.2%) | | 57 (16.4%) | | | 47 (13.5%) | | | 89 (25.6%) | | 136 (39.2%) | *347* |  |
| **… I can get higher reputation as a specialist** | | | | | | | | | | | | | | |
| < 23 years | | 21 (12.8%) | | 44 (26.8%) | | | 23 (14.0%) | | | 39 (23.8%) | | 37 (22.6%) | *164* | 0.202 ^ii^ |
| ≥ 23 years | | 42 (12.2%) | | 73 (21.2%) | | | 54 (15.7%) | | | 67 (19.4%) | | 109 (31.6%) | *345* |  |
| **… teamwork is easier as a specialist** | | | | | | | | | | | | | | |
| < 23 years | | 22 (13.4%) | | 45 (27.4%) | | | 37 (22.6%) | | | 40 (24.4%) | | 20 (12.2%) | *164* | 0.227 ^ii^ |
| ≥ 23 years | | 39 (11.3%) | | 99 (28.7%) | | | 68 (19.7%) | | | 70 (20.3%) | | 69 (20.0%) | *345* |  |
| **… I have better possibilities to work in research** | | | | | | | | | | | | | | |
| < 23 years | | 39 (23.6%) | | 52 (31.5%) | | | 27 (16.4%) | | | 28 (17.0%) | | 19 (11.5%) | *165* | 0.785 ^ii^ |
| ≥ 23 years | | 74 (21.4%) | | 109 (31.6%) | | | 52 (15.1%) | | | 56 (16.2%) | | 54 (15.7%) | *345* |  |
| **… I have a higher income** | | | | | | | | | | | | | | |
| < 23 years | | 39 (23.6%) | | 47 (28.5%) | | | 33 (20.0%) | | | 26 (15.8%) | | 20 (12.1%) | *165* | 0.920 ^ii^ |
| ≥ 23 years | | 85 (24.7%) | | 109 (31.7%) | | | 64 (18.6%) | | | 50 (14.5%) | | 36 (10.5%) | *344* |  |
| **… there are clearer content-related demarcations of the field** | | | | | | | | | | | | | | |
| < 23 years | | 51 (30.9%) | | 47 (28.5%) | | | 27 (16.4%) | | | 28 (17.0%) | | 12 (7.3%) | *165* | 0.142 ^ii^ |
| ≥ 23 years | | 74 (21.4%) | | 119 (34.4%) | | | 65 (18.8%) | | | 53 (15.3%) | | 35 (10.1%) | *346* |  |
| **… I am facing more challenging medical topics** | | | | | | | | | | | | | | |
| < 23 years | | 51 (31.1%) | | 40 (24.4%) | | | 26 (15.9%) | | | 35 (21.3%) | | 12 (7.3%) | *164* | **0.001** ^ii^ |
| ≥ 23 years | | 62 (17.9%) | | 67 (19.3%) | | | 82 (23.6%) | | | 88 (25.4%) | | 48 (13.8%) | *347* |  |
| **… I have better opportunities of career** | | | | | | | | | | | | | | |
| < 23 years | | 52 (31.5%) | | 51 (30.9%) | | | 34 (20.6%) | | | 19 (11.5%) | | 9 (5.5%) | *165* | 0.059 ^ii^ |
| ≥ 23 years | | 82 (23.7%) | | 106 (30.6%) | | | 65 (18.8%) | | | 49 (14.2%) | | 44 (12.7%) | *346* |  |
| **… I can extend my knowledge in a more targeted way** | | | | | | | | | | | | | | |
| < 23 years | | 86 (52.1%) | | 45 (27.3%) | | | 21 (12.7%) | | | 10 (6.1%) | | 3 (1.8%) | *165* | 0.173 ^ii^ |
| ≥ 23 years | | 151 (43.5%) | | 100 (28.8%) | | | 49 (14.1%) | | | 27 (7.8%) | | 20 (5.8%) | *347* |  |
|  | | **Yes** | | | | | **No** | | | | | **Don’t know** | ***n*** | **p-value** |
| **C7. I would preferably work …** | | | | | | | | | | | | | | |
| **… as an independent physician in a group office** | | | | | | | | | | | | | | |
| < 23 years | | 96 (58.2%) | | | | | 34 (20.6%) | | | | | 35 (21.2%) | *165* | 0.114 ^ii^ |
| ≥ 23 years | | 234 (67.2%) | | | | | 61 (17.5%) | | | | | 53 (15.2%) | *348* |  |
| **… as an independent physician in a single-handed office** | | | | | | | | | | | | | | |
| < 23 years | | 86 (52.1%) | | | | | 50 (30.3%) | | | | | 29 (17.6%) | *165* | **0.007** ^ii^ |
| ≥ 23 years | | 139 (40.1%) | | | | | 155 (44.7%) | | | | | 53 (15.3%) | *347* |  |
| **… as a physician in the hospital setting** | | | | | | | | | | | | | | |
| < 23 years | | 121 (73.3%) | | | | | 21 (12.7%) | | | | | 23 (13.9%) | *165* | **0.019** ^ii^ |
| ≥ 23 years | | 229 (66.2%) | | | | | 80 (23.1%) | | | | | 37 (10.7%) | *346* |  |
| **… as an employed physician in another physician’s office** | | | | | | | | | | | | | | |
| < 23 years | | 43 (26.4%) | | | | | 91 (55.8%) | | | | | 29 (17.8%) | *163* | **0.033** ^ii^ |
| ≥ 23 years | | 128 (37.1%) | | | | | 153 (44.3%) | | | | | 64 (18.6%) | *345* |  |
| **… as a GP in a multiprofessional team (e.g. Primary Healthcare Center)** | | | | | | | | | | | | | | |
| < 23 years | | 49 (29.7%) | | | | | 70 (42.4%) | | | | | 46 (27.9%) | *165* | <**0.001** ^ii^ |
| ≥ 23 years | | 171 (49.4%) | | | | | 107 (30.9%) | | | | | 68 (19.7%) | *346* |  |
| **… as a GP in the primary care setting** | | | | | | | | | | | | | | |
| < 23 years | | 52 (31.5%) | | | | | 72 (43.6%) | | | | | 41 (24.8%) | *165* | 0.125 ^ii^ |
| ≥ 23 years | | 141 (40.9%) | | | | | 129 (37.4%) | | | | | 75 (21.7%) | *345* |  |
| **… as a ward physician in a hospital** | | | | | | | | | | | | | | |
| < 23 years | | 56 (33.9%) | | | | | 65 (39.4%) | | | | | 44 (26.7%) | *165* | <**0.001** ^ii^ |
| ≥ 23 years | | 54 (15.6%) | | | | | 229 (66.0%) | | | | | 64 (18.4%) | *347* |  |
| **… as a school physician** | | | | | | | | | | | | | | |
| < 23 years | | 18 (10.9%) | | | | | 136 (82.4%) | | | | | 11 (6.7%) | *165* | **0.044** ^ii^ |
| ≥ 23 years | | 45 (13.0%) | | | | | 255 (73.5%) | | | | | 47 (13.5%) | *347* |  |
| **… as a public health officer** | | | | | | | | | | | | | | |
| < 23 years | | 19 (11.6%) | | | | | 127 (77.4%) | | | | | 18 (11.0%) | *164* | 0.878 ^ii^ |
| ≥ 23 years | | 41 (11.9%) | | | | | 261 (75.7%) | | | | | 43 (12.5%) | *345* |  |
| **… as a physician with insurance companies or health insurances** | | | | | | | | | | | | | | |
| < 23 years | | 4 (2.4%) | | | | | 149 (90.3%) | | | | | 12 (7.3%) | *165* | 0.187 ^ii^ |
| ≥ 23 years | | 18 (5.2%) | | | | | 294 (84.7%) | | | | | 35 (10.1%) | *347* |  |
| **… in a scientific career** | | | | | | | | | | | | | | |
| < 23 years | | 63 (38.4%) | | | | | 74 (45.1%) | | | | | 27 (16.5%) | *164* | 0.062 ^ii^ |
| ≥ 23 years | | 98 (28.3%) | | | | | 189 (54.6%) | | | | | 59 (17.1%) | *346* |  |
| **… in a non-medical career (e.g. pharmaceutical industry, economy)** | | | | | | | | | | | | | | |
| < 23 years | | 11 (6.7%) | | | | | 137 (83.0%) | | | | | 17 (10.3%) | *165* | 0.329 ^ii^ |
| ≥ 23 years | | 32 (9.3%) | | | | | 266 (77.3%) | | | | | 46 (13.4%) | *344* |  |
| **C8. I would like to work …** | | | | | | | | | | | | | | |
| **… in a rural area** | | | | | | | | | | | | | | |
| < 23 years | | 110 (67.1%) | | | | | 30 (18.3%) | | | | | 24 (14.6%) | *164* | 0.167 ^ii^ |
| ≥ 23 years | | 252 (72.4%) | | | | | 42 (12.1%) | | | | | 54 (15.5%) | *348* |  |
| **… in an urban area** | | | | | | | | | | | | | | |
| < 23 years | | 133 (80.6%) | | | | | 13 (7.9%) | | | | | 19 (11.5%) | *165* | 0.513 ^ii^ |
| ≥ 23 years | | 278 (79.9%) | | | | | 37 (10.6%) | | | | | 33 (9.5%) | *348* |  |

*GP* General Practitioner, *Exp* Experience

^ii^ Chi² Test

### **Supplementary Table S6b**: Age-related analyses regarding interest in general practice, feeling of preparedness to work as a GP, specialist qualification, and personal preferences regarding various professional profiles and working circumstances (part C of the questionnaire) – RESIDENTS

| **RESIDENTS** | | **Only general practice** | | | | **General practice or other specialty** | | | **Only other specialty** | | | **Don’t know** | ***n*** | **p-value** |
| --- | --- | --- | --- | --- | --- | --- | --- | --- | --- | --- | --- | --- | --- | --- |
| **C1. Currently planned specialty choice** | | | | | | | | | | | | | |  |
| < 30 years | | 9 (14.1%) | | | | 38 (59.4%) | | | 15 (23.4%) | | | 2 (3.1%) | *64* | **0.003** ^ii^ |
| ≥ 30 years | | 3 (7.7%) | | | | 13 (33.3%) | | | 23 (59.0%) | | | 0 (0.0%) | *39* |  |
|  | | **Yes, surely** | | **Rather yes** | | | **Neutral** | | | **Rather no** | | **Not at all** | ***n*** | **p-value** |
| **C2. Interest in general practice as a specialty for the future professional life** | | | | | | | | | | | | | |  |
| < 30 years | | 23 (35.9%) | | 19 (29.7%) | | | 9 (14.1%) | | | 9 (14.1%) | | 4 (6.3%) | *64* | 0.138 ^ii^ |
| ≥ 30 years | | 9 (23.7%) | | 6 (15.8%) | | | 11 (28.9%) | | | 8 (21.1%) | | 4 (10.5%) | *38* |  |
|  | **Very good** | | **Good** | | **Neutral** | | | **Bad** | | | **Very bad** | **No exp.** | ***n*** | **p-value** |
| **C3a. Quality of the practical experiences in general practice during medical school** | | | | | | | | | | | | | | |
| < 30 years | 27 (42.2%) | | 23 (35.9%) | | 12 (18.8%) | | | 1 (1.6%) | | | 1 (1.6%) | 0 (0.0%) | *64* | 0.165 ^ii^ |
| ≥ 30 years | 9 (23.1%) | | 15 (38.5%) | | 9 (23.1%) | | | 3 (7.7%) | | | 2 (5.1%) | 1 (2.6%) | *39* |  |
| **C3b. Quality of the theoretical experiences in general practice during medical school** | | | | | | | | | | | | | | |
| < 30 years | 2 (3.1%) | | 16 (25.0%) | | 25 (39.1%) | | | 13 (20.3%) | | | 7 (10.9%) | 1 (1.6%) | *64* | 0.405 ^ii^ |
| ≥ 30 years | 2 (5.1%) | | 6 (15.4%) | | 12 (30.8%) | | | 15 (38.5%) | | | 3 (7.7%) | 1 (2.6%) | *39* |  |
|  | | **Yes, surely** | | **Rather yes** | | | **Neutral** | | | **Rather no** | | **Not at all** | ***n*** | **p-value** |
| **C4a. I feel well prepared to work as a GP by medical school** | | | | | | | | | | | | | | |
| < 30 years | | 2 (3.1%) | | 3 (4.7%) | | | 19 (29.7%) | | | 30 (46.9%) | | 10 (15.6%) | *64* | 0.209 ^ii^ |
| ≥ 30 years | | 0 (0.0%) | | 2 (5.1%) | | | 5 (12.8%) | | | 22 (56.4%) | | 10 (25.6%) | *39* |  |
| **C4b. I feel well prepared to work as a GP by the general practice training** | | | | | | | | | | | | | | |
| < 30 years | | 10 (16.1%) | | 29 (46.8%) | | | 13 (21.0%) | | | 8 (12.9%) | | 2 (3.2%) | *62* | **0.025** ^ii^ |
| ≥ 30 years | | 2 (5.3%) | | 10 (26.3%) | | | 11 (28.9%) | | | 10 (26.3%) | | 5 (13.2%) | *38* |  |
| **C5. A specialist qualification would increase the attractiveness of general practice** | | | | | | | | | | | | | | |
| < 30 years | | 15 (23.4%) | | 19 (29.7%) | | | 9 (14.1%) | | | 13 (20.3%) | | 8 (12.5%) | *64* | 0.561 ^ii^ |
| ≥ 30 years | | 10 (25.6%) | | 15 (38.5%) | | | 7 (17.9%) | | | 5 (12.8%) | | 2 (5.1%) | *39* |  |
|  | | **Yes, surely** | | **Rather yes** | | | **Neutral** | | | **Rather no** | | **Not at all** | ***n*** | **p-value** |
| **C6. A specialist qualification is more attractive to me than general practice, because …** | | | | | | | | | | | | | | |
| **… without any specialist qualification, I would be a ‘lesser’ physician** | | | | | | | | | | | | | | |
| < 30 years | | 6 (9.5%) | | 9 (14.3%) | | | 11 (17.5%) | | | 21 (33.3%) | | 16 (25.4%) | *63* | 0.753 ^ii^ |
| ≥ 30 years | | 6 (15.4%) | | 7 (17.9%) | | | 6 (15.4%) | | | 9 (23.1%) | | 11 (28.2%) | *39* |  |
| **… I can get higher reputation as a specialist** | | | | | | | | | | | | | | |
| < 30 years | | 8 (12.7%) | | 14 (22.2%) | | | 15 (23.8%) | | | 16 (25.4%) | | 10 (15.9%) | *63* | 0.788 ^ii^ |
| ≥ 30 years | | 5 (13.2%) | | 11 (28.9%) | | | 6 (15.8%) | | | 8 (21.1%) | | 8 (21.1%) | *38* |  |
| **… teamwork is easier as a specialist** | | | | | | | | | | | | | | |
| < 30 years | | 11 (17.5%) | | 18 (28.6%) | | | 10 (15.9%) | | | 19 (30.2%) | | 5 (7.9%) | *63* | 0.239 ^ii^ |
| ≥ 30 years | | 7 (17.9%) | | 10 (25.6%) | | | 8 (20.5%) | | | 6 (15.4%) | | 8 (20.5%) | *39* |  |
| **… I have better possibilities to work in research** | | | | | | | | | | | | | | |
| < 30 years | | 12 (19.0%) | | 17 (27.0%) | | | 11 (17.5%) | | | 13 (20.6%) | | 10 (15.9%) | *63* | 0.342 ^ii^ |
| ≥ 30 years | | 3 (7.7%) | | 10 (25.6%) | | | 5 (12.8%) | | | 10 (25.6%) | | 11 (28.2%) | *39* |  |
| **… I have a higher income** | | | | | | | | | | | | | | |
| < 30 years | | 20 (32.3%) | | 19 (30.6%) | | | 12 (19.4%) | | | 7 (11.3%) | | 4 (6.5%) | *62* | 0.351 ^ii^ |
| ≥ 30 years | | 10 (25.6%) | | 9 (23.1%) | | | 6 (15.4%) | | | 10 (25.6%) | | 4 (10.3%) | *39* |  |
| **… there are clearer content-related demarcations of the field** | | | | | | | | | | | | | | |
| < 30 years | | 19 (30.2%) | | 20 (31.7%) | | | 15 (23.8%) | | | 6 (9.5%) | | 3 (4.8%) | *63* | 0.531 ^ii^ |
| ≥ 30 years | | 6 (15.4%) | | 14 (35.9%) | | | 11 (28.2%) | | | 6 (15.4%) | | 2 (5.1%) | *39* |  |
| **… I am facing more challenging medical topics** | | | | | | | | | | | | | | |
| < 30 years | | 11 (17.5%) | | 10 (15.9%) | | | 23 (36.5%) | | | 14 (22.2%) | | 5 (7.9%) | *63* | 0.478 ^ii^ |
| ≥ 30 years | | 4 (10.3%) | | 7 (17.9%) | | | 10 (25.6%) | | | 13 (33.3%) | | 5 (12.8%) | *39* |  |
| **… I have better opportunities of career** | | | | | | | | | | | | | | |
| < 30 years | | 17 (27.0%) | | 23 (36.5%) | | | 13 (20.6%) | | | 7 (11.1%) | | 3 (4.8%) | *63* | 0.140 ^ii^ |
| ≥ 30 years | | 8 (20.5%) | | 10 (25.6%) | | | 6 (15.4%) | | | 9 (23.1%) | | 6 (15.4%) | *39* |  |
| **… I can extend my knowledge in a more targeted way** | | | | | | | | | | | | | | |
| < 30 years | | 24 (38.1%) | | 19 (30.2%) | | | 14 (22.2%) | | | 6 (9.5%) | | 0 (0.0%) | *63* | **0.024** ^ii^ |
| ≥ 30 years | | 8 (20.5%) | | 18 (46.2%) | | | 6 (15.4%) | | | 3 (7.7%) | | 4 (10.3%) | *39* |  |
|  | | **Yes** | | | | | **No** | | | | | **Don’t know** | ***n*** | **p-value** |
| **C7. I would preferably work …** | | | | | | | | | | | | | | |
| **… as an independent physician in a group office** | | | | | | | | | | | | | | |
| < 30 years | | 52 (81.3%) | | | | | 4 (6.3%) | | | | | 8 (12.5%) | *64* | 0.481 ^ii^ |
| ≥ 30 years | | 27 (73.0%) | | | | | 2 (5.4%) | | | | | 8 (21.6%) | *37* |  |
| **… as an independent physician in a single-handed office** | | | | | | | | | | | | | | |
| < 30 years | | 24 (37.5%) | | | | | 30 (46.9%) | | | | | 10 (15.6%) | *64* | 0.343 ^ii^ |
| ≥ 30 years | | 9 (23.7%) | | | | | 21 (55.3%) | | | | | 8 (21.1%) | *38* |  |
| **… as a physician in the hospital setting** | | | | | | | | | | | | | | |
| < 30 years | | 31 (48.4%) | | | | | 23 (35.9%) | | | | | 10 (15.6%) | *64* | 0.383 ^ii^ |
| ≥ 30 years | | 22 (56.4%) | | | | | 9 (23.1%) | | | | | 8 (20.5%) | *39* |  |
| **… as an employed physician in another physician’s office** | | | | | | | | | | | | | | |
| < 30 years | | 37 (57.8%) | | | | | 15 (23.4%) | | | | | 12 (18.8%) | *64* | 0.575 ^ii^ |
| ≥ 30 years | | 18 (48.6%) | | | | | 9 (24.3%) | | | | | 10 (27.0%) | *37* |  |
| **… as a GP in a multiprofessional team (e.g. Primary Healthcare Center)** | | | | | | | | | | | | | | |
| < 30 years | | 42 (65.6%) | | | | | 15 (23.4%) | | | | | 7 (10.9%) | *64* | 0.940 ^ii^ |
| ≥ 30 years | | 24 (63.2%) | | | | | 9 (23.7%) | | | | | 5 (13.2%) | *38* |  |
| **… as a GP in the primary care setting** | | | | | | | | | | | | | | |
| < 30 years | | 38 (59.4%) | | | | | 22 (34.4%) | | | | | 4 (6.3%) | *64* | 0.189 ^ii^ |
| ≥ 30 years | | 17 (44.7%) | | | | | 15 (39.5%) | | | | | 6 (15.8%) | *38* |  |
| **… as a ward physician in a hospital** | | | | | | | | | | | | | | |
| < 30 years | | 6 (9.5%) | | | | | 47 (74.6%) | | | | | 10 (15.9%) | *63* | 0.617 ^ii^ |
| ≥ 30 years | | 3 (7.9%) | | | | | 26 (68.4%) | | | | | 9 (23.7%) | *38* |  |
| **… as a school physician** | | | | | | | | | | | | | | |
| < 30 years | | 12 (19.0%) | | | | | 42 (66.7%) | | | | | 9 (14.3%) | *63* | 0.852 ^ii^ |
| ≥ 30 years | | 8 (21.1%) | | | | | 26 (68.4%) | | | | | 4 (10.5%) | *38* |  |
| **… as a public health officer** | | | | | | | | | | | | | | |
| < 30 years | | 9 (14.3%) | | | | | 43 (68.3%) | | | | | 11 (17.5%) | *63* | 0.643 ^ii^ |
| ≥ 30 years | | 6 (16.2%) | | | | | 22 (59.5%) | | | | | 9 (24.3%) | *37* |  |
| **… as a physician with insurance companies or health insurances** | | | | | | | | | | | | | | |
| < 30 years | | 5 (7.9%) | | | | | 53 (84.1%) | | | | | 5 (7.9%) | *63* | 0.254 ^ii^ |
| ≥ 30 years | | 5 (13.5%) | | | | | 26 (70.3%) | | | | | 6 (16.2%) | *37* |  |
| **… in a scientific career** | | | | | | | | | | | | | | |
| < 30 years | | 14 (21.9%) | | | | | 42 (65.6%) | | | | | 8 (12.5%) | *64* | 0.601 ^ii^ |
| ≥ 30 years | | 6 (15.8%) | | | | | 25 (65.8%) | | | | | 7 (18.4%) | *38* |  |
| **… in a non-medical career (e.g. pharmaceutical industry, economy)** | | | | | | | | | | | | | | |
| < 30 years | | 8 (12.5%) | | | | | 44 (68.8%) | | | | | 12 (18.8%) | *64* | 0.507 ^ii^ |
| ≥ 30 years | | 8 (21.1%) | | | | | 23 (60.5%) | | | | | 7 (18.4%) | *38* |  |
| **C8. I would like to work …** | | | | | | | | | | | | | | |
| **… in a rural area** | | | | | | | | | | | | | | |
| < 30 years | | 53 (85.5%) | | | | | 3 (4.8%) | | | | | 6 (9.7%) | *62* | 0.328 ^ii^ |
| ≥ 30 years | | 28 (73.7%) | | | | | 4 (10.5%) | | | | | 6 (15.8%) | *38* |  |
| **… in an urban area** | | | | | | | | | | | | | | |
| < 30 years | | 40 (62.5%) | | | | | 12 (18.8%) | | | | | 12 (18.8%) | *64* | 0.891 ^ii^ |
| ≥ 30 years | | 26 (66.7%) | | | | | 6 (15.4%) | | | | | 7 (17.9%) | *39* |  |

*GP* General Practitioner, *Exp* Experience

^ii^ Chi² Test

### **Supplementary Table S7a**: Nationality-related analyses regarding interest in general practice, feeling of preparedness to work as a GP, specialist qualification, and personal preferences regarding various professional profiles and working circumstances (part C of the questionnaire) – MEDICAL STUDENTS

| **MEDICAL STUDENTS** | | **Only general practice** | | | | **General practice or other specialty** | | | **Only other specialty** | | | **Don’t know** | ***n*** | **p-value** |
| --- | --- | --- | --- | --- | --- | --- | --- | --- | --- | --- | --- | --- | --- | --- |
| **C1. Currently planned specialty choice** | | | | | | | | | | | | | |  |
| Austria | | 10 (3.5%) | | | | 133 (47.0%) | | | 112 (39.6%) | | | 28 (9.9%) | *283* | 0.693 ^ii^ |
| Germany | | 4 (4.1%) | | | | 45 (45.9%) | | | 36 (36.7%) | | | 13 (13.3%) | *98* |  |
| South Tyrol | | 3 (2.4%) | | | | 49 (39.5%) | | | 54 (43.5%) | | | 18 (14.5%) | *124* |  |
| Others ^§^ | | 0 (0.0%) | | | | 6 (31.6%) | | | 10 (52.6%) | | | 3 (15.8%) | *19* |  |
|  | | **Yes, surely** | | **Rather yes** | | | **Neutral** | | | **Rather no** | | **Not at all** | ***n*** | **p-value** |
| **C2. Interest in general practice as a specialty for the future professional life** | | | | | | | | | | | | | |  |
| Austria | | 42 (14.8%) | | 78 (27.6%) | | | 66 (23.3%) | | | 72 (25.4%) | | 25 (8.8%) | *283* | 0.209 ^ii^ |
| Germany | | 15 (15.3%) | | 26 (26.5%) | | | 29 (29.6%) | | | 18 (18.4%) | | 10 (10.2%) | *98* |  |
| South Tyrol | | 17 (13.8%) | | 22 (17.9%) | | | 47 (38.2%) | | | 26 (21.1%) | | 11 (8.9%) | *123* |  |
| Others ^§^ | | 1 (5.3%) | | 7 (36.8%) | | | 4 (21.1%) | | | 4 (21.1%) | | 3 (15.8%) | *19* |  |
|  | **Very good** | | **Good** | | **Neutral** | | | **Bad** | | | **Very bad** | **No exp.** | ***n*** | **p-value** |
| **C3a. Quality of the practical experiences in general practice during medical school** | | | | | | | | | | | | | | |
| Austria | 62 (21.8%) | | 50 (17.6%) | | 26 (9.2%) | | | 10 (3.5%) | | | 0 (0.0%) | 136 (47.9%) | *284* | 0.720 ^ii^ |
| Germany | 20 (20.6%) | | 16 (16.5%) | | 5 (5.2%) | | | 2 (2.1%) | | | 1 (1.0%) | 53 (54.6%) | *97* |  |
| South Tyrol | 24 (19.5%) | | 22 (17.9%) | | 11 (8.9%) | | | 1 (0.8%) | | | 1 (0.8%) | 64 (52.0%) | *123* |  |
| Others ^§^ | 2 (10.5%) | | 5 (26.3%) | | 3 (15.8%) | | | 0 (0.0%) | | | 0 (0.0%) | 9 (47.4%) | *19* |  |
| **C3b. Quality of the theoretical experiences in general practice during medical school** | | | | | | | | | | | | | | |
| Austria | 30 (10.6%) | | 100 (35.2%) | | 97 (34.2%) | | | 36 (12.7%) | | | 10 (3.5%) | 11 (3.9%) | *284* | 0.762 ^ii^ |
| Germany | 9 (9.2%) | | 41 (41.8%) | | 35 (35.7%) | | | 9 (9.2%) | | | 1 (1.0%) | 3 (3.1%) | *98* |  |
| South Tyrol | 14 (11.4%) | | 55 (44.7%) | | 32 (26.0%) | | | 12 (9.8%) | | | 4 (3.3%) | 6 (4.9%) | *123* |  |
| Others ^§^ | 3 (15.8%) | | 7 (36.8%) | | 8 (42.1%) | | | 1 (5.3%) | | | 0 (0.0%) | 0 (0.0%) | *19* |  |
|  | | **Yes, surely** | | **Rather yes** | | | **Neutral** | | | **Rather no** | | **Not at all** | ***n*** | **p-value** |
| **C4a. I feel well prepared to work as a GP by medical school** | | | | | | | | | | | | | | |
| Austria | | 6 (2.1%) | | 60 (21.3%) | | | 106 (37.6%) | | | 92 (32.6%) | | 18 (6.4%) | *282* | 0.482 ^ii^ |
| Germany | | 2 (2.1%) | | 19 (19.8%) | | | 40 (41.7%) | | | 33 (34.4%) | | 2 (2.1%) | *96* |  |
| South Tyrol | | 3 (2.4%) | | 32 (26.0%) | | | 48 (39.0%) | | | 31 (25.2%) | | 9 (7.3%) | *123* |  |
| Others ^§^ | | 0 (0.0%) | | 8 (42.1%) | | | 6 (31.6%) | | | 5 (26.3%) | | 0 (0.0%) | *19* |  |
| **C5. A specialist qualification would increase the attractiveness of general practice** | | | | | | | | | | | | | | |
| Austria | | 48 (16.9%) | | 76 (26.8%) | | | 45 (15.8%) | | | 59 (20.8%) | | 56 (19.7%) | *284* | 0.443 ^ii^ |
| Germany | | 15 (15.6%) | | 36 (17.5%) | | | 16 (16.7%) | | | 15 (15.6%) | | 14 (14.6%) | *96* |  |
| South Tyrol | | 17 (13.7%) | | 42 (33.9%) | | | 23 (18.5%) | | | 25 (20.2%) | | 17 (13.7%) | *124* |  |
| Others ^§^ | | 6 (31.6%) | | 5 (26.3%) | | | 3 (15.8%) | | | 4 (21.1%) | | 1 (5.3%) | *19* |  |
|  | | **Yes, surely** | | **Rather yes** | | | **Neutral** | | | **Rather no** | | **Not at all** | ***n*** | **p-value** |
| **C6. A specialist qualification is more attractive to me than general practice, because …** | | | | | | | | | | | | | | |
| **… without any specialist qualification, I would be a ‘lesser’ physician** | | | | | | | | | | | | | | |
| Austria | | 10 (3.5%) | | 52 (18.4%) | | | 35 (12.4%) | | | 64 (22.6%) | | 122 (43.1%) | *283* | 0.086 ^ii^ |
| Germany | | 8 (8.2%) | | 16 (16.5%) | | | 13 (13.4%) | | | 31 (32.0%) | | 29 (29.9%) | *97* |  |
| South Tyrol | | 8 (6.5%) | | 18 (14.6%) | | | 14 (11.4%) | | | 38 (30.9%) | | 45 (36.6%) | *123* |  |
| Others ^§^ | | 2 (10.5%) | | 2 (10.5%) | | | 5 (26.3%) | | | 7 (36.8%) | | 3 (15.8%) | *19* |  |
| **… I can get higher reputation as a specialist** | | | | | | | | | | | | | | |
| Austria | | 34 (12.1%) | | 64 (22.7%) | | | 42 (14.9%) | | | 55 (19.5%) | | 82 (30.9%) | *282* | 0.766 ^ii^ |
| Germany | | 13 (13.3%) | | 27 (27.6%) | | | 16 (16.3%) | | | 20 (20.4%) | | 22 (22.4%) | *98* |  |
| South Tyrol | | 15 (12.3%) | | 23 (18.9%) | | | 19 (15.6%) | | | 30 (24.6%) | | 35 (28.7%) | *122* |  |
| Others ^§^ | | 4 (23.5%) | | 5 (29.4%) | | | 3 (17.6%) | | | 2 (11.8%) | | 3 (17.6%) | *17* |  |
| **… teamwork is easier as a specialist** | | | | | | | | | | | | | | |
| Austria | | 34 (12.1%) | | 69 (24.5%) | | | 57 (20.2%) | | | 68 (24.1%) | | 54 (19.1%) | *282* | **0.003** ^ii^ |
| Germany | | 3 (3.1%) | | 26 (26.8%) | | | 26 (26.8%) | | | 25 (25.8%) | | 17 (17.5%) | *97* |  |
| South Tyrol | | 24 (19.7%) | | 44 (36.1%) | | | 17 (13.9%) | | | 20 (16.4%) | | 17 (13.9%) | *122* |  |
| Others ^§^ | | 2 (10.5%) | | 6 (31.6%) | | | 7 (36.8%) | | | 1 (5.3%) | | 3 (15.8%) | *19* |  |
| **… I have better possibilities to work in research** | | | | | | | | | | | | | | |
| Austria | | 65 (23.0%) | | 81 (28.6%) | | | 46 (16.3%) | | | 44 (15.5%) | | 47 (16.6%) | *283* | 0.170 ^ii^ |
| Germany | | 15 (15.3%) | | 38 (38.8%) | | | 17 (17.3%) | | | 14 (14.3%) | | 14 (14.3%) | *98* |  |
| South Tyrol | | 29 (23.8%) | | 39 (32.0%) | | | 16 (13.1%) | | | 27 (22.1%) | | 11 (9.0%) | *122* |  |
| Others ^§^ | | 7 (38.9%) | | 6 (33.3%) | | | 1 (5.6%) | | | 1 (5.6%) | | 3 (16.7%) | *18* |  |
| **… I have a higher income** | | | | | | | | | | | | | | |
| Austria | | 78 (27.8%) | | 81 (28.8%) | | | 44 (15.7%) | | | 43 (15.3%) | | 35 (12.5%) | *281* | 0.218 ^ii^ |
| Germany | | 20 (20.4%) | | 36 (36.7%) | | | 22 (22.4%) | | | 10 (10.2%) | | 10 (10.2%) | *98* |  |
| South Tyrol | | 24 (19.7%) | | 37 (30.3%) | | | 26 (21.3%) | | | 24 (19.7%) | | 11 (9.0%) | *122* |  |
| Others ^§^ | | 5 (26.3%) | | 6 (31.6%) | | | 6 (31.6%) | | | 2 (10.5%) | | 0 (0.0%) | *19* |  |
| **… there are clearer content-related demarcations of the field** | | | | | | | | | | | | | | |
| Austria | | 72 (25.4%) | | 92 (32.5%) | | | 50 (17.7%) | | | 39 (13.8%) | | 30 (10.6%) | *283* | 0.117 ^ii^ |
| Germany | | 15 (15.5%) | | 36 (37.1%) | | | 17 (17.5%) | | | 23 (23.7%) | | 6 (6.2%) | *97* |  |
| South Tyrol | | 33 (26.8%) | | 35 (28.5%) | | | 23 (18.7%) | | | 21 (17.1%) | | 11 (8.9%) | *123* |  |
| Others ^§^ | | 7 (36.8%) | | 7 (36.8%) | | | 5 (26.3%) | | | 0 (0.0%) | | 0 (0.0%) | *19* |  |
| **… I am facing more challenging medical topics** | | | | | | | | | | | | | | |
| Austria | | 61 (21.6%) | | 62 (21.9%) | | | 62 (21.9%) | | | 60 (21.2%) | | 38 (13.4%) | *283* | 0.483 ^ii^ |
| Germany | | 16 (16.3%) | | 23 (23.5%) | | | 23 (23.5%) | | | 26 (26.5%) | | 10 (10.2%) | *98* |  |
| South Tyrol | | 33 (27.0%) | | 20 (16.4%) | | | 20 (16.4%) | | | 34 (27.9%) | | 15 (12.3%) | *122* |  |
| Others ^§^ | | 5 (26.3%) | | 4 (21.1%) | | | 5 (26.3%) | | | 5 (26.3%) | | 0 (0.0%) | *19* |  |
| **… I have better opportunities of career** | | | | | | | | | | | | | | |
| Austria | | 83 (29.4%) | | 74 (26.2%) | | | 57 (20.2%) | | | 35 (12.4%) | | 33 (11.7%) | *282* | 0.324 ^ii^ |
| Germany | | 20 (20.4%) | | 38 (38.8%) | | | 18 (18.4%) | | | 12 (12.2%) | | 10 (10.2%) | *98* |  |
| South Tyrol | | 28 (22.8%) | | 46 (37.4%) | | | 19 (15.4%) | | | 19 (15.4%) | | 11 (8.9%) | *123* |  |
| Others ^§^ | | 5 (26.3%) | | 7 (36.8%) | | | 5 (26.3%) | | | 2 (10.5%) | | 0 (0.0%) | *19* |  |
| **… I can extend my knowledge in a more targeted way** | | | | | | | | | | | | | | |
| Austria | | 135 (47.7%) | | 78 (27.6%) | | | 33 (11.7%) | | | 22 (7.8%) | | 15 (5.3%) | *283* | 0.869 ^ii^ |
| Germany | | 42 (42.9%) | | 27 (27.6%) | | | 17 (17.3%) | | | 9 (9.2%) | | 3 (3.1%) | *98* |  |
| South Tyrol | | 54 (43.9%) | | 39 (31.7%) | | | 18 (14.6%) | | | 7 (5.7%) | | 5 (4.1%) | *123* |  |
| Others ^§^ | | 11 (57.9%) | | 4 (21.1%) | | | 3 (15.8%) | | | 1 (5.3%) | | 0 (0.0%) | *19* |  |
|  | | **Yes** | | | | | **No** | | | | | **Don’t know** | ***n*** | **p-value** |
| **C7. I would preferably work …** | | | | | | | | | | | | | | |
| **… as an independent physician in a group office** | | | | | | | | | | | | | | |
| Austria | | 181 (64.0%) | | | | | 51 (18.0%) | | | | | 51 (18.0%) | *283* | 0.986 ^ii^ |
| Germany | | 64 (65.3%) | | | | | 18 (18.4%) | | | | | 16 (16.3%) | *98* |  |
| South Tyrol | | 80 (64.5%) | | | | | 25 (20.2%) | | | | | 19 (15.3%) | *124* |  |
| Others ^§^ | | 11 (57.9%) | | | | | 4 (21.1%) | | | | | 4 (21.1%) | *19* |  |
| **… as an independent physician in a single-handed office** | | | | | | | | | | | | | | |
| Austria | | 145 (51.2%) | | | | | 90 (31.8%) | | | | | 48 (17.0%) | *283* | **0.003** ^ii^ |
| Germany | | 32 (32.7%) | | | | | 50 (51.0%) | | | | | 16 (16.3%) | *98* |  |
| South Tyrol | | 45 (36.3%) | | | | | 61 (49.2%) | | | | | 18 (14.5%) | *124* |  |
| Others ^§^ | | 9 (50.0%) | | | | | 5 (27.8%) | | | | | 4 (22.2%) | *18* |  |
| **… as a physician in the hospital setting** | | | | | | | | | | | | | | |
| Austria | | 192 (67.8%) | | | | | 59 (20.8%) | | | | | 32 (11.3%) | *283* | 0.103 ^ii^ |
| Germany | | 60 (61.2%) | | | | | 26 (26.5%) | | | | | 12 (12.2%) | *98* |  |
| South Tyrol | | 94 (77.0%) | | | | | 15 (12.3%) | | | | | 13 (10.7%) | *122* |  |
| Others ^§^ | | 12 (66.7%) | | | | | 2 (11.1%) | | | | | 4 (22.2%) | *18* |  |
| **… as an employed physician in another physician’s office** | | | | | | | | | | | | | | |
| Austria | | 87 (30.9%) | | | | | 149 (52.8%) | | | | | 46 (16.3%) | *282* | 0.304 ^ii^ |
| Germany | | 37 (37.8%) | | | | | 38 (38.8%) | | | | | 23 (23.5%) | *98* |  |
| South Tyrol | | 44 (36.4%) | | | | | 54 (44.6%) | | | | | 23 (19.0%) | *121* |  |
| Others ^§^ | | 5 (27.8%) | | | | | 9 (50.0%) | | | | | 4 (22.2%) | *18* |  |
| **… as a GP in a multiprofessional team (e.g. Primary Healthcare Center)** | | | | | | | | | | | | | | |
| Austria | | 116 (41.1%) | | | | | 107 (37.9%) | | | | | 59 (20.9%) | *282* | 0.608 ^ii^ |
| Germany | | 42 (42.9%) | | | | | 31 (31.6%) | | | | | 25 (25.5%) | *98* |  |
| South Tyrol | | 58 (47.2%) | | | | | 36 (29.3%) | | | | | 29 (23.6%) | *123* |  |
| Others ^§^ | | 6 (33.3%) | | | | | 8 (44.4%) | | | | | 4 (22.2%) | *18* |  |
| **… as a GP in the primary care setting** | | | | | | | | | | | | | | |
| Austria | | 112 (39.7%) | | | | | 112 (39.7%) | | | | | 58 (20.6%) | *282* | 0.104 ^ii^ |
| Germany | | 38 (39.2%) | | | | | 36 (37.1%) | | | | | 23 (23.7%) | *97* |  |
| South Tyrol | | 44 (35.8%) | | | | | 49 (39.8%) | | | | | 30 (24.4%) | *123* |  |
| Others ^§^ | | 2 (11.1%) | | | | | 7 (38.9%) | | | | | 9 (50.0%) | *18* |  |
| **… as a ward physician in a hospital** | | | | | | | | | | | | | | |
| Austria | | 53 (18.7%) | | | | | 178 (62.9%) | | | | | 52 (18.4%) | *283* | **0.036** ^ii^ |
| Germany | | 28 (28.6%) | | | | | 49 (50.0%) | | | | | 21 (21.4%) | *98* |  |
| South Tyrol | | 30 (24.4%) | | | | | 62 (51.2%) | | | | | 30 (24.4%) | *123* |  |
| Others ^§^ | | 1 (5.6%) | | | | | 10 (55.6%) | | | | | 7 (38.9%) | *18* |  |
| **… as a school physician** | | | | | | | | | | | | | | |
| Austria | | 37 (13.1%) | | | | | 212 (74.9%) | | | | | 34 (12.0%) | *283* | 0.423 ^ii^ |
| Germany | | 7 (7.1%) | | | | | 77 (78.6%) | | | | | 14 (14.3%) | *98* |  |
| South Tyrol | | 16 (13.0%) | | | | | 95 (77.2%) | | | | | 12 (9.8%) | *123* |  |
| Others ^§^ | | 3 (16.7%) | | | | | 15 (83.3%) | | | | | 0 (0.0%) | *18* |  |
| **… as a public health officer** | | | | | | | | | | | | | | |
| Austria | | 30 (10.7%) | | | | | 219 (77.9%) | | | | | 32 (11.4%) | *281* | 0.206 ^ii^ |
| Germany | | 8 (8.2%) | | | | | 74 (75.5%) | | | | | 16 (16.3%) | *98* |  |
| South Tyrol | | 21 (17.2%) | | | | | 89 (73.0%) | | | | | 12 (9.8%) | *122* |  |
| Others ^§^ | | 2 (11.1%) | | | | | 12 (66.7%) | | | | | 4 (22.2%) | *18* |  |
| **… as a physician with insurance companies or health insurances** | | | | | | | | | | | | | | |
| Austria | | 13 (4.6%) | | | | | 245 (86.6%) | | | | | 25 (8.8%) | *283* | 0.982 ^ii^ |
| Germany | | 4 (4.1%) | | | | | 84 (85.7%) | | | | | 10 (10.2%) | *98* |  |
| South Tyrol | | 4 (3.3%) | | | | | 106 (86.2%) | | | | | 13 (10.6%) | *123* |  |
| Others ^§^ | | 1 (5.6%) | | | | | 16 (88.9%) | | | | | 1 (5.6%) | *18* |  |
| **… in a scientific career** | | | | | | | | | | | | | | |
| Austria | | 84 (29.8%) | | | | | 152 (53.9%) | | | | | 46 (16.3%) | *282* | 0.272 ^ii^ |
| Germany | | 31 (31.6%) | | | | | 51 (52.0%) | | | | | 16 (16.3%) | *98* |  |
| South Tyrol | | 40 (32.8%) | | | | | 62 (50.8%) | | | | | 20 (16.4%) | *122* |  |
| Others ^§^ | | 8 (44.4%) | | | | | 4 (22.2%) | | | | | 6 (33.3%) | *18* |  |
| **… in a non-medical career (e.g. pharmaceutical industry, economy)** | | | | | | | | | | | | | | |
| Austria | | 24 (8.5%) | | | | | 224 (79.7%) | | | | | 33 (11.7%) | *281* | 0.056 ^ii^ |
| Germany | | 8 (8.2%) | | | | | 78 (80.4%) | | | | | 11 (11.3%) | *97* |  |
| South Tyrol | | 11 (8.9%) | | | | | 96 (78.0%) | | | | | 16 (13.0%) | *123* |  |
| Others ^§^ | | 0 (0.0%) | | | | | 11 (61.1%) | | | | | 7 (38.9%) | *18* |  |
| **C8. I would like to work …** | | | | | | | | | | | | | | |
| **… in a rural area** | | | | | | | | | | | | | | |
| Austria | | 209 (73.9%) | | | | | 36 (12.7%) | | | | | 38 (13.4%) | *283* | 0.191 ^ii^ |
| Germany | | 60 (61.2%) | | | | | 16 (16.3%) | | | | | 22 (22.4%) | *98* |  |
| South Tyrol | | 88 (71.0%) | | | | | 18 (14.5%) | | | | | 18 (14.5%) | *124* |  |
| Others ^§^ | | 11 (57.9%) | | | | | 5 (26.3%) | | | | | 3 (15.8%) | *19* |  |
| **… in an urban area** | | | | | | | | | | | | | | |
| Austria | | 229 (80.6%) | | | | | 31 (10.9%) | | | | | 24 (8.5%) | *284* | 0.400 ^ii^ |
| Germany | | 74 (75.5%) | | | | | 12 (12.2%) | | | | | 12 (12.2%) | *98* |  |
| South Tyrol | | 101 (81.5%) | | | | | 9 (7.3%) | | | | | 14 (11.3%) | *124* |  |
| Others ^§^ | | 18 (94.7%) | | | | | 0 (0.0%) | | | | | 1 (5.3%) | *19* |  |

*GP* General Practitioner, *Exp* Experience

^ii^ Chi² Test

^§^ Other nationalities include: Italian regions except South Tyrol, EU-states, Non-EU-states

### **Supplementary Table S7b**: Nationality-related analyses regarding interest in general practice, feeling of preparedness to work as a GP, specialist qualification, and personal preferences regarding various professional profiles and working circumstances (part C of the questionnaire) – RESIDENTS

| **RESIDENTS** | | **Only general practice** | | | | **General practice or other specialty** | | **Only other specialty** | | **Don’t know** | ***n*** | **p-value** |
| --- | --- | --- | --- | --- | --- | --- | --- | --- | --- | --- | --- | --- |
| **C1. Currently planned specialty choice** | | | | | | | | | | | |  |
| Austria | | 9 (14.8%) | | | | 32 (52.5%) | | 20 (32.8%) | | 0 (0.0%) | *61* | **<0.001** ^ii^ |
| Germany | | 1 (6.3%) | | | | 6 (37.5%) | | 9 (56.3%) | | 0 (0.0%) | *16* |  |
| South Tyrol | | 1 (4.8%) | | | | 13 (61.9%) | | 7 (33.3%) | | 0 (0.0%) | *21* |  |
| Others ^§^ | | 1 (20.0%) | | | | 0 (0.0%) | | 2 (40.0%) | | 2 (40.0%) | *5* |  |
|  | | **Yes, surely** | | **Rather yes** | | | **Neutral** | | **Rather no** | **Not at all** | ***n*** | **p-value** |
| **C2. Interest in general practice as a specialty for the future professional life** | | | | | | | | | | | |  |
| Austria | | 18 (29.5%) | | 15 (24.6%) | | | 12 (19.7%) | | 10 (16.4%) | 6 (9.8%) | *61* | 0.645 ^ii^ |
| Germany | | 7 (46.7%) | | 2 (13.3%) | | | 2 (13.3%) | | 3 (20.0%) | 1 (6.7%) | *15* |  |
| South Tyrol | | 6 (28.6%) | | 7 (33.3%) | | | 3 (14.3%) | | 4 (19.0%) | 1 (4.8%) | *21* |  |
| Others ^§^ | | 1 (20.0%) | | 1 (20.0%) | | | 3 (60.0%) | | 0 (0.0%) | 0 (0.0%) | *5* |  |
|  | **Very good** | | **Good** | | **Neutral** | | **Bad** | | **Very bad** | **No exp.** | ***n*** | **p-value** |
| **C3a. Quality of the practical experiences in general practice during medical school** | | | | | | | | | | | | |
| Austria | 20 (32.8%) | | 21 (34.4%) | | 15 (24.6%) | | 2 (3.3%) | | 2 (3.3%) | 1 (1.6%) | *61* | 0.339 ^ii^ |
| Germany | 6 (37.5%) | | 6 (37.5%) | | 2 (12.5%) | | 2 (12.5%) | | 0 (0.0%) | 0 (0.0%) | *16* |  |
| South Tyrol | 7 (33.3%) | | 11 (52.4%) | | 3 (14.3%) | | 0 (0.0%) | | 0 (0.0%) | 0 (0.0%) | *21* |  |
| Others ^§^ | 3 (60.0%) | | 0 (0.0%) | | 1 (20.0%) | | 0 (0.0%) | | 1 (20.0%) | 0 (0.0%) | *5* |  |
| **C3b. Quality of the theoretical experiences in general practice during medical school** | | | | | | | | | | | | |
| Austria | 1 (1.6%) | | 9 (14.8%) | | 22 (36.1%) | | 20 (32.8%) | | 8 (13.1%) | 1 (1.6%) | *61* | 0.059 ^ii^ |
| Germany | 2 (12.5%) | | 5 (31.3%) | | 3 (18.8%) | | 5 (31.3%) | | 0 (0.0%) | 1 (6.3%) | *16* |  |
| South Tyrol | 0 (0.0%) | | 8 (38.1%) | | 9 (42.9%) | | 2 (9.5%) | | 2 (9.5%) | 0 (0.0%) | *21* |  |
| Others ^§^ | 1 (20.0%) | | 0 (0.0%) | | 3 (60.0%) | | 1 (20.0%) | | 0 (0.0%) | 0 (0.0%) | *5* |  |
|  | | **Yes, surely** | | **Rather yes** | | | **Neutral** | | **Rather no** | **Not at all** | ***n*** | **p-value** |
| **C4a. I feel well prepared to work as a GP by medical school** | | | | | | | | | | | | |
| Austria | | 2 (3.3%) | | 3 (4.9%) | | | 9 (14.8%) | | 29 (47.5%) | 18 (29.5%) | *61* | **0.026** ^ii^ |
| Germany | | 0 (0.0%) | | 0 (0.0%) | | | 5 (31.3%) | | 9 (56.3%) | 2 (12.5%) | *16* |  |
| South Tyrol | | 0 (0.0%) | | 2 (9.5%) | | | 6 (28.6%) | | 13 (61.9%) | 0 (0.0%) | *21* |  |
| Others ^§^ | | 0 (0.0%) | | 0 (0.0%) | | | 4 (80.0%) | | 1 (20.0%) | 0 (0.0%) | *5* |  |
| **C4b. I feel well prepared to work as a GP by the general practice training** | | | | | | | | | | | | |
| Austria | | 5 (8.3%) | | 25 (41.7%) | | | 14 (23.3%) | | 12 (20.0%) | 4 (6.7%) | *60* | 0.293 ^ii^ |
| Germany | | 1 (6.7%) | | 6 (40.0%) | | | 2 (13.3%) | | 3 (20.0%) | 3 (20.0%) | *15* |  |
| South Tyrol | | 4 (20.0%) | | 7 (35.0%) | | | 6 (30.0%) | | 3 (15.0%) | 0 (0.0%) | *20* |  |
| Others ^§^ | | 2 (40.0%) | | 1 (20.0%) | | | 2 (40.0%) | | 0 (0.0%) | 0 (0.0%) | *5* |  |
| **C5. A specialist qualification would increase the attractiveness of general practice** | | | | | | | | | | | | |
| Austria | | 14 (23.0%) | | 19 (31.3%) | | | 10 (16.4%) | | 12 (19.7%) | 6 (9.8%) | *61* | 0.769 ^ii^ |
| Germany | | 5 (31.3%) | | 7 (43.8%) | | | 1 (6.3%) | | 1 (6.3%) | 2 (12.5%) | *16* |  |
| South Tyrol | | 4 (19.0%) | | 8 (38.1%) | | | 4 (19.0%) | | 3 (14.3%) | 2 (9.5%) | *21* |  |
| Others ^§^ | | 2 (40.0%) | | 0 (0.0%) | | | 1 (20.0%) | | 2 (40.0%) | 0 (0.0%) | *5* |  |
|  | | **Yes, surely** | | **Rather yes** | | | **Neutral** | | **Rather no** | **Not at all** | ***n*** | **p-value** |
| **C6. A specialist qualification is more attractive to me than general practice, because …** | | | | | | | | | | | | |
| **… without any specialist qualification, I would be a ‘lesser’ physician** | | | | | | | | | | | | |
| Austria | | 6 (10.0%) | | 13 (21.7%) | | | 8 (13.3%) | | 17 (28.3%) | 16 (26.7%) | *60* | 0.611 ^ii^ |
| Germany | | 2 (12.5%) | | 2 (12.5%) | | | 3 (18.8%) | | 3 (18.8%) | 6 (37.5%) | *16* |  |
| South Tyrol | | 3 (14.3%) | | 1 (4.8%) | | | 4 (19.0%) | | 8 (38.1%) | 5 (23.8%) | *21* |  |
| Others ^§^ | | 1 (20.0%) | | 0 (0.0%) | | | 2 (40.0%) | | 2 (40.0%) | 0 (0.0%) | *5* |  |
| **… I can get higher reputation as a specialist** | | | | | | | | | | | | |
| Austria | | 9 (15.3%) | | 17 (28.8%) | | | 10 (16.9%) | | 13 (22.0%) | 10 (16.9%) | *59* | **0.047** ^ii^ |
| Germany | | 1 (6.3%) | | 1 (6.3%) | | | 5 (31.3%) | | 2 (12.5%) | 7 (43.8%) | *16* |  |
| South Tyrol | | 2 (9.5%) | | 4 (19.0%) | | | 6 (28.6%) | | 8 (38.1%) | 1 (4.8%) | *21* |  |
| Others ^§^ | | 1 (20.0%) | | 3 (60.0%) | | | 0 (0.0%) | | 1 (20.0%) | 0 (0.0%) | *5* |  |
| **… teamwork is easier as a specialist** | | | | | | | | | | | | |
| Austria | | 7 (11.7%) | | 21 (35.0%) | | | 9 (15.0%) | | 17 (28.3%) | 6 (10.0%) | *60* | 0.104 ^ii^ |
| Germany | | 3 (18.8%) | | 4 (25.0%) | | | 2 (12.5%) | | 2 (12.5%) | 5 (31.3%) | *16* |  |
| South Tyrol | | 7 (33.3%) | | 1 (4.8%) | | | 6 (28.6%) | | 5 (23.8%) | 2 (9.5%) | *21* |  |
| Others ^§^ | | 1 (20.0%) | | 2 (40.0%) | | | 1 (20.0%) | | 1 (20.0%) | 0 (0.0%) | *5* |  |
| **… I have better possibilities to work in research** | | | | | | | | | | | | |
| Austria | | 8 (13.3%) | | 17 (28.3%) | | | 9 (15.0%) | | 14 (23.3%) | 12 (20.0%) | *60* | 0.918 ^ii^ |
| Germany | | 2 (12.5%) | | 3 (18.8%) | | | 5 (31.3%) | | 3 (18.8%) | 3 (18.8%) | *16* |  |
| South Tyrol | | 4 (19.0%) | | 6 (28.6%) | | | 2 (9.5%) | | 5 (23.8%) | 4 (19.0%) | *21* |  |
| Others ^§^ | | 1 (20.0%) | | 1 (20.0%) | | | 0 (0.0%) | | 1 (20.0%) | 2 (40.0%) | *5* |  |
| **… I have a higher income** | | | | | | | | | | | | |
| Austria | | 19 (32.2%) | | 15 (25.4%) | | | 9 (15.3%) | | 12 (20.3%) | 4 (6.8%) | *59* | 0.472 ^ii^ |
| Germany | | 2 (12.5%) | | 8 (50.0%) | | | 3 (18.8%) | | 1 (6.3%) | 2 (12.5%) | *16* |  |
| South Tyrol | | 7 (33.3%) | | 3 (14.3%) | | | 6 (28.6%) | | 3 (14.3%) | 2 (9.5%) | *21* |  |
| Others ^§^ | | 2 (40.0%) | | 2 (40.0%) | | | 0 (0.0%) | | 1 (20.0%) | 0 (0.0%) | *5* |  |
| **… there are clearer content-related demarcations of the field** | | | | | | | | | | | | |
| Austria | | 14 (23.3%) | | 18 (30.0%) | | | 19 (31.7%) | | 6 (10.0%) | 3 (5.0%) | *60* | 0.498 ^ii^ |
| Germany | | 2 (12.5%) | | 6 (37.5%) | | | 2 (12.5%) | | 4 (25.0%) | 2 (12.5%) | *16* |  |
| South Tyrol | | 7 (33.3%) | | 8 (38.1%) | | | 4 (19.0%) | | 2 (9.5%) | 0 (0.0%) | *21* |  |
| Others ^§^ | | 2 (40.0%) | | 2 (40.0%) | | | 1 (20.0%) | | 0 (0.0%) | 0 (0.0%) | *5* |  |
| **… I am facing more challenging medical topics** | | | | | | | | | | | | |
| Austria | | 7 (11.7%) | | 8 (13.3%) | | | 24 (40.0%) | | 18 (30.0%) | 3 (5.0%) | *60* | **0.016** ^ii^ |
| Germany | | 2 (12.5%) | | 3 (18.8%) | | | 2 (12.5%) | | 3 (18.8%) | 6 (37.5%) | *16* |  |
| South Tyrol | | 5 (23.8%) | | 6 (28.6%) | | | 5 (23.8%) | | 4 (19.0%) | 1 (4.8%) | *21* |  |
| Others ^§^ | | 1 (20.0%) | | 0 (0.0%) | | | 2 (40.0%) | | 2 (40.0%) | 0 (0.0%) | *5* |  |
| **… I have better opportunities of career** | | | | | | | | | | | | |
| Austria | | 15 (25.0%) | | 22 (36.7%) | | | 9 (15.0%) | | 12 (20.0%) | 2 (3.3%) | *60* | **0.017** ^ii^ |
| Germany | | 2 (12.5%) | | 2 (12.5%) | | | 4 (25.0%) | | 3 (18.8%) | 5 (31.3%) | *16* |  |
| South Tyrol | | 5 (23.8%) | | 8 (38.1%) | | | 6 (28.6%) | | 1 (4.8%) | 1 (4.8%) | *21* |  |
| Others ^§^ | | 3 (60.0%) | | 1 (20.0%) | | | 0 (0.0%) | | 0 (0.0%) | 1 (20.0%) | *5* |  |
| **… I can extend my knowledge in a more targeted way** | | | | | | | | | | | | |
| Austria | | 16 (26.7%) | | 24 (40.0%) | | | 12 (20.0%) | | 6 (10.0%) | 2 (3.3%) | *60* | 0.506 ^ii^ |
| Germany | | 5 (31.3%) | | 3 (18.8%) | | | 4 (25.0%) | | 2 (12.5%) | 2 (12.5%) | *16* |  |
| South Tyrol | | 9 (42.9%) | | 9 (42.9%) | | | 2 (9.5%) | | 1 (4.8%) | 0 (0.0%) | *21* |  |
| Others ^§^ | | 2 (40.0%) | | 1 (20.0%) | | | 2 (40.0%) | | 0 (0.0%) | 0 (0.0%) | *5* |  |
|  | | **Yes** | | | | | **No** | | | **Don’t know** | ***n*** | **p-value** |
| **C7. I would preferably work …** | | | | | | | | | | | | |
| **… as an independent physician in a group office** | | | | | | | | | | | | |
| Austria | | 49 (83.1%) | | | | | 3 (5.1%) | | | 7 (11.9%) | *59* | 0.446 ^ii^ |
| Germany | | 10 (62.5%) | | | | | 2 (12.5%) | | | 4 (25.0%) | *16* |  |
| South Tyrol | | 17 (81.0%) | | | | | 1 (4.8%) | | | 3 (14.3%) | *21* |  |
| Others ^§^ | | 3 (60.0%) | | | | | 0 (0.0%) | | | 2 (40.0%) | *5* |  |
| **… as an independent physician in a single-handed office** | | | | | | | | | | | | |
| Austria | | 23 (38.3%) | | | | | 29 (48.3%) | | | 8 (13.3%) | *60* | 0.555 ^ii^ |
| Germany | | 5 (31.3%) | | | | | 8 (50.0%) | | | 3 (18.8%) | *16* |  |
| South Tyrol | | 4 (19.0%) | | | | | 12 (57.1%) | | | 5 (23.8%) | *21* |  |
| Others ^§^ | | 1 (20.0%) | | | | | 2 (40.0%) | | | 2 (40.0%) | *5* |  |
| **… as a physician in the hospital setting** | | | | | | | | | | | | |
| Austria | | 28 (45.9%) | | | | | 24 (39.3%) | | | 9 (14.8%) | *61* | 0.541 ^ii^ |
| Germany | | 10 (62.5%) | | | | | 3 (18.8%) | | | 3 (18.8%) | *16* |  |
| South Tyrol | | 12 (57.1%) | | | | | 4 (19.0%) | | | 5 (23.8%) | *21* |  |
| Others ^§^ | | 3 (60.0%) | | | | | 1 (20.0%) | | | 1 (20.0%) | *5* |  |
| **… as an employed physician in another physician’s office** | | | | | | | | | | | | |
| Austria | | 31 (51.7%) | | | | | 15 (25.0%) | | | 14 (23.3%) | *60* | 0.750 ^ii^ |
| Germany | | 10 (66.7%) | | | | | 3 (20.0%) | | | 2 (13.3%) | *15* |  |
| South Tyrol | | 11 (52.4%) | | | | | 6 (28.6%) | | | 4 (19.0%) | *21* |  |
| Others ^§^ | | 3 (60.0%) | | | | | 0 (0.0%) | | | 2 (40.0%) | *5* |  |
| **… as a GP in a multiprofessional team (e.g. Primary Healthcare Center)** | | | | | | | | | | | | |
| Austria | | 39 (65.0%) | | | | | 15 (25.0%) | | | 6 (10.0%) | *60* | 0.300 ^ii^ |
| Germany | | 7 (43.8%) | | | | | 5 (31.1%) | | | 4 (25.0%) | *16* |  |
| South Tyrol | | 15 (71.4%) | | | | | 4 (19.0%) | | | 2 (9.5%) | *21* |  |
| Others ^§^ | | 5 (100%) | | | | | 0 (0.0%) | | | 0 (0.0%) | *5* |  |
| **… as a GP in the primary care setting** | | | | | | | | | | | | |
| Austria | | 34 (56.7%) | | | | | 22 (36.7%) | | | 4 (6.7%) | *60* | 0.703 ^ii^ |
| Germany | | 9 (56.3%) | | | | | 5 (31.1%) | | | 2 (12.5%) | *16* |  |
| South Tyrol | | 9 (42.9%) | | | | | 8 (38.1%) | | | 4 (19.0%) | *21* |  |
| Others ^§^ | | 3 (60.0%) | | | | | 2 (40.0%) | | | 0 (0.0%) | *5* |  |
| **… as a ward physician in a hospital** | | | | | | | | | | | | |
| Austria | | 8 (13.3%) | | | | | 43 (71.7%) | | | 9 (15.0%) | *60* | 0.585 ^ii^ |
| Germany | | 0 (0.0%) | | | | | 12 (75.0%) | | | 4 (25.0%) | *16* |  |
| South Tyrol | | 1 (4.8%) | | | | | 15 (71.4%) | | | 5 (23.8%) | *21* |  |
| Others ^§^ | | 0 (0.0%) | | | | | 3 (75.0%) | | | 1 (25.0%) | *4* |  |
| **… as a school physician** | | | | | | | | | | | | |
| Austria | | 13 (21.7%) | | | | | 40 (66.7%) | | | 7 (11.7%) | *60* | 0.946 ^ii^ |
| Germany | | 2 (12.5%) | | | | | 11 (68.8%) | | | 3 (18.8%) | *16* |  |
| South Tyrol | | 4 (19.0%) | | | | | 14 (66.7%) | | | 3 (14.3%) | *21* |  |
| Others ^§^ | | 1 (25.0%) | | | | | 3 (75.0%) | | | 0 (0.0%) | *4* |  |
| **… as a public health officer** | | | | | | | | | | | | |
| Austria | | 7 (11.9%) | | | | | 41 (69.5%) | | | 11 (18.6%) | *59* | 0.703 ^ii^ |
| Germany | | 2 (13.3%) | | | | | 10 (66.7%) | | | 3 (20.0%) | *15* |  |
| South Tyrol | | 4 (19.0%) | | | | | 12 (57.1%) | | | 5 (23.8%) | *21* |  |
| Others ^§^ | | 2 (40.0%) | | | | | 2 (40.0%) | | | 1 (20.0%) | *5* |  |
| **… as a physician with insurance companies or health insurances** | | | | | | | | | | | | |
| Austria | | 7 (11.9%) | | | | | 45 (76.3%) | | | 7 (11.9%) | *59* | 0.903 ^ii^ |
| Germany | | 1 (6.3%) | | | | | 14 (87.5%) | | | 1 (6.3%) | *16* |  |
| South Tyrol | | 2 (9.5%) | | | | | 17 (81.0%) | | | 2 (9.5%) | *21* |  |
| Others ^§^ | | 0 (0.0%) | | | | | 3 (75.0%) | | | 1 (25.0%) | *4* |  |
| **… in a scientific career** | | | | | | | | | | | | |
| Austria | | 9 (15.0%) | | | | | 43 (71.7%) | | | 8 (13.3%) | *60* | 0.380 ^ii^ |
| Germany | | 3 (18.8%) | | | | | 11 (68.8%) | | | 2 (12.5%) | *16* |  |
| South Tyrol | | 7 (33.3%) | | | | | 11 (52.4%) | | | 3 (14.3%) | *21* |  |
| Others ^§^ | | 1 (20.0%) | | | | | 2 (40.0%) | | | 2 (40.0%) | *5* |  |
| **… in a non-medical career (e.g. pharmaceutical industry, economy)** | | | | | | | | | | | | |
| Austria | | 14 (23.3%) | | | | | 38 (63.3%) | | | 8 (13.3%) | *60* | 0.072 ^ii^ |
| Germany | | 2 (12.5%) | | | | | 12 (75.0%) | | | 2 (12.5%) | *16* |  |
| South Tyrol | | 0 (0.0%) | | | | | 14 (66.7%) | | | 7 (33.3%) | *21* |  |
| Others ^§^ | | 0 (0.0%) | | | | | 3 (60.0%) | | | 2 (40.0%) | *5* |  |
| **C8. I would like to work …** | | | | | | | | | | | | |
| **… in a rural area** | | | | | | | | | | | | |
| Austria | | 49 (81.7%) | | | | | 3 (5.0%) | | | 8 (13.3%) | *60* | **0.011** ^ii^ |
| Germany | | 12 (75.0%) | | | | | 2 (12.5%) | | | 2 (12.5%) | *16* |  |
| South Tyrol | | 19 (95.0%) | | | | | 0 (0.0%) | | | 1 (5.0%) | *20* |  |
| Others ^§^ | | 1 (25.0%) | | | | | 2 (50.0%) | | | 1 (25.0%) | *4* |  |
| **… in an urban area** | | | | | | | | | | | | |
| Austria | | 37 (60.7%) | | | | | 14 (23.0%) | | | 10 (16.4%) | *61* | 0.502 ^ii^ |
| Germany | | 13 (81.3%) | | | | | 0 (0.0%) | | | 3 (18.8%) | *16* |  |
| South Tyrol | | 13 (61.9%) | | | | | 3 (14.3%) | | | 5 (23.8%) | *21* |  |
| Others ^§^ | | 3 (60.0%) | | | | | 1 (20.0%) | | | 1 (20.0%) | *5* |  |

*GP* General Practitioner, *Exp* Experience

^ii^ Chi² Test

^§^ Other nationalities include: Italian regions except South Tyrol, EU-states, Non-EU-states
